# Internalizing and externalizing symptoms in individuals with neurofibromatosis type 1: a systematic review and meta-analysis

**DOI:** 10.1186/s13643-024-02749-0

**Published:** 2025-01-22

**Authors:** Dan Liu, Liyan Yu, Xian Wu, Julia Moreira, Benjamin Felipe Mujica, Elora Shelly Mukhopadhyay, Angelena Novotney, André B. Rietman, Yang Hou

**Affiliations:** 1 Department of Behavioral Sciences and Social Medicine, College of Medicine, Florida State University, 1115 West Call Street, Tallahassee, FL 32306-4300 USA; 2 Department of Biostatistics, College of Public Health, University of Kentucky, Lexington, USA; 3 Department of Child and Adolescent Psychiatry/Psychology, Erasmus Medical Centre - Sophia Children’s Hospital, Rotterdam, The Netherlands

**Keywords:** Systematic review, Meta-analysis, Neurofibromatosis, Internalizing problems, Externalizing problems, Predictors

## Abstract

**Background:**

Individuals with neurofibromatosis type 1 (NF1) frequently report psychosocial problems, among which internalizing and externalizing symptoms are the most poorly understood due to limited research and inconsistent evidence. This hinders the overall attendance of their psychosocial needs and has a major impact on their quality of life. Thus, this systematic review and meta-analysis was conducted to synthesize existing findings on the degree to which individuals with NF1 experience internalizing and externalizing symptoms, compared with the unaffected population, and explore moderators of the group disparities.

**Methods:**

Scopus, PsycINFO, Web of Science, PubMed, and ProQuest were searched from inception to March 26th, 2024, which identified 59 eligible studies (*N* of NF1 = 3182, mean ages 2.38 to 46.4 years). Hedges’ *g* was calculated for differences in internalizing and externalizing symptoms between the NF1 group and the unaffected controls. Study effect sizes were pooled using robust variance estimation and random-effects models. Moderators of group differences were tested using meta-regression.

**Results:**

Random-effects meta-analyses indicated that compared with unaffected controls, individuals with NF1 showed more severe depressive (*k* = 21; *g* = 0.43; 95% CI [0.21, 0.65]), anxiety (*k* = 24; *g* = 0.27; 95% CI [0.01, 0.54]), somatic (*k* = 27; *g* = 0.56; 95% CI [0.30, 0.83]), total internalizing (*k* = 75; *g* = 0.50; 95% CI [0.33, 0.67]), aggression (*k* = 33; *g* = 0.33; 95% CI [0.08, 0.58]), delinquency, (*k* = 37; *g* = 0.43; 95% CI [0.26, 0.60]), and total externalizing symptoms (*k* = 47; *g* = 0.24; 95% CI [0.13, 0.35]). Studies that included more participants with NF1 who had ADHD or a lower verbal IQ reported greater group disparities in total internalizing symptoms or aggression.

**Conclusions:**

Findings highlight the importance of promptly recognizing internalizing and externalizing symptoms in individuals with NF1 for timely interventions. Future research should identify predictors of internalizing and externalizing symptoms within the NF1 population to inform our knowledge and intervention development. Other implications for future research were also discussed.

**Systematic review registration:**

The study protocol of this meta-analysis was registered at PROSPERO (CRD42023478258).

**Supplementary Information:**

The online version contains supplementary material available at 10.1186/s13643-024-02749-0.

## Background

Neurofibromatosis type 1 (NF1) is an autosomal genetic condition that causes the development of tumors in the peripheral and central nervous system [1]. Incidence of NF1 is approximately 1 in 3000 individuals globally with some variations across the globe [1]. Individuals with NF1 experience a wide range of clinical conditions (e.g., benign and/or malignant tumors, café-au-lait macules, skin pigmentation, and bone abnormalities), and they often struggle with cognitive impairment (e.g., poor executive functioning, learning disabilities [2, 3]. Increasing evidence suggests that psychosocial problems are frequently found in individuals with NF1 [3, 4], but this complication is relatively poorly understood. In fact, guidelines for supervision and treatment of NF1 have only recently started to suggest attending to age-appropriate psychosocial needs of individuals with NF1, but noting a lack of consistent evidence to support this recommendation [5]. This is partly due to the uneven understanding of the variety of psychosocial problems that individuals with NF1 experience. In particular, internalizing and externalizing symptoms are less well understood than the more studied symptoms of autism spectrum disorder (ASD) and attention-deficit/hyperactivity disorder (ADHD) [3].

Internalizing and externalizing symptoms represent two major categories of mental health problems that are often comorbid and associated with a host of developmental and life outcomes including persistent mental disorders [6], poor economic status [7], and elevated mortality risk [8]. Internalizing symptoms consist of a group of inwardly directed emotional symptoms including depressive, anxiety, and somatic symptoms [7]. Externalizing symptoms consist of a group of outwardly directed behavioral symptoms including aggression and delinquency [7]. Research shows that individuals with NF1 may experience these symptoms more severely than individuals with certain other chronic conditions, including coronary artery disease and cancer [9], and compared to the general population, not selected based on specific health conditions [10]. The underlying biological mechanisms of internalizing and externalizing symptoms in the NF1 population continue to be incompletely understood. However, it is possible that they are related to NF1 associated neuropathological changes [3], based on evidence showing comparable levels of internalizing symptoms between individuals with NF1 and individuals with other chronic diseases (e.g., multiple sclerosis, diabetes) that are predisposed to intracranial pathology [9, 10]. For individuals with NF1, internalizing and externalizing symptoms will add to other NF1-related mental, cognitive, and physical challenges and further undermine their quality of life [11]. Thus, it is paramount to improve our understanding of internalizing and externalizing symptoms in the NF1 population to better help them.

To date, the extent to which individuals with NF1 experience internalizing and externalizing symptoms, as compared with the unaffected population, remains unclear. This is mostly due to the limited number of relevant research and discrepancies in findings comparing individuals with versus without NF1. For instance, individuals with NF1 indicated more severe internalizing symptoms (e.g., depressive and anxiety symptoms) than those without NF1 in some studies [12–14] but not in others [15–17]. Similarly, the discrepancies in externalizing symptoms (e.g., aggression, delinquency) were only found in some studies [12, 18] but not in others [13–15]. The inconsistency limits our understanding and holds back the development or refinement of interventions, slowing down our efforts to improve quality of life for individuals with NF1 [11]. Based on this, a rigorous synthesis of existing findings is critically needed for determining the discrepancies in internalizing and externalizing symptoms between individuals with versus without NF1 as well as for explaining the heterogeneity of findings across studies.

Thus far, three meta-analyses have synthesized existing findings regarding psychosocial problems in individuals with NF1. One meta-analysis focused on quality of life and found a lower mental health score (assessed with quality of life measures) in individuals with NF (both types 1 and 2) than those without [11]. However, the findings are not informative about how individuals with NF1 experience specific mental health symptoms. To improve mental health status of individuals with NF1, it is crucial to find out how each mental health domain is affected and its potential predictors. Another meta-analysis focused on social functions [19]. The third focused on ADHD symptoms [20]. These studies found more severe ASD and ADHD symptoms in individuals with versus without NF1 and identified some potential predictors of these symptoms. Moving beyond previous meta-analytic studies, the current systematic review and meta-analysis focuses specifically on internalizing and externalizing symptoms as well as their subdimensions.

It is also important to investigate why some studies found greater discrepancies in internalizing and externalizing symptoms between individuals with and without NF1 than others. This will greatly improve interpretations of study findings and help identify subgroups who experience more severe symptoms, to facilitate personalized interventions. Possible factors include sample characteristics, such as sample age [21], sex composition [22, 23], percentage of familial NF1 cases in the sample [24], severity of intellectual disability [3], and percentage of participants diagnosed with ASD or ADHD [21–24]. A number of methodological factors may also be related. Specifically, the informant of behavioral problems may affect study findings [10, 12, 25]. Other potential methodological factors include measures used to assess internalizing and externalizing symptoms [26] and the type of the comparison group (i.e., healthy community controls, unaffected siblings, or normative sample), even though each type of comparison group has its own advantages [27].

In sum, this systematic review and meta-analysis was conducted with two aims. Aim 1 was to determine the degree to which individuals with NF1 experience internalizing (i.e., depressive, anxiety, somatic, and total internalizing symptoms) and externalizing (i.e., aggression, delinquency, and total externalizing symptoms) symptoms as compared with an unaffected control group (i.e., healthy community, unaffected siblings, or normative sample). Aim 2 was to test potential moderators of group differences across studies, including sample characteristics (i.e., age, sex, NF1 transmission, intelligence quotient or IQ, ASD diagnosis, and ADHD diagnosis) and methodological factors (i.e., informant, measure, and control group type).

## Methods

This meta-analysis was registered at PROSPERO (CRD42023478258). The reporting of this meta-analysis closely followed the guidelines of the Preferred Reporting Items for Systematic Reviews and Meta-analyses (PRISMA; see Online Resource 2) [28].

### Data sources and search strategy

This is one of a series of systematic reviews on neurobehavioral functioning (e.g., socioemotional and behavioral functioning, academic functioning, cognitive functioning) of individuals with NF1. Literature searches were conducted in Scopus, Web of Science, PsycINFO, PubMed, and ProQuest Dissertations & Theses Global with a combination of NF1 terms (e.g., neurofibromatosis type 1, NF1) and neurobehavioral functioning terms (e.g., internaliz*, externaliz*, depress*, aggress*, delinquen*). The complete search syntax is in Online Resource 3. The initial searches were conducted on September 22, 2022, which identified 4060 records. Additional searches were conducted to identify relevant papers since 2022 using the same search strategies on March 26th, 2024. A total of 1483 records were generated in the supplemental searches.

### Study selection

The inclusion criteria of the larger systematic review project are listed in Online Resource 4. For the current study, eligible studies must have reported data on internalizing or externalizing problems in individuals with NF1 as well as a normal control group (i.e., healthy community group, unaffected siblings group) or have provided standardized scores (i.e., T scores, standard scores, scaled scores, or *z* scores) for the NF1 group (Online Resource 5). Study titles, abstracts, and full texts were screened by two reviewers independently. A third reviewer resolved conflicts between the two reviewers and finalized the list of studies to be included.

### Data extraction

Extracted information included sample characteristics such as mean age, age range, percentage of females, race/ethnicity, percentage of familial NF1 cases, and percentage of participants diagnosed with ADHD or ASD. The control group type (i.e., healthy community, unaffected siblings, normative sample) was also extracted. Measurement information extracted included measure name, mean score, standard deviation, score direction (whether a higher score indicated worse symptoms), score type (e.g., T score, standard score), and informant (e.g., parent/caregiver, self, teacher). Data were extracted for both the NF1 and the control groups and by two coders independently. A third reviewer resolved discrepancies between the two coders and checked the extracted data for accuracy. Authors of articles that did not provide the needed information for data analysis were contacted at least twice to request for the missing information.

### Quality/certainty assessment

Five methodological factors, as outlined in the Adapted Newcastle–Ottawa Scale for Cross-Sectional Studies [29], that may bias estimates of group differences in internalizing and externalizing symptoms between individuals with and without NF1 were considered: Representativeness of the sample, sample size, ascertainment of exposure (measurement validity), comparability, and assessment of outcome. For representativeness of the sample, sensitivity analyses were conducted to test whether effect sizes differed before and after removing studies that excluded individuals with a psychiatric disorder (i.e., general psychiatric disorder, depression, anxiety, and ADHD). For sample size, more weight was given to studies with larger samples when synthesizing effect sizes across studies (see Online Resource 1 for details of analyses). Regarding measurement validity, whether effect sizes varied across measures of internalizing and externalizing symptoms was tested. For comparability, whether effect sizes varied across three control group types including healthy community, unaffected siblings, and normative data was analyzed. For assessment of outcome, whether effect sizes varied across informants (parent, self, or teacher) was tested. Additionally, meta-regression and subgroup analyses were conducted separately according to each type of informant for each outcome. These methodological factors of each included study are reported in Online Resources 6–13.

### Data analysis plan

Seven sets of meta-analyses were completed for each of the internalizing (i.e., depressive, anxiety, somatic, and total internalizing symptoms) and externalizing (i.e., aggression, delinquency problems, and total externalizing symptoms) variables. The studies included in each set of meta-analysis are listed in Online Resources 7–13. First, group differences in internalizing and externalizing symptoms between the NF1 and the control groups were calculated as Hedges’ *g*, given its sensitivity to small samples [30]. The magnitude of Hedges’* g* was interpreted as small (0.2), medium (0.5), or large (0.8) [30]. Three parameters of heterogeneity were estimated: One for the existence of between-study heterogeneity (*Q* statistic) and two for the extent of between-study heterogeneity (τ^2^: Variance of true effect sizes, and *I*^2^: The ratio of true heterogeneity to total variance across the observed effect sizes) [30]. Forest plots were created to present group differences for each variable in each study (one effect size for each study).

Following the calculation of group differences, meta-regression was used to test potential moderators accounting for variance in group differences across studies [31]. Next, subgroup analyses were conducted for categorical moderators using the ROBUMETA package in R [32], which implements a robust standard error estimation technique that could handle dependent effect sizes [33]. Finally, publication bias was evaluated. This was completed using meta-regression analyses that examined whether standard errors of effect sizes moderated study effect sizes [33]. Additional techniques employed included Egger’s tests [34] with funnel plot [35] and trim-and-fill analyses [36] using METAFOR in R [37, 38]. More details of data analysis are in Supplementary Method (Online Resource 1).

## Results

### Participant and study characteristics

**Fig. 1:**
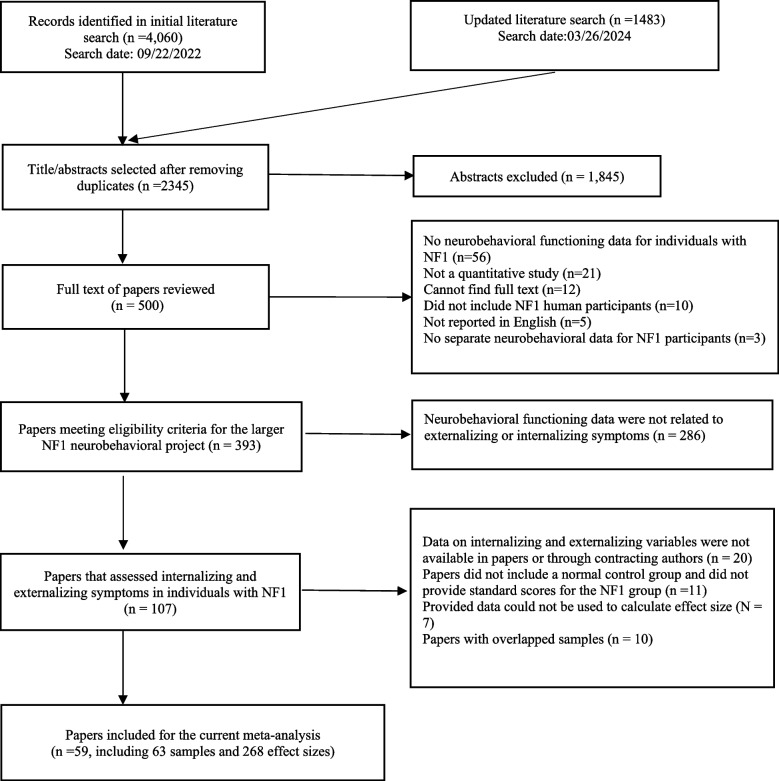
Flow diagram for the paper selection process in the current meta-analysis

The systematic searches identified 2345 unique articles, 107 of which examined internalizing and externalizing symptoms in individuals with NF1, and 59 papers provided sufficient data to calculate the effect size of group differences in internalizing and externalizing symptoms (Fig. 1). Some papers provided data for multiple unique samples (Online Resource 6), with each sample treated as a separate study in this meta-analysis [39]. As a result, the 59 papers reported on 63 studies, each with a unique sample, representing a total of 3182 individuals with NF1 (Online Resource 6). The mean age of the included NF1 samples ranged from 2.38 to 46.4 years, the full-scale IQ 83 to 105, the verbal IQ 86 to 112, and the performance IQ 82 to 103. The included NF1 samples had 20–86% females (one study included males only), 13–58% familial NF1 participants (27 studies reported the data, among which one included familial NF1 cases only and another, sporadic NF1 cases only), 8–77% diagnosed with ADHD (32 studies reported the data, among which five did not include any individuals with ADHD), and 10–71% diagnosed with ASD (12 studies reported the data, among which five did not include any individuals with ASD and two included only individuals with ASD). Due to the small number of studies that reported data on participants’ ASD diagnosis and the highly skewed distribution of the available data, ASD diagnosis data were not included in analysis.

Among the included studies, 59 (94%) were published journal articles, and four were unpublished dissertations [40–43]. Most studies included a small number of participants with NF1 (*N*s = 7–183): 57 (90%) studies included fewer than 100 participants with NF1. Most of the studies recruited participants with NF1 from the USA (*n* [the numer of studies] = 20, 32%)*,* followed by Australia (*n* = 8, 13%), Italy (*n* = 8, 13%), the Netherlands (*n* = 6, 10%), and others. Most of the studies included children (aged 0–18 years; *n* = 54, 86%); 14% of the studies (*n* = 9) included adults only (aged 19 years or older). Among the 268 effect sizes analyzed, the most used measure of internalizing and externalizing symptoms was the Child Behavior Checklist (CBCL; *k* [the number of effect sizes] = 168, 63%), followed by the Behavior Assessment System for Children (BASC; *k* = 60, 22%), and others (*k* = 40, 15%). Among the effect sizes, 179 (67%) were based on parent report, 38 (14%) self-report, and 49 (18%) teacher report. For control group type, 77 (29%) were composed of healthy community individuals, 32 (12%) unaffected siblings, and 159 (59%) based on normative data.

### Internalizing symptoms in individuals with versus without NF1

**Fig. 2:**
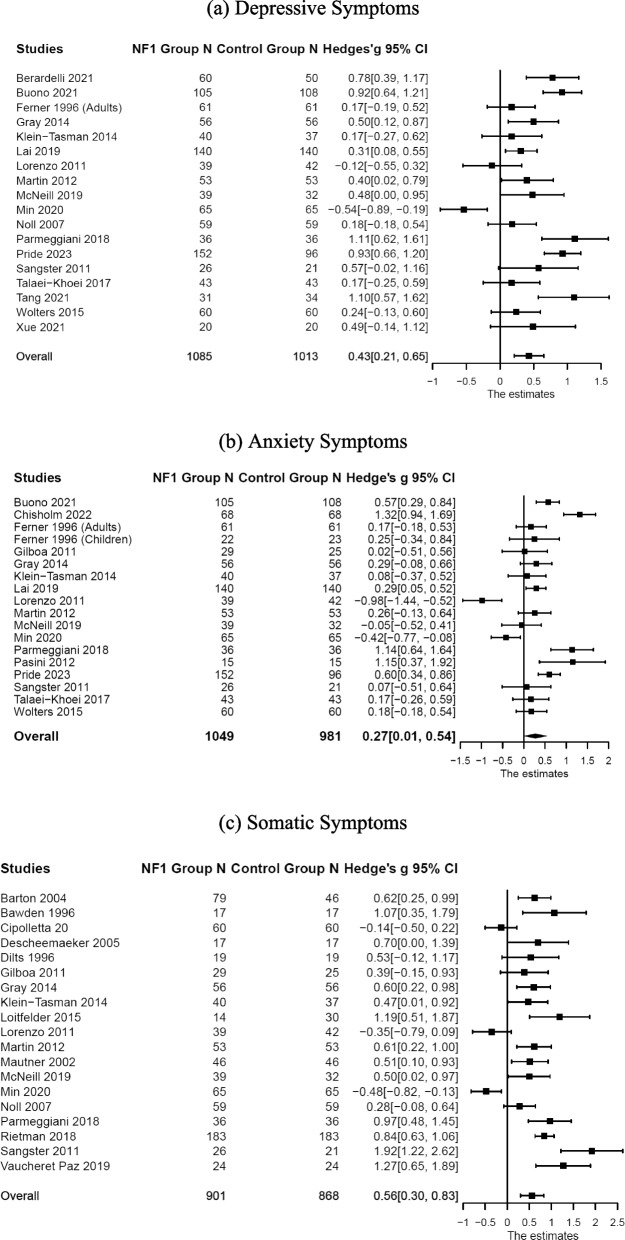
Forest plot for effect sizes of **a** depressive, **b** anxiety, and **c** somatic symptoms. Study labels are composed of first author’s last name and year of publication; for studies that had subgroups of NF1 participants and in which only subgroup data were used in analysis, study labels also include the NF1 subgroup name as labeled in each study. N = sample size. CI = confidence interval

**Fig. 3:**
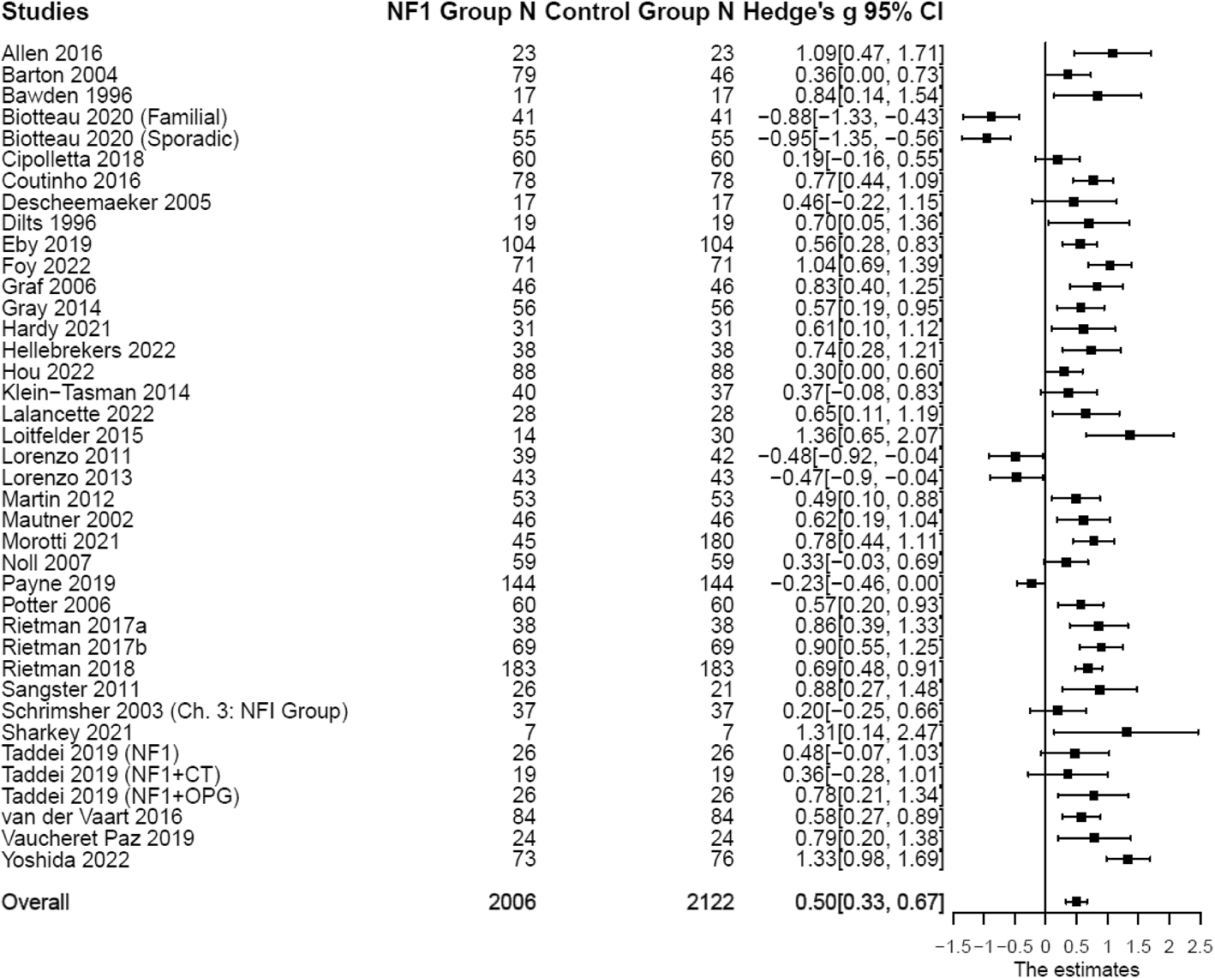
Forest plot for effect sizes of total internalizing symptoms. Study labels are composed of first author’s last name and year of publication; for studies that had subgroups of NF1 participants and in which only subgroup data were used in analysis, study labels also include the NF1 subgroup name as labeled in each study. N = sample size. CI = confidence interval

Compared with the control groups (Table 1), individuals with NF1 showed higher levels of depressive symptoms: *n* = 18; *k* = 21; *g* [effect size Hedge’s *g*] = 0.43, 95% CI [0.23, 0.63], *p* < 0.001; anxiety symptoms: *n* = 18; *k* = 24; *g* = 0.28, 95% CI [0.03, 0.52], *p* = 0.029; somatic symptoms: *n* = 19; *k* = 27; *g* = 0.57, 95% CI [0.32, 0.81], *p* < 0.001; and total internalizing symptoms: *n* = 39; *k* = 75; *g* = 0.50, 95% CI [0.33, 0.67], *p* < 0.001. Forest plots are presented in Figs. 2 and 3. Substantial systematic variability was observed in study effect sizes: Depressive symptoms: *Q*(17) = 86.51, *p* < 0.001, *T*^2^ = 0.15, *I*^2^ = 79.83; anxiety symptoms: *Q*(17) = 105.51, *p* < 0.001, *T*^2^ = 0.24, *I*^2^ = 86.12; somatic symptoms: *Q*(18) = 101.28, *p* < 0.001, *T*^2^ = 0.24, *I*^2^ = 83.33; and total internalizing symptoms: *Q*(38) = 238.32, *p* < 0.001, *T*^2^ = 0.22, *I*^2^ = 84.40.

**Table 1:** Summary of mean effect sizes across studies

	Hedges’ *g*	*LL*	*UL*	*SE*	*df*	*p* value for Hedges’ *g*	*n*	*k*	*Q*(df)	*p* value for *Q*	Tao^2^	*I*^2^ (%)
Depressive symptoms	0.43	0.23	0.63	0.10	16.80	< 0.001	18	21	86.51(17)	< 0.001	0.15	79.83
Anxiety symptoms	0.28	0.03	0.52	0.13	16.77	0.029	18	24	105.51(17)	< 0.001	0.24	86.12
Somatic symptoms	0.57	0.32	0.81	0.13	17.72	< 0.001	19	27	101.28(18)	< 0.001	0.24	83.33
Total internalizing symptoms	0.50	0.33	0.67	0.08	37.34	< 0.001	39	75	238.32(38)	< 0.001	0.22	84.40
Aggression	0.33	0.09	0.57	0.12	19.74	0.007	21	33	104.59(20)	< 0.001	0.24	82.88
Delinquency	0.43	0.27	0.59	0.08	23.05	< 0.001	25	37	72.25(24)	< 0.001	0.11	68.74
Total externalizing symptoms	0.24	0.14	0.35	0.05	29.23	< 0.001	33	47	59.51(32)	0.002	0.04	47.78

*Notes: LL* lower limit of 95% confidence interval, *UL* upper limit of 95% confidence interval, *SE* standard error, *df* degrees of freedom, *n* number of studies, *k *number of effect sizes, *Q* *Q* statistic, *df* degree of freedom, *Tao*^2^ Tau-squared, *I*^2 ^*I*-squared

Results from moderation analyses are presented in Online Resource 15 (see Online Resource 16 for results from subgroup analyses). Based on the results, the group difference in total internalizing symptoms was greater in studies with participants who had a lower mean verbal IQ (*β* = − 0.07, 95% CI [− 0.14, 0.00], *p* = 0.040). No other moderation effects were found.

### Externalizing symptoms in individuals with versus without NF1

**Fig. 4:**
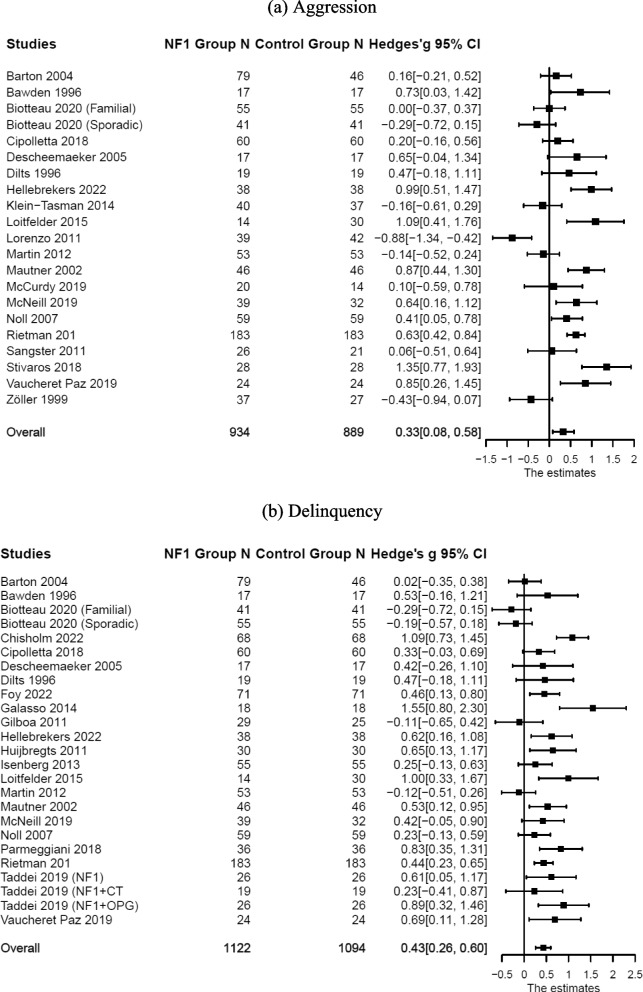
Forest plot for effect sizes of **a** aggression and **b** delinquency. Study labels are composed of first author’s last name and year of publication; for studies that had subgroups of NF1 participants and in which only subgroup data were used in analysis, study labels also include the NF1 subgroup name as labeled in each study. N = sample size. CI = confidence interval

**Fig. 5:**
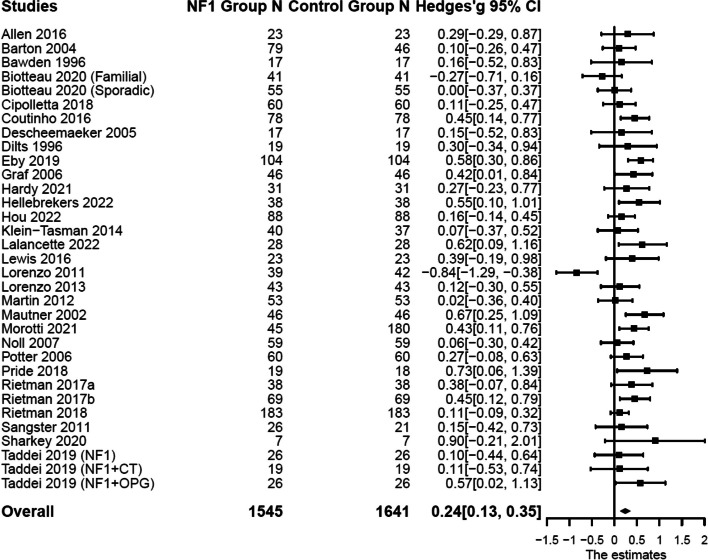
Forest plot for effect sizes of total externalizing symptoms. Study labels are composed of first author’s last name and year of publication; for studies that had subgroups of NF1 participants and in which only subgroup data were used in analysis, study labels also include the NF1 subgroup name as labeled in each study. N = sample size. CI = confidence interval

Compared with the control groups (Table 1), individuals with NF1 also showed higher levels of aggression: *n* = 21; *k* = 33; *g* = 0.33, 95% CI [0.09, 0.57], *p* = 0.007; delinquency: *n* = 25; *k* = 37; *g* = 0.43, 95% CI [0.27, 0.59], *p* < 0.001; and total externalizing symptoms: *n* = 33; *k* = 47; *g* = 0.24, 95% CI [0.14, 0.35], *p* < 0.001. Forest plots are presented in Figs. 4 and 5. Substantial systematic variability was observed in study effect sizes: Aggression: *Q*(20) = 104.59, *p* < 0.001, *T*^2^ = 0.24, *I*^2^ = 82.88; delinquency: *Q*(24) = 72.25, *p* < 0.001, *T*^2^ = 0.11, *I*^2^ = 68.74; total externalizing symptoms: *Q*(32) = 59.51, *p* = 0.002, *T*^2^ = 0.04, *I*^2^ = 47.78.

Based on moderation analyses (Online Resource 15; see Online Resource 16 for results from subgroup analyses), the percentage of NF1 participants diagnosed with ADHD moderated differences between the NF1 group and the control group in aggression: *β* = 0.02, 95% CI [0.00, 0.01], *p* = 0.017, with studies that had a higher percentage of NF1 participants diagnosed with ADHD reporting a larger group difference. The group difference in aggression was also larger in samples with lower mean verbal IQ: *β* = − 0.06, 95% CI [− 0.11, − 0.01], *p* = 0.028, and when the CBCL (*β* = 0.48, 95% CI [0.23, 0.74], *p* = 0.001) was used to measure aggression rather than the BASC (*β* = − 0.07, 95% CI [− 0.61, 0.46], *p* = 0.735): *β* = 0.57, 95% CI [0.04, 1.10], *p* = 0.039.

### Publication bias

Meta-regression with Egger’s test indicated no significant publication bias in studies that included depressive, anxiety, total internalizing, aggression, delinquency, and total externalizing symptoms. However, significant publication bias was observed in studies that included somatic symptoms (Online Resource 14). The funnel plots were largely consistent with these results as the effect sizes were symmetrically distributed around the average effect size for depressive, anxiety, total internalizing, aggression, delinquency, and total externalizing symptoms (Online Resources 17–18). Some asymmetrical distributions were found for somatic symptoms (Online Resource 17). The trim-and-fill analyses identified four hypothetical unpublished studies reporting somatic symptoms. After inputting these studies, the mean effect size became smaller but still statistically significant: *g* = 0.41; 95% CI [0.12, 0.69], *p* = 0.008.

### Sensitivity analysis

Sensitivity analyses were conducted to compare results with and without the four studies that excluded participants with a psychiatric disorder [17, 44–46]. The significance level, magnitude of effect sizes, and publication bias evaluation results were largely consistent between the two sets of analyses, except for somatic symptoms. In particular, after removing studies that had potentially biased sample selection [17, 45], the magnitude of the effect size of somatic symptoms changed from weak (Hedges’ *g* = 0.44) to medium (Hedges’ *g* = 0.57), and the effect size after adjusting for publication bias changed from Hedges’ *g* = 0.28 (*p* = 0.082) to Hedges’ *g* = 0.41 (*p* = 0.008). Thus, results after removing studies that were potentially biased in sample selection were reported for somatic symptoms, while results based on all eligible studies were reported for other internalizing and externalizing variables. Additional sensitivity analyses were conducted to test if results differed with and without unpublished dissertations [40–43]. The results of pooled effect sizes and moderation analyses were consistent with and without the four dissertations. Thus, results from analyses that included the four dissertations were reported, to provide a more thorough synthesis of existing literature.

## Discussion

The severity and heterogeneity of internalizing and externalizing symptoms in individuals with NF1 remain unclear given the inconsistent findings across the limited number of studies. This systematic review and meta-analysis synthesized existing findings and tested the extent to which individuals with NF1 experience internalizing and externalizing symptoms compared with those without NF1. Moderators of group differences were also tested to explore potential correlates of internalizing and externalizing symptoms in individuals with NF1. The meta-analyses included 59 studies, 63 unique samples, and 3182 individuals with NF1. Several important findings emerged.

First and foremost, findings suggest that individuals with NF1 experience more severe internalizing and externalizing symptoms than the unaffected comparison groups. Some variations in sizes of the group differences were also observed across domains of internalizing and externalizing symptoms (Hedges’ *g*s = 0.24–0.57). For instance, the group difference in somatic (Hedge’s *g* = 0.57) and total internalizing symptoms (Hedge’s *g* = 0.50) was more than twice the size as the group difference in total externalizing symptoms (Hedge’s *g* = 0.24). This is consistent with previous assessments indicating that internalizing symptoms might be more severe than externalizing symptoms in individuals with NF1 in terms of both prevalence and severity of the symptoms [3, 4, 47]. The observed sizes of group differences are likely smaller than the group differences observed for other more established phenotypes of NF1 including cognitive deficits [48, 49], ASD [19], and ADHD [50]. However, it is critical to recognize and treat internalizing and externalizing symptoms, as they all remained statistically significant even after adjusting for publication bias. NF1 complications are known to increase and worsen over time, while no cure for the disease has been found [3]. This often makes individuals with NF1 feel insecure and uncertain about the course of the disease, increasing risks for internalizing and externalizing symptoms [51]. Thus, timely identification and treatment of the problems as well as continued support will benefit individuals with NF1 tremendously.

In addition, the magnitude of group differences varied across study samples, and a number of study characteristics were related. First, the group differences in aggression were larger in studies that included a higher percentage of individuals diagnosed with ADHD in the NF1 group. This is consistent with the available research on individuals with NF1 that found a strong correlation between ADHD symptoms with externalizing symptoms [52], although the underlying causes remain unclear. In the general population, ADHD symptoms often covary with internalizing and externalizing symptoms [53], suggesting potential common underlying genetic and environmental factors. Future research should further test whether the covariations also exist among individuals with NF1 to confirm comorbidity of ADHD with externalizing symptoms in this population.

Second, group differences in total internalizing and aggression symptoms were larger in samples that had a lower verbal IQ. Language skills are frequently found to be linked to internalizing and externalizing symptoms in children without NF1 [54]. Abundant evidence shows that language use or skills play an important role in regulating emotions and behaviors [55–57], which is then associated with the degree of internalizing and externalizing symptoms across developmental periods [58, 59]. The inability to communicate efficiently and the associated low self-concept and poor social skills might also directly affect individuals’ internalizing symptoms [55]. Given the close associations between language skills with internalizing and externalizing symptoms, interventions have targeted language skills in young children to improve their internalizing and externalizing symptoms, and these interventions did produce promising results [60, 61]. Based on this evidence, intervening in verbal or general language skills of individuals with NF1 might also help decrease their internalizing and externalizing symptoms, a target missing in current interventions that focus primarily on the interactions between the mind and the body [62].

Moreover, findings suggest that the levels of externalizing symptoms (i.e., aggression) might be related to the measure used. Specifically, the difference in aggression between individuals with and without NF1 was found to be more prominent when measured by the CBCL than the BASC, although such a difference was not found for other internalizing or externalizing symptoms. However, our study cannot tell whether this difference was due to the sensitivity of the measures or due to other study or sample characteristics. Perhaps future research with individual-level data, where participants fill out both the CBCL and the BASC, can better compare the two measures and test how individual characteristics are related to differences in the CBCL versus the BASC scores.

In general, despite increasing evidence that suggests elevated internalizing and externalizing symptoms in individuals with NF1, there is a lack of research on the predictors. In addition to the factors discussed above, evidence shows that internalizing and externalizing symptoms in individuals with NF1 may be associated with social and demographic variables, such as age and parental education [47], as well as NF1-related disease factors, such as visibility and severity of NF1 [63, 64]. Preliminary evidence suggests that underlying neuropathological changes associated with NF1 may influence individuals’ psychological conditions [3]. Additionally, the multiple neurodevelopmental phenotypes individuals with NF1 experience (e.g., cognitive disability, ADHD and ASD symptoms, motor problems) may provide additive risk for them to develop internalizing symptoms [3, 65]. Other possible but understudied factors include insecurity or uncertainty about the course of NF1 and hopelessness over the lack of cure for NF1 [51], which can be depressing. Fatigue is another understudied factor that is a serious and frequent complication of NF1 [66] and often found to be related to internalizing symptoms in the general population [67]. Furthermore, internalizing and externalizing symptoms might be jointly affected by biopsychosocial factors (e.g., genetic predisposition, NF1 disease complications, social support, coping strategies) [68]. Much remains to be learned about what factors may contribute to internalizing and externalizing symptoms in individuals with NF1. Future research is critically needed to address this gap to improve our knowledge and inform interventions.

## Limitations

Although the current meta-analysis was conducted with robust methods following the most cutting-edge guidelines, several limitations should be considered in interpreting the results. Firstly, out of the 107 studies that focused on internalizing and externalizing symptoms in individuals with NF1, only 59 studies (including 63 independent samples) provided sufficient data for meta-analysis, even after multiple contacts with authors to request missing information. Thus, some moderation tests were potentially underpowered. To address this issue, it will be important for future studies to report study and sample characteristics in more detail. Related to this, the current study only tested a limited number of moderators or potential predictors of internalizing and externalizing symptoms in individuals with NF1, based on findings and data availability of existing research. Thus, factors that were not tested before or prior studies did not provide sufficient data for were not considered, such as fatigue and worry about future health, which should be addressed in future research as well.

Secondly, most of the included studies used a child sample rather than an adult sample (86% vs. 14%). In fact, adults with NF1 may have more pronounced mental health problems, particularly internalizing symptoms [3]. Thus, more future research on adults with NF1 is needed for a better understanding of the life-span experience of internalizing and externalizing symptoms among individuals with NF1. Moreover, the current meta-analysis included study-level data instead of individual-level data. Individual-level data will provide better information for testing factors related to internalizing and externalizing symptoms as well as enable the test of covariation among internalizing symptoms, externalizing symptoms, and ADHD symptoms. However, most existing studies with individual-level data have utilized small samples, which have provided limited power for analyses and thus produced unstable results. Future research should make an effort to recruit a larger number of participants or to seek collaborations with other sites to obtain larger samples. Finally, the current meta-analysis did not have sufficient data to test whether the results differed across cultures or socioeconomic groups, an important question that should be addressed in future research.

## Conclusions

This systematic review and meta-analysis provides robust evidence that individuals with NF1 experience a wide range of internalizing symptoms (depressive, anxiety, somatic, and total internalizing symptoms) and externalizing symptoms (aggression, delinquency, and total externalizing symptoms) more severely, as compared with the unaffected controls. This evidence supports the inclusion of psychosocial needs in the supervision and treatment of NF1 [5] and highlights the importance of early identification as well as continued support and treatment of internalizing and externalizing symptoms in individuals with NF1. This meta-analysis also found that a number of study characteristics (e.g., a higher percentage of participants diagnosed with ADHD, a lower sample mean verbal IQ) were related to worse internalizing and externalizing symptoms (total internalizing symptoms and aggression) observed in some study samples. These findings help to explain the heterogeneity of inconsistent discrepancies in internalizing and externalizing symptoms between individuals with versus without NF1 across studies. Additional research with individual-level data from a larger sample is still needed to better understand predictors of internalizing and externalizing symptoms among individuals with NF1. This research will further enhance our knowledge, inform existing interventions [62] and facilitate the development of new interventions or treatments.

## Supplementary information

Supplementary Material 1: Online Resource 1: Supplementary Method, Online Resource 2: PRISMA 2020 Checklist, Online Resource 3: Searching Syntax for the Larger NF1 Neurobehavioral Project, Online Resource 4: Inclusion and Exclusion Criteria for the Larger NF1 Neurobehavioral Project, Online Resource 5: Inclusion and Exclusion Criteria for the NF1 Internalizing/Externalizing Study, Online Resource 6: Characteristics of Studies Included in the Meta-Analysis, Online Resource 7: Characteristics of Studies Included in the Meta-Analysis of Depression, Online Resource 8: Characteristics of Studies Included in the Meta-Analysis of Anxiety, Online Resource 9: Characteristics of Studies Included in the Meta-Analysis of Somatic Complaints, Online Resource 10: Characteristics of Studies Included in the Meta-Analysis of Internalizing, Online Resource 11: Characteristics of Studies Included in the Meta-Analysis of Aggression, Online Resource 12: Characteristics of Studies Included in the Meta-Analysis of Delinquency, Online Resource 13: Characteristics of Studies Included in the Meta-Analysis of Externalizing, Online Resource 14: Sensitivity Analysis of Effect Size, Online Resource 15: Results from Moderation Tests, Online Resource 16: Mean Effects for Each Group of Categorical Moderators, Online Resource 17: Funnel Plots for Effect Sizes of Internalizing Problems, Online Resource 18: Funnel Plots for Effect Sizes of Externalizing Problems, References.

## Data Availability

Main data genderated in the current study are included in the supplementary document. Additional data can be provided upon request to the corresponding author.

## Abbreviations

ADHD: Attention-deficit/hyperactivity disorder
ASD: Autism spectrum disorder
BASC: Behavior Assessment System for Children
CBCL: Child Behavior Checklist
CI: Confidence intervals
Fig.: Figure
IQ: Intelligence quotient
NF: Neurofibromatosis
NF1: Neurofibromatosis type 1
PRISMA: The Preferred Reporting Items for Systematic Reviews and Meta-analyses
