# Supplementary material for: Internalizing and externalizing symptoms in individuals with neurofibromatosis type 1: a systematic review and meta-analysis
Source: Syst Rev. 2025 Jan 22;14:20. doi: 10.1186/s13643-024-02749-0 (PMC11752862; doi:10.1186/s13643-024-02749-0)
Supplement: Supplementary file 1 — Supplementary Material 1: Online Resource 1: Supplementary Method, Online Resource 2: PRISMA 2020 Checklist, Online Resource 3: Searching Syntax for the Larger NF1 Neurobehavioral Project, Online Resource 4: Inclusion and Exclusion Criteria for the Larger NF1 Neurobehavioral Project, Online Resource 5: Inclusion and Exclusion Criteria for the NF1 Internalizing/Externalizing Study, Online Resource 6: Characteristics of Studies Included in the Meta-Analysis, Online Resource 7: Characteristics of Studies Included in the Meta-Analysis of Depression, Online Resource 8: Characteristics of Studies Included in the Meta-Analysis of Anxiety, Online Resource 9: Characteristics of Studies Included in the Meta-Analysis of Somatic Complaints, Online Resource 10: Characteristics of Studies Included in the Meta-Analysis of Internalizing, Online Resource 11: Characteristics of Studies Included in the Meta-Analysis of Aggression, Online Resource 12: Characteristics of Studies Included in the Meta-Analysis of Delinquency, Online Resource 13: Characteristics of Studies Included in the Meta-Analysis of Externalizing, Online Resource 14: Sensitivity Analysis of Effect Size, Online Resource 15: Results from Moderation Tests, Online Resource 16: Mean Effects for Each Group of Categorical Moderators, Online Resource 17: Funnel Plots for Effect Sizes of Internalizing Problems, Online Resource 18: Funnel Plots for Effect Sizes of Externalizing Problems, References. [file 13643_2024_2749_MOESM1_ESM.docx]

**Internalizing and Externalizing Symptoms in Individuals with Neurofibromatosis Type 1: A Systematic Review and Meta-analysis**

***Systematic Reviews***

Dan Liu, Liyan Yu, Xian Wu, Julia Moreira, Benjamin Felipe Mujica, Elora Shelly Mukhopadhyay, Angelena Novotney, André B. Rietman, Yang Hou^*^

***Correspondence**: Dr. Yang Hou, Assistant Professor, Department of Behavioral Sciences and Social Medicine, College of Medicine, Florida State University. Email: yang.hou@med.fsu.edu.

**Table of Content**

[**Online Resource 1:** Supplementary Method 2](#_Toc177204928)

[**Online Resource 2**. PRISMA 2020 Checklist 6](#_Toc177204929)

[**Online Resource 3**. Searching Syntax for the Larger NF1 Neurobehavioral Project 9](#_Toc177204930)

[**Online Resource 4**. Inclusion and Exclusion Criteria for the Larger NF1 Neurobehavioral Project 11](#_Toc177204931)

[**Online Resource 5**. Inclusion and Exclusion Criteria for the NF1 Internalizing/Externalizing Study 12](#_Toc177204932)

[**Online Resource 6**. Characteristics of Studies Included in the Meta-Analysis 13](#_Toc177204933)

[**Online Resource 7**. Characteristics of Studies Included in the Meta-Analysis of Depressive Symptoms 16](#_Toc177204934)

[**Online Resource 8**. Characteristics of Studies Included in the Meta-Analysis of Anxiety Symptoms 18](#_Toc177204935)

[**Online Resource 9**. Characteristics of Studies Included in the Meta-Analysis of Somatic Symptoms 20](#_Toc177204936)

[**Online Resource 10.** Characteristics of Studies Included in the Meta-Analysis of Total Internalizing Symptoms 22](#_Toc177204937)

[**Online Resource 11**. Characteristics of Studies Included in the Meta-Analysis of Aggression 26](#_Toc177204938)

[**Online Resource 12**. Characteristics of Studies Included in the Meta-Analysis of Delinquency 28](#_Toc177204939)

[**Online Resource 13**. Characteristics of Studies Included in the Meta-Analysis of Total Externalizing Symptoms 30](#_Toc177204940)

[**Online Resource 14**. Sensitivity Analysis of Effect Size 33](#_Toc177204941)

[**Online Resource 15**. Results from Moderation Tests 34](#_Toc177204942)

[**Online Resource 16**. Mean Effects for Each Group of Categorical Moderators 36](#_Toc177204943)

[**Online Resource 17**. Funnel Plots for Effect Sizes of Internalizing Symptoms 38](#_Toc177204944)

[**Online Resource 18**. Funnel Plots for Effect Sizes of Externalizing Symptoms 39](#_Toc177204945)

[**References** 40](#_Toc177204946)

**Online Resource 1:** Supplementary Method

Seven sets of meta-analyses were conducted for depressive, anxiety, somatic, total internalizing, aggression, delinquency, and total externalizing symptoms. The studies included in each set of meta-analyses can be found in Online Resources 7-13. The statistical analysis was conducted in five steps. In Step 1, Hedges’ *g* for each effect size was calculated*.* The magnitude of Hedges’ *g* can be interpreted as small (0.2), medium (0.5), or large (0.8) (Cohen, 2013). In Step 2, effect sizes were pooled across studies. In Step 3, meta-regression was conducted to examine how study effect sizes varied across sample characteristics, measure informants, measures used, and different types of control groups used. In Step 4, subgroup analysis was conducted for categorical moderators. In Step 5, publication bias was evaluated. Overall effect size estimate was conducted only when there were at least four samples included (Fu et al., 2011). Meta-regression and/or subgroup analyses were conducted only when there were at least six samples for continuous variables and at least four samples in each subgroup of a categorical variable (Fu et al., 2011).

Steps 1 to 4 were completed using the ROBUMETA package in R (Fisher & Tipton, 2015), which employed robust variance estimation methods to handle dependency in effect sizes (Peng et al., 2018). In Step 5 (the examination of publication bias), meta-regression was conducted using the ROBUMETA package in R to examine whether the standard error of study effect size moderated study effect size (Fisher & Tipton, 2015; Peng et al., 2018). Egger’s Test (Peters et al., 2006), funnel plot (Amitay & Keinan-Boker, 2015), and Trim and Fill Analysis (Shi & Lin, 2019) using the METAFOR were also completed (Viechtbauer et al., 2015) after averaging multiple effect sizes in one study (van Geel et al., 2014). Details for each step and sample syntax are presented below.

**Step 1: Calculate Hedges’ g for the difference between the NF1 and the control groups.**

Two types of data were used for calculations: 1) mean and standard deviation (SD) of both the NF1 and the control groups, and 2) *t*-value (a ratio of the difference between means of the two groups) along with sample sizes for the NF1 and the control groups.

**a. Calculate Hedges’ *g* using mean and SD in R**

**First**, standard errors (SE) were calculated using the following formula (Higgins et al., 2019):

$$SE=SD/\sqrt{N}$$

*Note*. SD = standard deviation of the mean; N = sample size.

**Next**, the ‘esc_mean_se’ function in R was used to calculate Hedges’ *g*. Sample syntax is provided below:

Hedge_g = esc_mean_se (grp1m = mydata$NF1mean, grp1se = mydata$NF1se, grp1n = mydata$NF1n,

grp2m = mydata$CTRLmean, grp2se = mydata$CTRLse, grp2n = mydata$CTRLn,

es.type = “g”)

*Note.* NF1mean = mean score of the NF1 group; CTRLmean = mean score of the control group; NF1se = standard error of the NF1 group; CTRLse = standard error of the control group; NF1n = sample size of the NF1 group; CTRLn = sample size of the control group; es.type = type of effect size that should be returned was set to *g* = Hedges’ *g.*

Among the 59 papers included, 31 papers (including 35 independent samples) provided a standardized mean score (i.e., T score or Z score), SD, and sample size of the NF1 group but did not include a control group. For these studies, standardized normative means and SDs (normative T score: mean = 50, SD = 10; z score: mean = 0, SD =1) were used as the control group scores, and the sample size of the NF1 group was used as the sample size of the control group. Two papers included a subgroup of individuals with NF1 and a psychiatric disorder, and a subgroup of individuals with NF1 without a psychiatric disorder (Mautner et al., 2002; Zöller & Rembeck, 1999). We combined the means and standard deviations of the two subgroups using the following formula to get the relatively unbiased mean and SD of the total study sample (Altman et al., 2013; Higgins et al., 2019)

$$M=\frac{n1*m1+n2*m2}{n1+n2}$$

$$SD=\sqrt{\frac{(n1-1)*s1^{2}+(n2-1)*s2^{2}+\frac{n1*n2}{n1+n2}*(m1^{2}+m2^{2}-2m1*m2)}{n1+n2-1}}$$

*Note*. M = mean of the total group; SD = standardized deviation of the total group; n1 and n2 represent the number of observations in each group; s1 and s2 are the standard deviations of the two original groups; m1 and m2 are the means of the two original groups.

**b. Calculate Hedges’ *g* using t-value and sample size in R**

One study provided t ratios only between the NF1 and the control groups (Dilts et al., 1996). For this study, Hedges’ g was calculated using the ‘esc_t’ function in R. Sample syntax is provided below:

effect_t= esc_t (t = mydata$ t-value

grp1n = NF1n

grp2n = CTRLn

es.type=”g”)

*Note*. NF1n = sample size of the NF1 group; CTRLn = sample size of the control group; es.type = type of effect size that should be returned was set to *g* = Hedges’ *g.*

**Step 2: Calculate the Overall Hedges’ *g* Across Studies in R**

First, the variance (σ) of effect size (*g*) was obtained using the following formula (Hedges & Olkin, 2014; Lee, 2016):

$\sigma_{g}=\frac{n1+n2}{n1*n2}+\frac{g^{2}}{2\left( n1+n2 \right)}$

*Note.* n1 and n2 represent the number of observations in the NF1 and the control groups respectively; *g* = Hedges’ g; σ = variance of effect size.

Next, the overall effect size was calculated using the ‘robu’ function from the ‘robumeta’ package in R. Sample syntax is provided below:

overall_ext = robu (formula = Hedge_g.es ~ 1, data = mydata,

studynum = StudyID, var.eff.size = Hedge_g.var,

rho = .8, small = TRUE)

*Note.* studynum = a numeric or factor variable that uniquely identifies each study, var.eff.size = variance of effect size, rho = within-study effect-size correlation. The default value of rho is 0.8.

In addition, we computed the average effect size of each study and then calculated the overall effect size using METAFOR package with random-effects model (Viechtbauer et al., 2015; Viechtbauer & Viechtbauer, 2015). This method computed the overall effect size using inverse variance weights, giving more weight to studies with larger sample sizes or greater precision.

W_i_ = 1/$\sigma_{g}$_i_

*Note.* W_i_ = the weight for study I, $I$_i_ = the variance of the effect size estimate in study i.

A sample syntax is provided below:

rma_model <- rma (measure = “SMD”,

Hedge_g.es,

Hedge_g.var,

data = mydata)

rma_model

summary (rma_model)

*Note.* Hedge_g.var = effect size of each study, Hedge_g.var = variance of effect size.

To visually display the effect size for each study and its corresponding confidence interval, along with the overall effect size, we generated forest plots using the FORESTPLOT package (Gordon et al., 2019).

A sample syntax is provided below:

forestplot(labeltext = as.matrix(mydata[,1:6]),

mean = mydata$V7,

lower = mydata$V8,

upper = mydata$V9,

is.summary=c(T,T,T,F,F,F,F,T,F,F,T,F,F,F,T,F,F),

zero = 0,

boxsize = 0.4,

lineheight = unit(9,'mm'),

colgap = unit(7,'mm'),

lwd.zero = 2,

lwd.ci = 2,

col=fpColors(box='black',summary='black', lines = 'black',zero = 'black'),

xlab="The estimates",

lty.ci = "solid",

graph.pos = 4,

txt_gp=fpTxtGp(label=gpar(cex=1.25),

ticks=gpar(cex=1.1),

xlab=gpar(cex = 1.2),

title=gpar(cex = 1.2)))

*Note.* as.matrix = labeltext, mean = effect size, lower = the lower bound of the confidence interval for the forest plot, upper = the upper bound of the confidence interval for the forest plot, is.summary = a vector indicating by TRUE/FALSE if the value is a summary value which means that it will have a different font-style, zero = set reference value, boxsize = set the square size of point estimates, lineheight = set the line height in the graph, colgap = set the column gap in the graph, lwd.zero = set the thickness of the reference line, lwd.ci = set the thickness of the interval estimate line, col = define the colors of graphic elements, xlab = set the x-axis label, lty.ci = lty for the confidence bands, graph.pos = set the position of the forest plot, and txt_gp = set the fonts etc for all text elements.

**Step 3: Moderation analysis in R**

Given the results in step 2 that showed substantial systematic variability in the study effect sizes, moderator analysis was conducted using the ‘robu’ function from the ‘robumeta’ package in R. Sample syntax for testing moderation effects of a categorical variable (i.e., Control Group Type) is provided below:

model1<- robu (formula = Hedge_g.es ~ factor (ControlGroupType),

data = mydata, studynum = StudyID, var.eff.size = Hedge_g.var,

modelweights ="CORR", rho = .8, small = TRUE)

*Note*. studynum = a numeric or factor variable that uniquely identifies each study, var.eff.size = variance of effect size, Rho = within-study effect-size correlation

**Step 4: Subgroup analysis in R**

The overall effect size for each subgroup of every categorical moderator was calculated using the same function in step 2.

**Step 5: Examine Publication Bias in R**

**a. Meta-regression with standard errors of effect sizes as the predictor of study effect sizes**

Meta-regression analysis was conducted using the ‘robu’ function from the ‘robumeta’ package in R. This function required the effect size, the standard error and variance, and the study label/ID for each effect size. A significant association between standard error of the study effect size and the effect size suggests the presence of publication bias. Sample syntax is provided below:

bias_ext<- robu (formula = Hedge_g.es ~ Hedge_g.ste, data =mydata,

studynum = StudyID, var.eff.size = Hedge_g.var, modelweights="CORR",

rho = .8, small = TRUE)

*Note.* studynum = a numeric or factor variable that uniquely identifies each study, var.eff.size = variance of effect size, rho = within-study effect-size correlation. The default value of rho is 0.8.

**b. Egger's regression test**

Egger's regression test was conducted using the ‘regtest’ function with averaged effect size of each study. This function required the effect size and its variance as inputs. This function outputted the p-value of Z-value, where a p-value smaller than 0.05 indicated significant publication bias. Sample syntax is provided below:

rma_ext <- rma (measure = "SMD",

Hedge_g.es,

Hedge_g.var,

data = mydata)

regtest (rma_ext)

*Note.* rma_ext = the externalizing model; Hedge_g.var = effect size of each study, Hedge_g.var = variance of effect size.

**c. Funnel plot**

A funnel plot was created to visualize the distribution of effect size of each study following Egger’s test. An even distribution of effect sizes around the overall effect size indicates the absence of significant publication bias. Sample syntax is provided below:

Funnel (rma_ext, xlab = "Hedges’ g")

*Note*. rma_ext = the externalizing model; xlab = label of the x-axis.

**d. Trim-and-fill procedure**

Finally, the ‘metagen’ function was used to obtain the overall effect size of all the studies. This function required the effect size, its standard error, and the study label/ID of each study. Sample syntax is provided below:

m.hksj_ext <- metagen (Hedge_g.es,

Hedge_g.ste,

data = mydata,

studlab = paste (Group.1),

comb.fixed = FALSE,

comb.random = TRUE,

method.tau = “SJ",

hakn = TRUE,

prediction = TRUE,

sm = "SMD")

*Note*. m.hksj_ext = the externalizing model. Hedge_g.es = effect size of each study, Hedge_g.ste = standard error of effect size, studlab = study label, comb.fixed = whether a fixed effect meta-analysis will be conducted, comb.random = whether a random effect meta-analysis will be conducted, method.tau = which method is used to estimate the between-study variance, hakn = whether method by Hartung and Knapp should be used to adjust test statistics and confidence intervals, sm = The character string indicating underlying summary measure.

Additionally, the ‘trimfill’ function was used to identify hypothesized unpublished papers and estimate the adjusted overall effect size after filling these hypothesized missing studies. Sample syntax is provided below:

trimfill_Hyp_imp_PR<- trimfill(m.hksj_ext)

*Note*. m.hksj_ext = the externalizing model.

**Online Resource 2**. PRISMA 2020 Checklist

| **Section and Topic** | **Item #** | **Checklist item** | **Location where item is reported** |
| --- | --- | --- | --- |
| **TITLE** | | |  |
| Title | 1 | Identify the report as a systematic review. | p. 1 |
| **ABSTRACT** | | |  |
| Abstract | 2 | See the PRISMA 2020 for Abstracts checklist. | p. 2 |
| **INTRODUCTION** | | |  |
| Rationale | 3 | Describe the rationale for the review in the context of existing knowledge. | pp. 4-7 |
| Objectives | 4 | Provide an explicit statement of the objective(s) or question(s) the review addresses. | p. 7 |
| **METHODS** | | |  |
| Eligibility criteria | 5 | Specify the inclusion and exclusion criteria for the review and how studies were grouped for the syntheses. | p. 8 |
| Information sources | 6 | Specify all databases, registers, websites, organisations, reference lists and other sources searched or consulted to identify studies. Specify the date when each source was last searched or consulted. | pp. 7-8 |
| Search strategy | 7 | Present the full search strategies for all databases, registers and websites, including any filters and limits used. | Online Resource 3 |
| Selection process | 8 | Specify the methods used to decide whether a study met the inclusion criteria of the review, including how many reviewers screened each record and each report retrieved, whether they worked independently, and if applicable, details of automation tools used in the process. | p. 8 |
| Data collection process | 9 | Specify the methods used to collect data from reports, including how many reviewers collected data from each report, whether they worked independently, any processes for obtaining or confirming data from study investigators, and if applicable, details of automation tools used in the process. | p. 8 |
| Data items | 10a | List and define all outcomes for which data were sought. Specify whether all results that were compatible with each outcome domain in each study were sought (e.g. for all measures, time points, analyses), and if not, the methods used to decide which results to collect. | p. 8 |
|  | 10b | List and define all other variables for which data were sought (e.g. participant and intervention characteristics, funding sources). Describe any assumptions made about any missing or unclear information. | p. 8 |
| Study risk of bias assessment | 11 | Specify the methods used to assess risk of bias in the included studies, including details of the tool(s) used, how many reviewers assessed each study and whether they worked independently, and if applicable, details of automation tools used in the process. | pp. 9-10 |
| Effect measures | 12 | Specify for each outcome the effect measure(s) (e.g. risk ratio, mean difference) used in the synthesis or presentation of results. | pp. 9-10 |
| Synthesis methods | 13a | Describe the processes used to decide which studies were eligible for each synthesis (e.g. tabulating the study intervention characteristics and comparing against the planned groups for each synthesis (item #5)). | Online Resources 6-13 |
|  | 13b | Describe any methods required to prepare the data for presentation or synthesis, such as handling of missing summary statistics, or data conversions. | pp.9-10 |
|  | 13c | Describe any methods used to tabulate or visually display results of individual studies and syntheses. | pp. 9-10 |
|  | 13d | Describe any methods used to synthesize results and provide a rationale for the choice(s). If meta-analysis was performed, describe the model(s), method(s) to identify the presence and extent of statistical heterogeneity, and software package(s) used. | pp. 9-10 |
|  | 13e | Describe any methods used to explore possible causes of heterogeneity among study results (e.g. subgroup analysis, meta-regression). | p. 10 |
|  | 13f | Describe any sensitivity analyses conducted to assess robustness of the synthesized results. | pp. 9-10 |
| Reporting bias assessment | 14 | Describe any methods used to assess risk of bias due to missing results in a synthesis (arising from reporting biases). | pp. 9-10 |
| Certainty assessment | 15 | Describe any methods used to assess certainty (or confidence) in the body of evidence for an outcome. | p. 9 |
| **RESULTS** | | |  |
| Study selection | 16a | Describe the results of the search and selection process, from the number of records identified in the search to the number of studies included in the review, ideally using a flow diagram. | pp. 10-11, Fig. 1 |
|  | 16b | Cite studies that might appear to meet the inclusion criteria, but which were excluded, and explain why they were excluded. | Online Resource 5 |
| Study characteristics | 17 | Cite each included study and present its characteristics. | Online Resource 6 |
| Risk of bias in studies | 18 | Present assessments of risk of bias for each included study. | pp. 12-14, Online Resource 14 |
| Results of individual studies | 19 | For all outcomes, present, for each study: (a) summary statistics for each group (where appropriate) and (b) an effect estimate and its precision (e.g. confidence/credible interval), ideally using structured tables or plots. | Online Resources 7-13, Figs. 2-5 |
| Results of syntheses | 20a | For each synthesis, briefly summarise the characteristics and risk of bias among contributing studies. | pp. 12-14 |
|  | 20b | Present results of all statistical syntheses conducted. If meta-analysis was done, present for each the summary estimate and its precision (e.g. confidence/credible interval) and measures of statistical heterogeneity. If comparing groups, describe the direction of the effect. | pp. 12-14, Table 1. Online Resources 7-13, Online Resources 17-18 |
|  | 20c | Present results of all investigations of possible causes of heterogeneity among study results. | pp. 12-13, Online Resources 16-S17 |
|  | 20d | Present results of all sensitivity analyses conducted to assess the robustness of the synthesized results. | pp. 13-14 |
| Reporting biases | 21 | Present assessments of risk of bias due to missing results (arising from reporting biases) for each synthesis assessed. | p. 13, Online Resource 14 |
| Certainty of evidence | 22 | Present assessments of certainty (or confidence) in the body of evidence for each outcome assessed. | pp. 13-14 |
| **DISCUSSION** | | |  |
| Discussion | 23a | Provide a general interpretation of the results in the context of other evidence. | pp.14-17 |
|  | 23b | Discuss any limitations of the evidence included in the review. | pp.17-18 |
|  | 23c | Discuss any limitations of the review processes used. | pp.17-18 |
|  | 23d | Discuss implications of the results for practice, policy, and future research. | pp.15-19 |
| **OTHER INFORMATION** | | |  |
| Registration and protocol | 24a | Provide registration information for the review, including register name and registration number, or state that the review was not registered. | p. 7 |
|  | 24b | Indicate where the review protocol can be accessed, or state that a protocol was not prepared. | p. 7 |
|  | 24c | Describe and explain any amendments to information provided at registration or in the protocol. | pp. 9, 11 |
| Support | 25 | Describe sources of financial or non-financial support for the review, and the role of the funders or sponsors in the review. | Title page |
| Competing interests | 26 | Declare any competing interests of review authors. | Title page |
| Availability of data, code and other materials | 27 | Report which of the following are publicly available and where they can be found: template data collection forms; data extracted from included studies; data used for all analyses; analytic code; any other materials used in the review. | Title page,  Supplementary document |

**Online Resource 3**. Searching Syntax for the Larger NF1 Neurobehavioral Project

| **Scopus** |
| --- |
| (TITLE-ABS-KEY ( "neurofibromatosis 1" ) OR TITLE-ABS-KEY ( "neurofibromatosis1" ) OR TITLE-ABS-KEY ( "NF1" ) OR TITLE-ABS-KEY ( "NF 1" ) OR TITLE-ABS-KEY ( "neurofibromatosis type 1" ) OR TITLE-ABS-KEY ( "neurofibromatosis type1" ))  AND  ( TITLE-ABS-KEY ( "cogni*" ) OR TITLE-ABS-KEY ( "academi*" ) OR TITLE-ABS-KEY ( "socia*" ) OR TITLE-ABS-KEY ( "psycho*" ) OR TITLE-ABS-KEY ( "behavi*" ) OR TITLE-ABS-KEY ( "intelle*" ) OR TITLE-ABS-KEY ( "IQ" ) OR TITLE-ABS-KEY ( "executive function" ) OR TITLE-ABS-KEY ( "executive functioning" ) OR TITLE-ABS-KEY ( "attention" ) OR TITLE-ABS-KEY ( "inattention" ) OR TITLE-ABS-KEY ( "memory" ) OR TITLE-ABS-KEY ( "inhibitory control" ) OR TITLE-ABS-KEY ( "cognitive flexibility" ) OR TITLE-ABS-KEY ( "learning" ) OR TITLE-ABS-KEY ( "math" ) OR TITLE-ABS-KEY ( "reading" ) OR TITLE-ABS-KEY ( "writing" ) OR TITLE-ABS-KEY ( "language" ) OR TITLE-ABS-KEY ( "impuls*" ) OR TITLE-ABS-KEY ( "hyperact*" ) OR TITLE-ABS-KEY ( "ADHD" ) OR TITLE-ABS-KEY ( "emoti*" ) OR TITLE-ABS-KEY ( " internalizing symptoms" ) OR TITLE-ABS-KEY ( " externalizing symptoms" ) OR TITLE-ABS-KEY ( " internalizing problems" ) OR TITLE-ABS-KEY ( " externalizing problems" ) OR TITLE-ABS-KEY ( " anxiety" ) OR TITLE-ABS-KEY ( " depression" ) OR TITLE-ABS-KEY ( "aggression" ) OR TITLE-ABS-KEY ( " delinquency" ) OR TITLE-ABS-KEY ( "peer" ) OR TITLE-ABS-KEY ( "interpersonal" ) OR TITLE-ABS-KEY ( "empathy" ) OR TITLE-ABS-KEY ( "autis*" ) OR TITLE-ABS-KEY ( "ASD" ) OR TITLE-ABS-KEY ( " visual-spatial") OR TITLE-ABS-KEY ( "visual-motor" ) OR TITLE-ABS-KEY ( " visual spatial") OR TITLE-ABS-KEY ( "visual motor" ) )  AND NOT  ( TITLE-ABS-KEY ( "Animals" ) OR TITLE-ABS-KEY ( "mice " ) OR TITLE-ABS-KEY ( "mouse" ) OR TITLE-ABS-KEY ( "rat" ) OR TITLE-ABS-KEY ( "rats" ) OR TITLE-ABS-KEY ( "rodent" ) OR TITLE-ABS-KEY ( "dog" ) OR TITLE-ABS-KEY ( "dogs" ) OR TITLE-ABS-KEY ( "pig" ) OR TITLE-ABS-KEY ( "pigs" ) OR TITLE-ABS-KEY ( "piglet" ) OR TITLE-ABS-KEY ( "swine" ) OR TITLE-ABS-KEY ( "porcine" ) OR TITLE-ABS-KEY ( "animal" ) OR TITLE-ABS-KEY ( "fly" ) OR TITLE-ABS-KEY ( "Invertebrates" ) OR TITLE-ABS-KEY ( "rodentia" ) OR TITLE-ABS-KEY ( "models, animal" ) OR TITLE-ABS-KEY ( "Animal Experimentation" ) OR TITLE-ABS-KEY ( "editorial" ) OR TITLE-ABS-KEY ( "commentary" ) OR TITLE-ABS-KEY ( "conference abstract" ) OR TITLE-ABS-KEY ( "retraction of publication" ) OR TITLE-ABS-KEY ( "retracted publication" ) OR TITLE ( " systematic revie*" ) OR TITLE ( " meta-analy*" ) OR TITLE ( " protoco*" ) OR TITLE-ABS-KEY ( "case report" ) OR TITLE-ABS-KEY ( "case series" ) )  AND  ( LIMIT-TO ( SRCTYPE,"j" ) )  AND  ( LIMIT-TO ( DOCTYPE,"ar" ) OR LIMIT-TO ( DOCTYPE,"sh" ) ) AND  ( LIMIT-TO ( LANGUAGE,"English" ) ) |
| **Psycinfo** |
| ( AB( "neurofibromatosis 1" ) OR AB( "neurofibromatosis1" ) OR AB( "NF1" ) OR AB( "NF 1" ) OR AB( "neurofibromatosis type 1" ) OR AB( "neurofibromatosis type1" )) AND ( AB( "cogni*" ) OR AB( "academi*" ) OR AB( "socia*" ) OR AB( "psycho*" ) OR AB( "behavi*" ) OR AB( "intelle*" ) OR AB( "IQ" ) OR AB( "executive function" ) OR AB( "executive functioning" ) OR AB( "attention" ) OR AB( "inattention" ) OR AB( "memory" ) OR AB( "inhibitory control" ) OR AB( "cognitive flexibility" ) OR AB( "learning" ) OR AB( "math" ) OR AB( "reading" ) OR AB( "writing" ) OR AB( "language" ) OR AB( "impuls*" ) OR AB( "hyperact*" ) OR AB( "ADHD" ) OR AB( "emoti*" ) OR AB( " internalizing symptoms" ) OR AB( " externalizing symptoms" ) OR AB( " internalizing problems" ) OR AB( " externalizing problems" ) OR AB( " anxiety" ) OR AB( " depression" ) OR AB( "aggression" ) OR AB( " delinquency" ) OR AB( "peer" ) OR AB( "interpersonal" ) OR AB( "empathy" ) OR AB( "autis*" ) OR AB( "ASD" ) OR AB( " visual-spatial") OR AB( "visual-motor" ) OR AB( " visual spatial") OR AB( "visual motor" ) ) NOT ( AB( "Animals" ) OR AB( "mice " ) OR AB( "mouse" ) OR AB( "rat" ) OR AB( "rats" ) OR AB( "rodent" ) OR AB( "dog" ) OR AB( "dogs" ) OR AB( "pig" ) OR AB( "pigs" ) OR AB( "piglet" ) OR AB( "swine" ) OR AB( "porcine" ) OR AB( "animal" ) OR AB( "fly" ) OR AB( "Invertebrates" ) OR AB( "rodentia" ) OR AB( "models, animal" ) OR AB( "Animal Experimentation" ) OR AB( "editorial" ) OR AB( "commentary" ) OR AB( "conference abstract" ) OR AB( "retraction of publication" ) OR AB( "retracted publication" ) OR ti( " systematic revie*" ) OR ti( " meta-analy*" ) OR ti( " protoco*" ) OR AB( "case report" ) OR AB( "case series" ) )  DOCUMENT type limit to Article / Language limit to English |
| **Web of science** |
| (AB=( "neurofibromatosis 1" ) OR AB=( "neurofibromatosis1" ) OR AB=( "NF1" ) OR AB=( "NF 1" ) OR AB=( "neurofibromatosis type 1" ) OR AB=( "neurofibromatosis type1" )) AND (AB=( "cogni*" ) OR AB=( "academi*" ) OR AB=( "socia*" ) OR AB=( "psycho*" ) OR AB=( "behavi*" ) OR AB=( "intelle*" ) OR AB=( "IQ" ) OR AB=( "executive function" ) OR AB=( "executive functioning" ) OR AB=( "attention" ) OR AB=( "inattention" ) OR AB=( "memory" ) OR AB=( "inhibitory control" ) OR AB=( "cognitive flexibility" ) OR AB=( "learning" ) OR AB=( "math" ) OR AB=( "reading" ) OR AB=( "writing" ) OR AB=( "language" ) OR AB=( "impuls*" ) OR AB=( "hyperact*" ) OR AB=( "ADHD" ) OR AB=( "emoti*" ) OR AB=( " internalizing symptoms" ) OR AB=( " externalizing symptoms" ) OR AB=( " internalizing problems" ) OR AB=( " externalizing problems" ) OR AB=( " anxiety" ) OR AB=( " depression" ) OR AB=( "aggression" ) OR AB=( " delinquency" ) OR AB=( "peer" ) OR AB=( "interpersonal" ) OR AB=( "empathy" ) OR AB=( "autis*" ) OR AB=( "ASD" ) OR AB=( " visual-spatial") OR AB=( "visual-motor" ) OR AB=( " visual spatial") OR AB=( "visual motor" ) ) NOT (AB=( "Animals" ) OR AB=( "mice " ) OR AB=( "mouse" ) OR AB=( "rat" ) OR AB=( "rats" ) OR AB=( "rodent" ) OR AB=( "dog" ) OR AB=( "dogs" ) OR AB=( "pig" ) OR AB=( "pigs" ) OR AB=( "piglet" ) OR AB=( "swine" ) OR AB=( "porcine" ) OR AB=( "animal" ) OR AB=( "fly" ) OR AB=( "Invertebrates" ) OR AB=( "rodentia" ) OR AB=( "models, animal" ) OR AB=( "Animal Experimentation" ) OR AB=( "editorial" ) OR AB=( "commentary" ) OR AB=( "conference abstract" ) OR AB=( "retraction of publication" ) OR AB=( "retracted publication" ) OR TI= ( " systematic revie*" ) OR TI= ( " meta-analy*" ) OR TI= ( " protoco*" ) OR AB=( "case report" ) OR AB=( "case series" ) ) |
| **PubMed** |
| ("neurofibromatosis 1" [Title/Abstract] OR "neurofibromatosis1” [Title/Abstract] OR "NF1” [Title/Abstract] OR "NF 1” [Title/Abstract] OR "neurofibromatosis type 1” [Title/Abstract] OR "neurofibromatosis type1” [Title/Abstract])  AND  ( "cogni*” [Title/Abstract] OR "academi*” [Title/Abstract] OR "socia*” [Title/Abstract] OR "psycho*” [Title/Abstract] OR "behavi*” [Title/Abstract] OR "intelle*” [Title/Abstract] OR "IQ” [Title/Abstract] OR "executive function” [Title/Abstract] OR "executive functioning” [Title/Abstract] OR "attention” [Title/Abstract] OR "inattention” [Title/Abstract] OR "memory” [Title/Abstract] OR "inhibitory control” [Title/Abstract] OR "cognitive flexibility” [Title/Abstract] OR "learning” [Title/Abstract] OR "math” [Title/Abstract] OR "reading” [Title/Abstract] OR "writing” [Title/Abstract] OR "language” [Title/Abstract] OR "impuls*” [Title/Abstract] OR "hyperact*” [Title/Abstract] OR "ADHD” [Title/Abstract] OR "emoti*” [Title/Abstract] OR " internalizing symptoms” [Title/Abstract] OR " externalizing symptoms” [Title/Abstract] OR " internalizing problems” [Title/Abstract] OR " externalizing problems” [Title/Abstract] OR " anxiety” [Title/Abstract] OR " depression” [Title/Abstract] OR "aggression” [Title/Abstract] OR " delinquency” [Title/Abstract] OR "peer” [Title/Abstract] OR "interpersonal” [Title/Abstract] OR "empathy” [Title/Abstract] OR "autis*” [Title/Abstract] OR "ASD” [Title/Abstract] OR " visual-spatial" [Title/Abstract] OR "visual-motor” [Title/Abstract] OR " visual spatial" [Title/Abstract] OR "visual motor” [Title/Abstract] )  NOT  ( "Animals” [Title/Abstract] OR "mice “ [Title/Abstract] OR "mouse” [Title/Abstract] OR "rat” [Title/Abstract] OR "rats” [Title/Abstract] OR "rodent” [Title/Abstract] OR "dog” [Title/Abstract] OR "dogs” [Title/Abstract] OR "pig” [Title/Abstract] OR "pigs” [Title/Abstract] OR "piglet” [Title/Abstract] OR "swine” [Title/Abstract] OR "porcine” [Title/Abstract] OR "animal” [Title/Abstract] OR "fly” [Title/Abstract] OR "Invertebrates” [Title/Abstract] OR "rodentia” [Title/Abstract] OR "models, animal” [Title/Abstract] OR "Animal Experimentation” [Title/Abstract] OR "editorial” [Title/Abstract] OR "commentary” [Title/Abstract] OR "conference abstract” [Title/Abstract] OR "retraction of publication” [Title/Abstract] OR "retracted publication” [Title/Abstract] OR " systematic revie*” [Title] OR " meta-analy*” [Title] OR " protoco*” [Title] OR "case report” [Title/Abstract] OR "case series” [Title/Abstract] ) |
| **ProQuest Dissertations and Theses Global** |
| ( AB( "neurofibromatosis 1" ) OR AB( "neurofibromatosis1" ) OR AB( "NF1" ) OR AB( "NF 1" ) OR AB( "neurofibromatosis type 1" ) OR AB( "neurofibromatosis type1" )) AND ( AB( "cogni*" ) OR AB( "academi*" ) OR AB( "socia*" ) OR AB( "psycho*" ) OR AB( "behavi*" ) OR AB( "intelle*" ) OR AB( "IQ" ) OR AB( "executive function" ) OR AB( "executive functioning" ) OR AB( "attention" ) OR AB( "inattention" ) OR AB( "memory" ) OR AB( "inhibitory control" ) OR AB( "cognitive flexibility" ) OR AB( "learning" ) OR AB( "math" ) OR AB( "reading" ) OR AB( "writing" ) OR AB( "language" ) OR AB( "impuls*" ) OR AB( "hyperact*" ) OR AB( "ADHD" ) OR AB( "emoti*" ) OR AB( " internalizing symptoms" ) OR AB( " externalizing symptoms" ) OR AB( " internalizing problems" ) OR AB( " externalizing problems" ) OR AB( " anxiety" ) OR AB( " depression" ) OR AB( "aggression" ) OR AB( " delinquency" ) OR AB( "peer" ) OR AB( "interpersonal" ) OR AB( "empathy" ) OR AB( "autis*" ) OR AB( "ASD" ) OR AB( " visual-spatial") OR AB( "visual-motor" ) OR AB( " visual spatial") OR AB( "visual motor" ) ) NOT ( AB( "Animals" ) OR AB( "mice " ) OR AB( "mouse" ) OR AB( "rat" ) OR AB( "rats" ) OR AB( "rodent" ) OR AB( "dog" ) OR AB( "dogs" ) OR AB( "pig" ) OR AB( "pigs" ) OR AB( "piglet" ) OR AB( "swine" ) OR AB( "porcine" ) OR AB( "animal" ) OR AB( "fly" ) OR AB( "Invertebrates" ) OR AB( "rodentia" ) OR AB( "models, animal" ) OR AB( "Animal Experimentation" ) OR AB( "editorial" ) OR AB( "commentary" ) OR AB( "conference abstract" ) OR AB( "retraction of publication" ) OR AB( "retracted publication" ) OR ti( " systematic revie*" ) OR ti( " meta-analy*" ) OR ti( " protoco*" ) OR AB( "case report" ) OR AB( "case series" ) ) |

*Note*. Search strategies were developed by Dr. Yang Hou and a doctoral student (both have published meta-analysis studies before), consulting a research librarian. No time limit was applied regarding when studies were published or conducted.

**Online Resource 4**. Inclusion and Exclusion Criteria for the Larger NF1 Neurobehavioral Project

| **Inclusion criteria** |
| --- |
| 1. This is a quantitative study with empirical data (note: medical record data also count); 2. Participants included individuals with NF1(NF1 terms: "neurofibromatosis 1", "neurofibromatosis1", "NF1", "NF 1", “neurofibromatosis type 1”, “neurofibromatosis type1”, “von Recklinghausen's disease”); 3. ^a^Assessed any neurobehavioral functions in individuals with NF1. The project focused on several domains of neurobehavioral functioning: 1) Academic functioning (relevant search terms: academic*, learning, math, writing, reading, language; 2) Socioemotional functioning (relevant search terms: socia*, psycho*, behavi*, emoti*, psycho*, Empathy, peer, interpersonal, autis*, ASD, Internalizing symptoms, Internalizing problems, anxiety, depression, externalizing symptoms, externalizing problems, aggression, delinquency); 3) ADHD (relevant search terms: attention, inattention, hyperact*, impuls*, ADHD); 4) IQ (relevant search terms: intelle*, IQ, Cogni*; 5) Executive functioning (relevant search terms: executive function, executive functioning, attention, inattention, memory, inhibitory control, cognitive flexibility); (6) visual-spatial and visual-motor skills (relevant search terms: visual-spatial, visual-motor, visual spatial, visual motor); 4. ^b^Reported in English. |
| **Exclusion criteria** |
| 1. Not a quantitative study [review, meta-analysis, proposal; qualitative study (e.g., case study, narrative), editorial, commentary, retracted publication etc.]; 2. Did not include NF1 human participants; 3. No neurobehavioral functioning data; 4. Full text was not available in English. |

*Note*. ^a^We planned to write multiple meta-analytic and qualitative review papers on several domains of neurobehavioral functioning (e.g., ADHD, socioemotional functioning, academic functioning, and visual-spatial and visual motor abilities). Thus, the search terms were broad and included terms relevant to all these domains. When screening the papers, we first identified papers that met the criteria in Online Resource 4. Among these neurobehavioral functioning papers, we further identified papers on each specific neurobehavioral domain (internalizing and externalizing problems for the current paper, Online Resource 5). This search and screening strategy can identify more relevant studies than a search focusing on one particular neurobehavioral domain. This is because, in this field, many studies may primarily focus on one neurobehavioral domain but also report data on other neurobehavioral domains. If we only included internalizing/externalizing-related search terms, we may miss studies that did not focus primarily on internalizing/externalizing but included related measures in their study. ^b^We only included studies reported in English because research on the neurobehavioral functioning of individuals with NF1 is mostly reported in English and research showed that excluding non-English publications from syntheses did not change the conclusions of meta-analytical studies (Nussbaumer-Streit et al., 2020).

**Online Resource 5**. Inclusion and Exclusion Criteria for the NF1 Internalizing/Externalizing Study

| **Inclusion criteria** |
| --- |
| 1. Assessed internalizing/externalizing symptoms of individuals with NF1. 2. Included a non-NF1 and non-clinical control group or reported standardized scores of internalizing/externalizing symptoms for individuals with NF1.   **Exclusion criteria**   1. ^a^Papers that examined internalizing or externalizing symptoms in the NF1 group but did not provide sufficient data to calculate the effect sizes in the paper or through contacting authors. 2. ^b^Papers that included a sample used in another study. |
| *Note*. ^a^Thirty-eight papers were excluded based on this criterion (Acosta et al., 2011; Allen et al., 2018; Bottesi et al., 2020; Bulgheroni et al., 2019; J. S. Cohen et al., 2015; R. Cohen et al., 2015; Crook et al., 2022; de Blank et al., 2020; Doser et al., 2020; Fishbein et al., 2022; Garg, 2015; Garg et al., 2013; Garwood et al., 2012; Hamoy-Jimenez et al., 2022; Heimgärtner et al., 2019; Isenberg et al., 2013; Johnson et al., 2005; Johnson et al., 1999; Kenborg et al., 2021; Lalancette et al., 2023; Leidger et al., 2022; Long, 2001; Martin et al., 2021; Martin et al., 2016; Mautner et al., 2015; Merchant et al., 2009; Morris et al., 2021; North et al., 1995; Samuelsson & Riccardi, 1989; Varnhagen et al., 1988; Vranceanu et al., 2014; Walsh et al., 2013; Wang et al., 2012; Wiener et al., 2018; Yamauchi & Suka, 2023; Yoo et al., 2023; Yund, 2020; Zimerman et al., 2015). ^b^Ten papers were excluded based on this criterion (Barton & North, 2007; Chisholm et al., 2023; Coutinho et al., 2017; Huijbregts et al., 2010; Lester et al., 2023; Lewis et al., 2017; Mace et al., 2021; Pierpont et al., 2018; van der Vaart et al., 2013; Wang et al., 2019). |

**Online Resource 6**. Characteristics of Studies Included in the Meta-Analysis

| **Study Label**^a^ | **NF1 *N*** | **Country** | **Mean Age (range)** | **Female %** | **White %** | **Familial NF1 %** | **ADHD %** | **ASD %** | **Mean FSIQ** | **Mean VIQ** | **Mean PIQ** |
| --- | --- | --- | --- | --- | --- | --- | --- | --- | --- | --- | --- |
| Allen 2016 | 23 | United States | 12.1 (8-16) | 34.8 | 73.9 |  |  |  |  |  |  |
| Barton 2004 | 79 | Australia | 11.5 (8-16) | 47 |  | 37 | 39 |  | 90.76 | 92.04 | 91.27 |
| Bawden 1996 | 17 | Canada | 11.3 (7-16) | 47 |  |  | 17.6 |  | 92.8 | 91.9 | 95.8 |
| Berardelli 2021 | 60 | Italy | 43 (18+) | 60 |  |  |  |  |  |  |  |
| Biotteau 2020 (Sporadic) | 55 | France | 9.8 (8-12) | 53 |  | 0 | 0 | 0 | 93.1 | 100.1 | 92.1 |
| Biotteau 2020 (Familial) | 41 | France | 10.2 (8-12) | 59 |  | 100 | 0 | 0 | 85 | 94.5 | 85.2 |
| Buono 2021 | 105 | United States | 43.6 (18-56) | 66 |  |  |  |  |  |  |  |
| Chisholm 2022 | 68 | Australia | 9 (3.4-15.9) | 44 |  | 41 |  | 100 | 84.6 | 88.1 | 87.8 |
| Cipolletta 2018 | 60 | Italy | 11.27 (6-17) | 48 |  | 20 |  |  |  |  |  |
| Coutinho 2016 | 78 | France | 10.42 (5.3-18.9) | 42 |  | 37 | 28 |  | 84.01 | 94.36 | 82.38 |
| Descheemaeker 2005 | 17 | Belgium | 9.2 (7-11) | 29 |  |  |  |  | 90.3 | 92.1 | 88.5 |
| Dilts 1996 | 19 | United States | (6.17-16.92) | 67 | 90 | 45 |  |  |  |  |  |
| Eby 2019 | 104 | United States | 10.33 (3.42-17.5) | 46 |  | 48 | 62 |  | 91.5 |  |  |
| Ferner 1996 (Adults | 61 | United Kingdom | (17-75) |  |  |  |  |  |  |  |  |
| Ferner 1996 (Children) | 22 | United Kingdom | (6-16) |  |  |  |  |  |  |  |  |
| Foy 2022 | 71 | United States; Australia; Canada; Other | 9.8 (3-17.7) | 49 | 92 |  | 58 | 23 |  |  |  |
| Galasso 2014 | 18 | Italy | 11 | 61 |  |  | 0 |  | 101.61 |  |  |
| Gilboa 2011 | 29 | Israel | 12.2 (8-16) | 69 |  |  | 17 |  | 98.9 | 85.9 | 111.9 |
| Graf 2006 | 46 | Switzerland | 11.6 (7.1-16) | 28 |  | 39.1 |  |  |  |  |  |
| Gray 2015^b^ | 56 | United States | 12.3 (6-18) | 37 | 79 | 44 | 36 |  |  |  |  |
| Hardy 2021 | 31 | United States | 10.97 (8-15) | 48 | 90 |  |  |  | 98.1 |  |  |
| Hellebrekers 2022 | 38 | Netherlands | 9.7 (6-16) | 0 |  |  | 52.1 | 10.5 | 91.5 | 93.9 | 89.9 |
| Hou 2022 | 88 | United States | 12.05 (6-18) | 43 | 76 | 47 | 33 |  |  |  |  |
| Huijbregts 2011 | 30 | Netherlands | 11.7 (6.9-17.4) | 60 |  | 47 |  |  |  |  |  |
| Isenberg 2013 | 55 | United States | 9.71 (6-14) |  | 78 |  | 42 |  | 93 | 102 | 84 |
| Klein-Tasman 2014 | 40 | United States | 4.5 (3-6) | 35 | 68 | 40 | 30 |  | 92.55 | 95.23 | 92.75 |
| Lai 2019 | 140 | United States | 12.53 (8-17) | 35.71 | 64.29 | 44.29 | 11.67 |  |  |  |  |
| Lalancette 2022 | 28 | Canada | 9.39 (4-13) | 53.57 |  |  | 50 |  | 89 | 94.68 | 92.23 |
| Lewis 2016 | 23 | Australia | 10.04 (6.67-13.83) | 65 |  |  | 21.7 | 0 | 87.48 |  |  |
| Loitfelder 2015 | 14 | Netherlands | 12.49 | 43 |  |  |  |  |  |  |  |
| Lorenzo 2011 | 39 | Australia | 2.38 (1.75-2.75) | 44 |  | 38 |  |  |  |  |  |
| Lorenzo 2013 | 43 | Australia | 3.35 | 26 |  | 42 | 77 |  | 96.2 | 100.83 | 92.93 |
| Martin 2012 | 53 | United States | 12.4 (6.3-18.7) | 34 |  |  | 32 |  | 95.82 | 99.45 | 92.19 |
| Mautner 2002 | 46 | Germany | 11.07 | 41 |  | 35 | 43 |  | 89.64 |  |  |
| McCurdy 2019^b^ | 20 | United States | 12.55 (10-15) | 55 | 75 | 20 | 35 | 0 | 96.6 |  |  |
| McNeill 2019 | 39 | United States | 11.95 (8-16) | 49 |  | 44 | 39 | 10 |  |  |  |
| Min 2020 | 65 | Other | 37.13 (18-68) | 57 |  | 48 |  |  |  |  |  |
| Morotti 2021 | 45 | United States | 9.17 (4-12) | 51.1 | 80.2 |  | 23.2 | 71 |  |  |  |
| Noll 2007 | 59 | United States | 10.62 (7.92-15.08) | 41 |  |  | 29 |  | 95.15 |  |  |
| Parmeggiani 2018 | 36 | Italy | 9 (7-11) | 47 |  |  | 23 |  | 104.6 | 112.3 | 103.3 |
| Pasini 2012 | 15 | Italy | 13.4 (9-18) | 67 |  | 13.3 |  |  |  |  |  |
| Payne 2019 | 144 | Australia | 11.6 (8-15) | 42 |  |  |  |  |  |  |  |
| Potter 2006^b^ | 60 | United States | 10.6 (6-17) | 48.3 | 79.6 |  |  |  |  |  |  |
| Pride 2018 | 19 | Australia | 11 (7-16) | 53 |  |  | 32 |  | 89.3 |  |  |
| Pride 2023 | 152 | United States; Australia | 8.1(3-15) | 46 |  |  |  |  | 89.1 |  |  |
| Rietman 2017a | 38 | Netherlands | 7.92 (5-13.5) | 42 |  | 58 |  |  | 85.3 | 88.1 | 85.2 |
| Rietman 2017b | 69 | Netherlands | 9.5 (4-16) | 42 |  | 42 | 55 | 10 | 89 | 92 | 88 |
| Rietman 2018 | 183 | Belgium; Netherlands | 10.8 (6-17) | 45 |  | 41 | 33 |  | 85 | 87 | 86 |
| Sangster 2011 | 26 | Australia | 5.25 (4-5.92) | 35 |  |  |  |  | 91.1 | 92.7 | 91.6 |
| Schrimsher 2003 (Ch. 3: NFI Group)^b^ | 37 | United States | 10.6 (6-16.5) | 57 |  |  | 8 |  | 103 |  |  |
| Sharkey 2021 | 7 | United States | 9.26 (5.23-14.05) | 85.71 | 71.43 |  |  |  |  |  |  |
| Stivaros 2018 | 28 | United Kingdom | 8.1 (4.5-10.5) | 20 |  | 43 |  | 100 |  |  |  |
| Taddei 2019 (NF1) | 26 | Italy | 9.33 (1.88-16.25) | 46.2 |  |  |  |  | 99 | 98 | 101 |
| Taddei 2019 (NF1+OPG) | 26 | Italy | 8.92 (2.54-17.17) | 57.7 |  |  |  |  | 86 | 87 | 89 |
| Taddei 2019 (NF1+CT) | 19 | Italy | 7.54 (2.21-15.42) | 31.6 |  |  |  |  | 84 | 90 | 88 |
| Talaei-Khoei 2017 | 43 | United States | 44.07 (18+) | 54.7 |  |  |  |  |  |  |  |
| Tang 2021 | 31 | China | 30.4 (18-60) | 61 |  |  | 0 |  |  |  |  |
| van der Vaart 2016 | 84 | Netherlands | 11.44 (7.9-16) | 54 |  |  |  |  | 83.3 | 86.1 | 83.4 |
| Vaucheret Paz 2019 | 24 | Argentina | 9.88 (5-16) | 42 |  |  | 58.33 |  | 88.91 | 90.08 | 91.08 |
| Wolters 2015 | 60 | United States | 12.7 (6.3-18.8) | 35 | 78 | 47 |  |  |  |  |  |
| Xue 2021 | 20 | China | 29.5 (19-46) | 60 |  |  | 0 | 0 |  |  |  |
| Yoshida 2022 | 73 | Japan | 44.16 (23-84) | 64.4 |  |  |  |  |  |  |  |
| Zöller 1999 | 37 | Sweden | 46.4 (32-65) | 43 |  |  |  |  |  |  |  |

*Note*. ^a^Study labels are composed of first author’s last name and year of publication; for studies that had subgroups of NF1 participants and in which only subgroup data were used in analysis, study labels also include the NF1 subgroup name as labeled in each study. ^b^These studies are unpublished dissertations; all other studies are published journal articles. NF1 *N* = sample size of the included NF1 group. Female % = percentage of females in the NF1 group. White % = percentage of White/Caucasian participants in the NF1 group. Familial NF1 % = percentage of participants with familial NF1. ADHD % = percentage of NF1 participants with a diagnosis or clinically significant levels of attention-deficit/hyperactivity disorder. ASD % = percentage of NF1 participants with a diagnosis or clinically significant levels of autism spectrum disorder. FSIQ = full-scale intelligence quotient. VIQ = verbal intelligence quotient. PIQ = performance intelligence quotient.

**Online Resource 7**. Characteristics of Studies Included in the Meta-Analysis of Depressive Symptoms

| **Study Labe**l^a^ | **Variable Name** | **Measure** | **Informant** | **Control Group Type** | **NF1 *N*** | **Control *N*** | **Hedges’ *g*** | ***LL*** | ***UL*** |
| --- | --- | --- | --- | --- | --- | --- | --- | --- | --- |
| Berardelli 2021 | BDI | Beck Depression Inventory | self-report | healthy community | 60 | 50 | 0.78 | 0.39 | 1.17 |
| Buono 2021 | Depression | Patient Health Questionnaire | self-report | healthy community | 105 | 108 | 0.92 | 0.64 | 1.21 |
| Ferner 1996 (Adults) | depression score | The Hospital Anxiety and Depression Scale | self-report | healthy community | 61 | 61 | 0.17 | -0.19 | 0.52 |
| Gray 2015^b^ | Depression | Behavior Assessment System for Children | parent report | normative data | 56 | 56 | 0.50 | 0.12 | 0.87 |
| Klein-Tasman 2014 | Depression | Behavior Assessment System for Children | parent report | healthy community | 40 | 37 | 0.17 | -0.27 | 0.62 |
| Lai 2019 | Depressive symptoms | Patient-Reported Outcomes Measurement Information System | self-report | normative data | 140 | 140 | 0.31 | 0.08 | 0.55 |
| Lorenzo 2011 | Depression | Behavior Assessment System for Children | parent report | healthy community | 39 | 42 | -0.12 | -0.55 | 0.32 |
| Martin 2012 | Depression | Behavior Assessment System for Children | teacher report | normative data | 53 | 53 | 0.29 | -0.09 | 0.67 |
| Martin 2012 | Depression | Behavior Assessment System for Children | parent report | normative data | 53 | 53 | 0.51 | 0.13 | 0.90 |
| McNeill 2019 | Depression | Behavior Assessment System for Children | parent report | unaffected siblings | 39 | 32 | 0.62 | 0.14 | 1.10 |
| McNeill 2019 | Depression | Behavior Assessment System for Children | self-report | unaffected siblings | 39 | 32 | 0.33 | -0.14 | 0.80 |
| Min 2020 | DEP | Symptom Checklist-90-Revision | self-report | normative data | 65 | 65 | -0.54 | -0.89 | -0.19 |
| Noll 2007 | Depression (CDI) | Children's Depression Inventory | self-report | healthy community | 59 | 59 | 0.18 | -0.18 | 0.54 |
| Parmeggiani 2018 | affective | Child Behavior Checklist | parent report | normative data | 36 | 36 | 1.11 | 0.62 | 1.61 |
| Pride 2023 | CBCL Affective | Child Behavior Checklist | parent report | healthy community | 152 | 96 | 0.93 | 0.66 | 1.20 |
| Sangster 2011 | Depression | Behavior Assessment System for Children | parent report | healthy community | 26 | 21 | 0.57 | -0.02 | 1.16 |
| Talaei-Khoei 2017 | Depression | Patient-Reported Outcomes Measurement Information System | self-report | normative data | 43 | 43 | 0.17 | -0.25 | 0.59 |
| Tang 2021 | Depression | Beck Depression Inventory | self-report | healthy community | 31 | 34 | 1.10 | 0.57 | 1.62 |
| Wolters 2015 | Depression | Behavior Assessment System for Children | self-report | normative data | 60 | 60 | -0.04 | -0.40 | 0.32 |
| Wolters 2015 | Depression | Behavior Assessment System for Children | parent report | normative data | 60 | 60 | 0.51 | 0.15 | 0.88 |
| Xue 2021 | BDI (Depression) | Beck Depression Inventory | self-report | healthy community | 20 | 20 | 0.49 | -0.14 | 1.12 |

*Note*. ^a^Study labels are mostly composed of first author’s last name and year of publication; for studies that had subgroups of NF1 participants and in which only subgroup data were used in analysis, study labels also include the NF1 subgroup name as labeled in each study. ^b^This study is an unpublished dissertation; all other studies are published journal articles. NF1 *N* = sample size of the included NF1 group. Control *N* = sample size of the control group. *LL* = lower limit of 95% confidence interval; *UL* = upper limit of 95% confidence interval.

**Online Resource 8**. Characteristics of Studies Included in the Meta-Analysis of Anxiety Symptoms

| **Study Labe**l^a^ | **Variable Name** | **Measure** | **Informant** | **Control Group Type** | **NF1 *N*** | **Control *N*** | **Hedges’ *g*** | ***LL*** | ***UL*** |
| --- | --- | --- | --- | --- | --- | --- | --- | --- | --- |
| Buono 2021 | Generalized Anxiety | Generalized Anxiety Disorder Scale-7 | self-report | healthy community | 105 | 108 | 0.57 | 0.29 | 0.84 |
| Chisholm 2022 | CBCL Anxiety Problems | Child Behavior Checklist | parent report | normative data | 68 | 68 | 1.32 | 0.94 | 1.69 |
| Ferner 1996 (Adults) | anxiety score | The Hospital Anxiety and Depression Scale | self-report | healthy community | 61 | 61 | 0.17 | -0.18 | 0.53 |
| Ferner 1996 (Children) | anxiety score | The Spielberger Anxiety Trait Inventory for Children | self-report | healthy community | 22 | 23 | 0.25 | -0.34 | 0.84 |
| Gilboa 2011 | Anxious-shy | The Conners Parent Rating Scales-Revised | parent report | healthy community | 29 | 25 | 0.02 | -0.51 | 0.56 |
| Gray 2015^b^ | Anxiety | Behavior Assessment System for Children | parent report | normative data | 56 | 56 | 0.29 | -0.08 | 0.66 |
| Klein-Tasman 2014 | Anxiety | Behavior Assessment System for Children | parent report | healthy community | 40 | 37 | 0.08 | -0.37 | 0.52 |
| Lai 2019 | Anxiety | Patient-Reported Outcomes Measurement Information System | self-report | normative data | 140 | 140 | 0.29 | 0.05 | 0.52 |
| Lorenzo 2011 | Anxiety | Behavior Assessment System for Children | parent report | healthy community | 39 | 42 | -0.98 | -1.44 | -0.52 |
| Martin 2012 | Anxiety | Behavior Assessment System for Children | teacher report | normative data | 53 | 53 | 0.17 | -0.22 | 0.55 |
| Martin 2012 | Anxiety | Behavior Assessment System for Children | parent report | normative data | 53 | 53 | 0.35 | -0.04 | 0.73 |
| McNeill 2019 | Anxiety | Behavior Assessment System for Children | self-report | unaffected siblings | 39 | 32 | -0.11 | -0.58 | 0.36 |
| McNeill 2019 | Anxiety | Behavior Assessment System for Children | parent report | unaffected siblings | 39 | 32 | 0.00 | -0.47 | 0.47 |
| Min 2020 | PHOB | Symptom Checklist-90-Revision | self-report | normative data | 65 | 65 | -0.42 | -0.77 | -0.08 |
| Min 2020 | ANX | Symptom Checklist-90-Revision | self-report | normative data | 65 | 65 | -0.43 | -0.77 | -0.08 |
| Parmeggiani 2018 | anxiety | Child Behavior Checklist | parent report | normative data | 36 | 36 | 1.14 | 0.64 | 1.64 |
| Pasini 2012 | MASC total | Multidimensional Anxiety Scale for Children | self-report | healthy community | 15 | 15 | 1.23 | 0.45 | 2.02 |
| Pasini 2012 | Anxiety Disorder Index | Multidimensional Anxiety Scale for Children | self-report | healthy community | 15 | 15 | 1.08 | 0.31 | 1.85 |
| Pasini 2012 | Social anxiety | Multidimensional Anxiety Scale for Children | self-report | healthy community | 15 | 15 | 1.14 | 0.36 | 1.91 |
| Pride 2023 | CBCL Anxiety | Child Behavior Checklist | parent report | healthy community | 152 | 96 | 0.60 | 0.34 | 0.86 |
| Sangster 2011 | Anxiety | Behavior Assessment System for Children | parent report | healthy community | 26 | 21 | 0.07 | -0.51 | 0.64 |
| Talaei-Khoei 2017 | Anxiety | Patient-Reported Outcomes Measurement Information System | self-report | normative data | 43 | 43 | 0.17 | -0.26 | 0.59 |
| Wolters 2015 | Anxiety | Behavior Assessment System for Children | parent report | normative data | 60 | 60 | 0.28 | -0.08 | 0.64 |
| Wolters 2015 | Anxiety | Behavior Assessment System for Children | self-report | normative data | 60 | 60 | 0.07 | -0.28 | 0.43 |

*Note*. ^a^Study labels are mostly composed of first author’s last name and year of publication; for studies that had subgroups of NF1 participants and in which only subgroup data were used in analysis, study labels also include the NF1 subgroup name as labeled in each study. ^b^This study is an unpublished dissertation; all other studies are published journal articles. NF1 *N* = sample size of the included NF1 group. Control *N* = sample size of the control group. *LL* = lower limit of 95% confidence interval; *UL* = upper limit of 95% confidence interval.

**Online Resource 9**. Characteristics of Studies Included in the Meta-Analysis of Somatic Symptoms

| **Study Labe**l^a^ | **Variable Name** | **Measure** | **Informant** | **Control Group Type** | **NF1 *N*** | **Control *N*** | **Hedges’ *g*** | ***LL*** | ***UL*** |
| --- | --- | --- | --- | --- | --- | --- | --- | --- | --- |
| Barton 2004 | Somatic Complaints | Child Behavior Checklist | parent report | unaffected siblings | 79 | 46 | 0.65 | 0.27 | 1.02 |
| Barton 2004 | Somatic Complaints | Child Behavior Checklist | teacher report | unaffected siblings | 79 | 46 | 0.59 | 0.22 | 0.96 |
| Bawden 1996 | Somatic Complaints | Child Behavior Checklist | parent report | normative data | 17 | 17 | 1.07 | 0.35 | 1.79 |
| Cipolletta 2018 | Somatic complaints | Child Behavior Checklist | parent report | healthy community | 60 | 60 | -0.14 | -0.50 | 0.22 |
| Descheemaeker 2005 | Somatic complains | Child Behavior Checklist | teacher report | normative data | 17 | 17 | 0.68 | -0.02 | 1.37 |
| Descheemaeker 2005 | Somatic complains | Child Behavior Checklist | parent report | normative data | 17 | 17 | 0.72 | 0.02 | 1.41 |
| Dilts 1996 | Somatic Complaints | Child Behavior Checklist | parent report | unaffected siblings | 19 | 19 | 0.57 | -0.08 | 1.21 |
| Dilts 1996 | Somatic Complaints | Child Behavior Checklist | teacher report | unaffected siblings | 19 | 19 | 0.49 | -0.16 | 1.13 |
| Gilboa 2011 | Psychosomatic | The Conners Parent Rating Scales-Revised | parent report | healthy community | 29 | 25 | 0.39 | -0.15 | 0.93 |
| Gray 2015^b^ | Somaticizing | Behavior Assessment System for Children | parent report | normative data | 56 | 56 | 0.60 | 0.22 | 0.98 |
| Klein-Tasman 2014 | Somatic complaints | Behavior Assessment System for Children | parent report | healthy community | 40 | 37 | 0.47 | 0.01 | 0.92 |
| Loitfelder 2015 | Somatic complains | Child Behavior Checklist | parent report | healthy community | 14 | 30 | 1.19 | 0.51 | 1.87 |
| Lorenzo 2011 | Somatic complaints | Behavior Assessment System for Children | parent report | healthy community | 39 | 42 | -0.35 | -0.79 | 0.09 |
| Martin 2012 | Somatic complaints | Behavior Assessment System for Children | teacher report | normative data | 53 | 53 | 0.56 | 0.18 | 0.95 |
| Martin 2012 | Somatic complaints | Behavior Assessment System for Children | parent report | normative data | 53 | 53 | 0.65 | 0.26 | 1.05 |
| Mautner 2002 | Somatic complaints | Child Behavior Checklist | parent report | normative data | 46 | 46 | 0.68 | 0.26 | 1.10 |
| Mautner 2002 | Somatic complaints | Child Behavior Checklist | teacher report | normative data | 46 | 46 | 0.34 | -0.07 | 0.76 |
| McNeill 2019 | Somatic complaints | Behavior Assessment System for Children | parent report | unaffected siblings | 39 | 32 | 0.50 | 0.02 | 0.97 |
| Min 2020 | SOM | Symptom Checklist-90-Revision | self-report | normative data | 65 | 65 | -0.48 | -0.82 | -0.13 |
| Noll 2007 | Somatic complaints | Child Behavior Checklist | parent report | healthy community | 59 | 59 | 0.30 | -0.07 | 0.66 |
| Noll 2007 | Somatic complaints | Child Behavior Checklist | parent report | healthy community | 59 | 59 | 0.27 | -0.10 | 0.63 |
| Parmeggiani 2018 | somatic problems | Child Behavior Checklist | parent report | normative data | 36 | 36 | 0.97 | 0.48 | 1.45 |
| Rietman 2018 | Somatic complaints | Child Behavior Checklist | self-report | normative data | 183 | 183 | 0.86 | 0.64 | 1.07 |
| Rietman 2018 | Somatic complaints | Child Behavior Checklist | teacher report | normative data | 183 | 183 | 0.67 | 0.46 | 0.88 |
| Rietman 2018 | Somatic complaints | Child Behavior Checklist | parent report | normative data | 183 | 183 | 1.00 | 0.78 | 1.22 |
| Sangster 2011 | Somatic complaints | Behavior Assessment System for Children | parent report | healthy community | 26 | 21 | 1.92 | 1.22 | 2.62 |
| Vaucheret Paz 2019 | Somatic complaints | Child Behavior Checklist | parent report | normative data | 24 | 24 | 1.27 | 0.65 | 1.89 |

*Note*. ^a^Study labels are mostly composed of first author’s last name and year of publication; for studies that had subgroups of NF1 participants and in which only subgroup data were used in analysis, study labels also include the NF1 subgroup name as labeled in each study. ^b^This study is an unpublished dissertation; all other studies are published journal articles. NF1 *N* = sample size of the included NF1 group. Control *N* = sample size of the control group. *LL* = lower limit of 95% confidence interval; *UL* = upper limit of 95% confidence interval.

**Online Resource 10.** Characteristics of Studies Included in the Meta-Analysis of Total Internalizing Symptoms

| **Study Label**^a^ | **Variable Name** | **Measure** | **Informant** | **Control Group Type** | **NF1 *N*** | **Control *N*** | **Hedges’ *g*** | ***LL*** | ***UL*** |  |
| --- | --- | --- | --- | --- | --- | --- | --- | --- | --- | --- |
| Allen 2016 | Internalizing | Child Behavior Checklist | parent report | healthy community | 23 | 23 | 1.09 | 0.47 | 1.71 |  |
| Barton 2004 | Internalizing | Child Behavior Checklist | teacher report | unaffected siblings | 79 | 46 | 0.58 | 0.21 | 0.95 |  |
| Barton 2004 | Anxious/Depressed | Child Behavior Checklist | teacher report | unaffected siblings | 79 | 46 | 0.48 | 0.11 | 0.85 |  |
| Barton 2004 | Internalizing | Child Behavior Checklist | parent report | unaffected siblings | 79 | 46 | 0.29 | -0.08 | 0.65 |  |
| Barton 2004 | Anxious/Depressed | Child Behavior Checklist | parent report | unaffected siblings | 79 | 46 | 0.10 | -0.26 | 0.46 |  |
| Bawden 1996 | Anxiety-Depression | Child Behavior Checklist | parent report | normative data | 17 | 17 | 0.94 | 0.23 | 1.65 |  |
| Bawden 1996 | Internalizing Behavior Problems | Child Behavior Checklist | parent report | normative data | 17 | 17 | 0.74 | 0.04 | 1.44 |  |
| Biotteau 2020 (Sporadic) | Internalizing | Child Behavior Checklist | parent report | normative data | 55 | 55 | -1.01 | -1.40 | -0.61 |  |
| Biotteau 2020 (Sporadic) | Anxiety/Depression | Child Behavior Checklist | parent report | normative data | 55 | 55 | -0.90 | -1.29 | -0.51 |  |
| Biotteau 2020 (Familial) | Internalizing | Child Behavior Checklist | parent report | normative data | 41 | 41 | -0.93 | -1.39 | -0.48 |  |
| Biotteau 2020 (Familial) | Anxiety/Depression | Child Behavior Checklist | parent report | normative data | 41 | 41 | -0.83 | -1.28 | -0.38 |  |
| Cipolletta 2018 | TOT internalizations | Child Behavior Checklist | parent report | healthy community | 60 | 60 | 0.16 | -0.20 | 0.52 |  |
| Cipolletta 2018 | Anxious/depressed | Child Behavior Checklist | parent report | healthy community | 60 | 60 | 0.23 | -0.13 | 0.59 |  |
| Coutinho 2016 | Internalizing | Child Behavior Checklist | parent report | normative data | 78 | 78 | 0.77 | 0.44 | 1.09 |  |
| Descheemaeker 2005 | Anxious/depressed | Child Behavior Checklist | parent report | normative data | 17 | 17 | 0.63 | -0.06 | 1.31 |  |
| Descheemaeker 2005 | Internalizing | Child Behavior Checklist | parent report | normative data | 17 | 17 | 0.09 | -0.58 | 0.77 |  |
| Descheemaeker 2005 | Internalizing | Child Behavior Checklist | teacher report | normative data | 17 | 17 | 0.67 | -0.02 | 1.36 |  |
| Dilts 1996 | Internalizing factor | Child Behavior Checklist | parent report | unaffected siblings | 19 | 19 | 0.90 | 0.23 | 1.57 |  |
| Dilts 1996 | Anxious/Depressed | Child Behavior Checklist | teacher report | unaffected siblings | 19 | 19 | 0.68 | 0.03 | 1.34 |  |
| Dilts 1996 | Anxious/Depressed | Child Behavior Checklist | parent report | unaffected siblings | 19 | 19 | 0.78 | 0.11 | 1.44 |  |
| Dilts 1996 | Internalizing factor | Child Behavior Checklist | teacher report | unaffected siblings | 19 | 19 | 0.45 | -0.19 | 1.10 |  |
| Eby 2019 | Internalizing Problems | Behavior Assessment System for Children | parent report | normative data | 104 | 104 | 0.56 | 0.28 | 0.83 |  |
| Foy 2022 | Emotional symptoms | Strengths and Difficulties Questionnaire (SDQ) | parent report | normative data | 71 | 71 | 1.04 | 0.69 | 1.39 |  |
| Graf 2006 | Internalizing | Child Behavior Checklist | parent report | normative data | 46 | 46 | 0.83 | 0.40 | 1.25 |  |
| Gray 2015^b^ | Internalizing | Behavior Assessment System for Children | parent report | normative data | 56 | 56 | 0.57 | 0.19 | 0.95 |  |
| Hardy 2021 | Internalizing T-Score | Child Behavior Checklist | parent report | normative data | 31 | 31 | 0.61 | 0.10 | 1.12 |  |
| Hellebrekers 2022 | Anxiety/Depression | Child Behavior Checklist | parent report | normative data | 38 | 38 | 0.56 | 0.10 | 1.02 |  |
| Hellebrekers 2022 | Intern. Prob. | Child Behavior Checklist | teacher report | normative data | 38 | 38 | 0.97 | 0.49 | 1.44 |  |
| Hellebrekers 2022 | Anxiety/Depression | Child Behavior Checklist | teacher report | normative data | 38 | 38 | 0.93 | 0.45 | 1.40 |  |
| Hellebrekers 2022 | Intern. Prob. | Child Behavior Checklist | parent report | normative data | 38 | 38 | 0.52 | 0.06 | 0.98 |  |
| Hou 2022 | Internalizing Problems | Behavior Assessment System for Children | self-report | normative data | 88 | 88 | -0.01 | -0.31 | 0.28 |  |
| Hou 2022 | Internalizing Problems | Behavior Assessment System for Children | parent report | normative data | 88 | 88 | 0.61 | 0.31 | 0.92 |  |
| Klein-Tasman 2014 | Internalizing Problems | Behavior Assessment System for Children | parent report | healthy community | 40 | 37 | 0.37 | -0.08 | 0.83 |  |
| Lalancette 2022 | internalizing | Child Behavior Checklist | parent report | healthy community | 28 | 28 | 0.65 | 0.11 | 1.19 |  |
| Loitfelder 2015 | Anxious/depressed | Child Behavior Checklist | parent report | healthy community | 14 | 30 | 0.62 | -0.03 | 1.26 |  |
| Loitfelder 2015 | Withdrawn/depressed | Child Behavior Checklist | parent report | healthy community | 14 | 30 | 2.10 | 1.33 | 2.88 |  |
| Lorenzo 2011 | Internalizing Problems | Behavior Assessment System for Children | parent report | healthy community | 39 | 42 | -0.48 | -0.92 | -0.04 |  |
| Lorenzo 2013 | Internalizing | Behavior Assessment System for Children | parent report | healthy community | 43 | 43 | -0.47 | -0.90 | -0.04 |  |
| Martin 2012 | Internalizing Problems | Behavior Assessment System for Children | parent report | normative data | 53 | 53 | 0.62 | 0.23 | 1.01 |  |
| Martin 2012 | Internalizing Problems | Behavior Assessment System for Children | teacher report | normative data | 53 | 53 | 0.36 | -0.02 | 0.74 |  |
| Mautner 2002 | Internalizing | Child Behavior Checklist | teacher report | normative data | 46 | 46 | 0.02 | -0.39 | 0.43 |  |
| Mautner 2002 | Anxiety/Depression | Child Behavior Checklist | parent report | normative data | 46 | 46 | 1.04 | 0.61 | 1.48 |  |
| Mautner 2002 | Internalizing | Child Behavior Checklist | parent report | normative data | 46 | 46 | 0.87 | 0.44 | 1.30 |  |
| Mautner 2002 | Anxiety/Depression | Child Behavior Checklist | teacher report | normative data | 46 | 46 | 0.53 | 0.11 | 0.95 |  |
| Morotti 2021 | Internalizing | Child Behavior Checklist | parent report | healthy community | 45 | 180 | 0.78 | 0.44 | 1.11 |  |
| Noll 2007 | Anxious-depressed | Child Behavior Checklist | parent report | healthy community | 59 | 59 | 0.36 | 0.00 | 0.73 |  |
| Noll 2007 | Anxious-depressed | Child Behavior Checklist | parent report | healthy community | 59 | 59 | 0.22 | -0.14 | 0.58 |  |
| Noll 2007 | Internalizing symptoms | Child Behavior Checklist | parent report | healthy community | 59 | 59 | 0.25 | -0.11 | 0.62 |  |
| Noll 2007 | Internalizing symptoms | Child Behavior Checklist | parent report | healthy community | 59 | 59 | 0.49 | 0.12 | 0.85 |  |
| Payne 2019 | Internalizing problems | Behavior Assessment System for Children | self-report | normative data | 144 | 144 | -0.10 | -0.33 | 0.13 |  |
| Payne 2019 | Internalizing problems | Behavior Assessment System for Children | parent report | normative data | 144 | 144 | -0.36 | -0.60 | -0.13 |  |
| Potter 2006^c^ | Internalizing Problems | Behavior Assessment System for Children | parent report | normative data | 60 | 60 | 0.57 | 0.20 | 0.93 |  |
| Rietman 2017a | Internalizing | Child Behavior Checklist | parent report | normative data | 38 | 38 | 1.01 | 0.53 | 1.49 |  |
| Rietman 2017a | Internalizing | Child Behavior Checklist | teacher report | normative data | 38 | 38 | 0.71 | 0.25 | 1.18 |  |
| Rietman 2017b | Internalizing problems | Child Behavior Checklist | parent report | normative data | 69 | 69 | 0.90 | 0.55 | 1.25 |  |
| Rietman 2018 | Anxious-depressed | Child Behavior Checklist | parent report | normative data | 183 | 183 | 0.67 | 0.46 | 0.88 |  |
| Rietman 2018 | Anxious-depressed | Child Behavior Checklist | self-report | normative data | 183 | 183 | 0.67 | 0.46 | 0.88 |  |
| Rietman 2018 | Anxious-depressed | Child Behavior Checklist | teacher report | normative data | 183 | 183 | 0.84 | 0.62 | 1.05 |  |
| Rietman 2018 | Internalizing problems | Child Behavior Checklist | parent report | normative data | 183 | 183 | 0.64 | 0.43 | 0.85 |  |
| Rietman 2018 | Internalizing problems | Child Behavior Checklist | self-report | normative data | 183 | 183 | 0.43 | 0.23 | 0.64 |  |
| Rietman 2018 | Internalizing problems | Child Behavior Checklist | teacher report | normative data | 183 | 183 | 0.60 | 0.39 | 0.81 |  |
| Rietman 2018 | Withdrawn-depressed | Child Behavior Checklist | parent report | normative data | 183 | 183 | 0.86 | 0.65 | 1.08 |  |
| Rietman 2018 | Withdrawn-depressed | Child Behavior Checklist | self-report | normative data | 183 | 183 | 0.73 | 0.51 | 0.94 |  |
| Rietman 2018 | Anxious-depressed | Child Behavior Checklist | parent report | normative data | 183 | 183 | 0.80 | 0.59 | 1.02 |  |
| Sangster 2011 | Internalizing Problems Composite | Behavior Assessment System for Children | parent report | healthy community | 26 | 21 | 0.88 | 0.27 | 1.48 |  |
| Schrimsher 2003 (Ch. 3: NFI Group)^b^ | Internalizing Problems | Child Behavior Checklist | parent report | normative data | 37 | 37 | 0.20 | -0.25 | 0.66 |  |
| Sharkey 2021 | Internalizing problems | Child Behavior Checklist | parent report | normative data | 7 | 7 | 1.34 | 0.16 | 2.51 |  |
| Sharkey 2021 | Internalizing problems | Child Behavior Checklist | teacher report | normative data | 7 | 7 | 1.28 | 0.11 | 2.44 |  |
| Taddei 2019 (NF1) | Int Total | Child Behavior Checklist | parent report | normative data | 26 | 26 | 0.48 | -0.07 | 1.03 |  |
| Taddei 2019 (NF1+OPG) | Int Total | Child Behavior Checklist | parent report | normative data | 19 | 19 | 0.36 | -0.28 | 1.01 |  |
| Taddei 2019 (NF1+CT) | Int Total | Child Behavior Checklist | parent report | normative data | 26 | 26 | 0.78 | 0.21 | 1.34 |  |
| van der Vaart 2016 | Internalizing behavioral problems | Child Behavior Checklist | parent report | normative data | 84 | 84 | 0.58 | 0.27 | 0.89 |  |
| Vaucheret Paz 2019 | Anxious/depressed | Child Behavior Checklist | parent report | normative data | 24 | 24 | 0.93 | 0.33 | 1.52 |  |
| Vaucheret Paz 2019 | Withdrawn/depressed | Child Behavior Checklist | parent report | normative data | 24 | 24 | 0.66 | 0.08 | 1.24 |  |
| Yoshida 2022 | Anxiety/depression | EQ-5D-5L | self-report | healthy community | 73 | 76 | 1.33 | 0.98 | 1.69 |  |

*Note*.  ^a^Study labels are mostly composed of first author’s last name and year of publication; for studies that had subgroups of NF1 participants and in which only subgroup data were used in analysis, study labels also include the NF1 subgroup name as labeled in each study. ^b^These studies are unpublished dissertations; all other studies are published journal articles. ^c^This study included a typically developing control group but did not provide data for it, and thus normative data were used as the control; this study is also an unpublished dissertation. NF1 *N* = sample size of the included NF1 group. Control *N* = sample size of the control group. *LL* = lower limit of 95% confidence interval; *UL* = upper limit of 95% confidence interval.

**Online Resource 11**. Characteristics of Studies Included in the Meta-Analysis of Aggression

| **Study Labe**l^a^ | **Variable Name** | **Measure** | **Informant** | **Control Group Type** | **NF1 *N*** | **Control *N*** | **Hedges’ *g*** | ***LL*** | ***UL*** |
| --- | --- | --- | --- | --- | --- | --- | --- | --- | --- |
| Barton 2004 | Aggressive Behavior | Child Behavior Checklist | teacher report | unaffected siblings | 79 | 46 | 0.11 | -0.25 | 0.48 |
| Barton 2004 | Aggressive Behavior | Child Behavior Checklist | parent report | unaffected siblings | 79 | 46 | 0.20 | -0.16 | 0.57 |
| Bawden 1996 | Aggressive Behavior | Child Behavior Checklist | parent report | normative data | 17 | 17 | 0.73 | 0.03 | 1.42 |
| Biotteau 2020 (Sporadic) | Aggressive behaviors | Child Behavior Checklist | parent report | normative data | 55 | 55 | 0.00 | -0.37 | 0.37 |
| Biotteau 2020 (Familial) | Aggressive behaviors | Child Behavior Checklist | parent report | normative data | 41 | 41 | -0.29 | -0.72 | 0.15 |
| Cipolletta 2018 | Aggressiveness | Child Behavior Checklist | parent report | healthy community | 60 | 60 | 0.20 | -0.16 | 0.56 |
| Descheemaeker 2005 | Aggressive behaviour | Child Behavior Checklist | parent report | normative data | 17 | 17 | 0.60 | -0.09 | 1.29 |
| Descheemaeker 2005 | Aggressive behaviour | Child Behavior Checklist | teacher report | normative data | 17 | 17 | 0.70 | 0.01 | 1.39 |
| Dilts 1996 | Aggressive Behavior | Child Behavior Checklist | teacher report | unaffected siblings | 19 | 19 | 0.51 | -0.14 | 1.15 |
| Dilts 1996 | Aggressive Behavior | Child Behavior Checklist | parent report | unaffected siblings | 19 | 19 | 0.43 | -0.21 | 1.07 |
| Hellebrekers 2022 | Aggression | Child Behavior Checklist | teacher report | normative data | 38 | 38 | 0.92 | 0.45 | 1.39 |
| Hellebrekers 2022 | Aggression | Child Behavior Checklist | parent report | normative data | 38 | 38 | 1.06 | 0.58 | 1.54 |
| Klein-Tasman 2014 | Aggression | Behavior Assessment System for Children | parent report | healthy community | 40 | 37 | -0.16 | -0.61 | 0.29 |
| Loitfelder 2015 | Aggressive behavior | Child Behavior Checklist | parent report | healthy community | 14 | 30 | 1.09 | 0.41 | 1.76 |
| Lorenzo 2011 | Aggression | Behavior Assessment System for Children | parent report | healthy community | 39 | 42 | -0.88 | -1.34 | -0.42 |
| Martin 2012 | Aggression | Behavior Assessment System for Children | parent report | normative data | 53 | 53 | -0.20 | -0.58 | 0.18 |
| Martin 2012 | Aggression | Behavior Assessment System for Children | teacher report | normative data | 53 | 53 | -0.08 | -0.46 | 0.30 |
| Mautner 2002 | Aggressive | Child Behavior Checklist | teacher report | normative data | 46 | 46 | 0.66 | 0.24 | 1.08 |
| Mautner 2002 | Aggressive | Child Behavior Checklist | parent report | normative data | 46 | 46 | 1.08 | 0.64 | 1.52 |
| McCurdy 2019^b^ | Aggression | Behavior Assessment System for Children | parent report | healthy community | 20 | 14 | 0.37 | -0.32 | 1.05 |
| McCurdy 2019^b^ | Aggression | Behavior Assessment System for Children | teacher report | healthy community | 20 | 14 | -0.17 | -0.86 | 0.51 |
| McNeill 2019 | Aggression | Behavior Assessment System for Children | parent report | unaffected siblings | 39 | 32 | 0.64 | 0.16 | 1.12 |
| Noll 2007 | Aggressive behavior | Child Behavior Checklist | parent report | healthy community | 59 | 59 | 0.53 | 0.17 | 0.90 |
| Noll 2007 | Aggressive behavior | Child Behavior Checklist | parent report | healthy community | 59 | 59 | 0.29 | -0.08 | 0.65 |
| Rietman 2018 | Aggressive behavior | Child Behavior Checklist | parent report | normative data | 183 | 183 | 0.83 | 0.61 | 1.04 |
| Rietman 2018 | Aggressive behavior | Child Behavior Checklist | teacher report | normative data | 183 | 183 | 0.65 | 0.44 | 0.86 |
| Rietman 2018 | Aggressive behavior | Child Behavior Checklist | self-report | normative data | 183 | 183 | 0.41 | 0.20 | 0.61 |
| Sangster 2011 | Aggression | Behavior Assessment System for Children | parent report | healthy community | 26 | 21 | 0.06 | -0.51 | 0.64 |
| Stivaros 2018 | Aggression | Conners | parent report | normative data | 28 | 28 | 1.35 | 0.77 | 1.93 |
| Vaucheret Paz 2019 | Aggressive behaviour | Child Behavior Checklist | parent report | normative data | 24 | 24 | 0.85 | 0.26 | 1.45 |
| Zöller 1999 | Aggression | Karolinska Scales of Personality Inventory | self-report | healthy community | 37 | 27 | -0.78 | -1.29 | -0.26 |
| Zöller 1999 | Hostility | Karolinska Scales of Personality Inventory | self-report | healthy community | 37 | 27 | 0.16 | -0.33 | 0.66 |
| Zöller 1999 | Indirect aggression | Karolinska Scales of Personality Inventory | self-report | healthy community | 37 | 27 | -0.69 | -1.20 | -0.18 |

*Note*. ^a^Study labels are mostly composed of first author’s last name and year of publication; for studies that had subgroups of NF1 participants and in which only subgroup data were used in analysis, study labels also include the NF1 subgroup name as labeled in each study. ^b^These studies are unpublished dissertations; all other studies are published journal articles. NF1 *N* = sample size of the included NF1 group. Control *N* = sample size of the control group. *LL* = lower limit of 95% confidence interval; *UL* = upper limit of 95% confidence interval.

**Online Resource 12**. Characteristics of Studies Included in the Meta-Analysis of Delinquency

| **Study Labe**l^a^ | **Variable Name** | **Measure** | **Informant** | **Control Group Type** | **NF1 *N*** | **Control *N*** | **Hedges’ *g*** | ***LL*** | ***UL*** |
| --- | --- | --- | --- | --- | --- | --- | --- | --- | --- |
| Barton 2004 | Delinquent Behavior | Child Behavior Checklist | parent report | unaffected siblings | 79 | 46 | 0.12 | -0.24 | 0.49 |
| Barton 2004 | Delinquent Behavior | Child Behavior Checklist | teacher report | unaffected siblings | 79 | 46 | -0.09 | -0.45 | 0.28 |
| Bawden 1996 | Delinquent Behavior | Child Behavior Checklist | parent report | normative data | 17 | 17 | 0.53 | -0.16 | 1.21 |
| Biotteau 2020 (Sporadic) | Delinquent behaviors | Child Behavior Checklist | parent report | normative data | 55 | 55 | -0.19 | -0.57 | 0.18 |
| Biotteau 2020 (Familial) | Delinquent behaviors | Child Behavior Checklist | parent report | normative data | 41 | 41 | -0.29 | -0.72 | 0.15 |
| Chisholm 2022 | Oppositional Defant Problems | Child Behavior Checklist | parent report | normative data | 68 | 68 | 1.09 | 0.73 | 1.45 |
| Cipolletta 2018 | Delinquency | Child Behavior Checklist | parent report | healthy community | 60 | 60 | 0.33 | -0.03 | 0.69 |
| Descheemaeker 2005 | Delinquent behaviour | Child Behavior Checklist | teacher report | normative data | 17 | 17 | 0.34 | -0.34 | 1.01 |
| Descheemaeker 2005 | Delinquent behaviour | Child Behavior Checklist | parent report | normative data | 17 | 17 | 0.51 | -0.17 | 1.20 |
| Dilts 1996 | Delinquent Behavior | Child Behavior Checklist | parent report | unaffected siblings | 19 | 19 | 0.44 | -0.21 | 1.08 |
| Dilts 1996 | Delinquent Behavior | Child Behavior Checklist | teacher report | unaffected siblings | 19 | 19 | 0.50 | -0.15 | 1.14 |
| Foy 2022 | Conduct Problems | Strengths and Difficulties Questionnaire | parent report | normative data | 71 | 71 | 0.46 | 0.13 | 0.80 |
| Galasso 2014 | Oppositional | Conners ADHD/DSM-IV Scales | parent report | healthy community | 18 | 18 | 1.55 | 0.80 | 2.30 |
| Gilboa 2011 | Oppositional | The Conners Parent Rating Scales-Revised | parent report | healthy community | 29 | 25 | -0.11 | -0.65 | 0.42 |
| Hellebrekers 2022 | Rule-Breaking | Child Behavior Checklist | parent report | normative data | 38 | 38 | 0.64 | 0.17 | 1.10 |
| Hellebrekers 2022 | Rule-Breaking | Child Behavior Checklist | teacher report | normative data | 38 | 38 | 0.61 | 0.15 | 1.07 |
| Huijbregts 2011 | Conduct problems | Strengths and Difficulties Questionnaire | parent report | unaffected siblings | 30 | 30 | 0.65 | 0.13 | 1.17 |
| Isenberg 2013 | Conduct problems | Conners Third Edition-Parent | parent report | normative data | 55 | 55 | 0.12 | -0.25 | 0.50 |
| Isenberg 2013 | Oppositional-defiant | Conners Third Edition-Parent | parent report | normative data | 55 | 55 | 0.38 | 0.00 | 0.75 |
| Loitfelder 2015 | Rule-breaking behavior | Child Behavior Checklist | parent report | healthy community | 14 | 30 | 1.00 | 0.33 | 1.67 |
| Martin 2012 | Conduct Problems | Behavior Assessment System for Children | parent report | normative data | 53 | 53 | -0.01 | -0.39 | 0.37 |
| Martin 2012 | Conduct Problems | Behavior Assessment System for Children | teacher report | normative data | 53 | 53 | -0.24 | -0.62 | 0.14 |
| Mautner 2002 | Delinquent | Child Behavior Checklist | teacher report | normative data | 46 | 46 | 0.56 | 0.14 | 0.97 |
| Mautner 2002 | Delinquent | Child Behavior Checklist | parent report | normative data | 46 | 46 | 0.51 | 0.10 | 0.93 |
| McNeill 2019 | Conduct problems | Behavior Assessment System for Children | parent report | unaffected siblings | 39 | 32 | 0.42 | -0.05 | 0.90 |
| Noll 2007 | Delinquent behavior | Child Behavior Checklist | parent report | healthy community | 59 | 59 | 0.39 | 0.03 | 0.76 |
| Noll 2007 | Delinquent behavior | Child Behavior Checklist | parent report | healthy community | 59 | 59 | 0.07 | -0.29 | 0.43 |
| Parmeggiani 2018 | oppositional/defiant | Child Behavior Checklist | parent report | normative data | 36 | 36 | 0.78 | 0.30 | 1.26 |
| Parmeggiani 2018 | conduct problems | Child Behavior Checklist | parent report | normative data | 36 | 36 | 0.89 | 0.40 | 1.37 |
| Rietman 2018 | Rule-breaking behavior | Child Behavior Checklist | parent report | normative data | 183 | 183 | 0.57 | 0.36 | 0.78 |
| Rietman 2018 | Rule-breaking behavior | Child Behavior Checklist | teacher report | normative data | 183 | 183 | 0.37 | 0.16 | 0.58 |
| Rietman 2018 | Rule-breaking behavior | Child Behavior Checklist | self-report | normative data | 183 | 183 | 0.37 | 0.17 | 0.58 |
| Taddei 2019 (NF1) | Oppositional Deviant Problems | Child Behavior Checklist | parent report | normative data | 26 | 26 | 0.61 | 0.05 | 1.17 |
| Taddei 2019 (NF1+OPG) | Oppositional Deviant Problems | Child Behavior Checklist | parent report | normative data | 26 | 26 | 0.89 | 0.32 | 1.46 |
| Taddei 2019 (NF1+CT) | Oppositional Deviant Problems | Child Behavior Checklist | parent report | normative data | 19 | 19 | 0.23 | -0.41 | 0.87 |
| Vaucheret Paz 2019 | Oppositional | Conners Continuous Performance Test II | performance based test | normative data | 24 | 24 | 0.68 | 0.10 | 1.26 |
| Vaucheret Paz 2019 | Rule-breaking behaviour | Child Behavior Checklist | parent report | normative data | 24 | 24 | 0.71 | 0.12 | 1.29 |

*Note*. ^a^Study labels are mostly composed of first author’s last name and year of publication; for studies that had subgroups of NF1 participants and in which only subgroup data were used in analysis, study labels also include the NF1 subgroup name as labeled in each study. NF1 *N* = sample size of the included NF1 group. Control *N* = sample size of the control group. *LL* = lower limit of 95% confidence interval; *UL* = upper limit of 95% confidence interval. All papers included are published journal articles.

**Online Resource 13**. Characteristics of Studies Included in the Meta-Analysis of Total Externalizing Symptoms

| **Study Labe**l^a^ | **Variable Name** | **Measure** | **Informant** | **Control Group Type** | **NF1 *N*** | **Control *N*** | **Hedges’ *g*** | ***LL*** | ***UL*** |
| --- | --- | --- | --- | --- | --- | --- | --- | --- | --- |
| Allen 2016 | Externalising Problems | Child Behavior Checklist | parent report | healthy community | 23 | 23 | 0.29 | -0.29 | 0.87 |
| Barton 2004 | Externalizing | Child Behavior Checklist | parent report | unaffected siblings | 79 | 46 | 0.14 | -0.23 | 0.50 |
| Barton 2004 | Externalizing | Child Behavior Checklist | teacher report | unaffected siblings | 79 | 46 | 0.07 | -0.29 | 0.43 |
| Bawden 1996 | Externalizing Behavior Problems | Child Behavior Checklist | parent report | normative data | 17 | 17 | 0.16 | -0.52 | 0.83 |
| Biotteau 2020 (Sporadic) | Externalizing | Child Behavior Checklist | parent report | normative data | 55 | 55 | 0.00 | -0.37 | 0.37 |
| Biotteau 2020 (Familial) | Externalizing | Child Behavior Checklist | parent report | normative data | 41 | 41 | -0.27 | -0.71 | 0.16 |
| Cipolletta 2018 | externalization | Child Behavior Checklist | parent report | healthy community | 60 | 60 | 0.11 | -0.25 | 0.47 |
| Coutinho 2016 | Externalizing | Child Behavior Checklist | parent report | normative data | 78 | 78 | 0.45 | 0.14 | 0.77 |
| Descheemaeker 2005 | Externalizing | Child Behavior Checklist | parent report | normative data | 17 | 17 | 0.00 | -0.67 | 0.67 |
| Descheemaeker 2005 | Externalizing | Child Behavior Checklist | teacher report | normative data | 17 | 17 | 0.30 | -0.37 | 0.98 |
| Dilts 1996 | Externalizing factor | Child Behavior Checklist | teacher report | unaffected siblings | 19 | 19 | 0.36 | -0.29 | 1.00 |
| Dilts 1996 | Externalizing factor | Child Behavior Checklist | parent report | unaffected siblings | 19 | 19 | 0.24 | -0.39 | 0.88 |
| Eby 2019 | Externalizing Problems | Behavior Assessment System for Children | parent report | normative data | 104 | 104 | 0.58 | 0.30 | 0.86 |
| Graf 2006 | Externalizing | Child Behavior Checklist | parent report | normative data | 46 | 46 | 0.42 | 0.01 | 0.84 |
| Hardy 2021 | Externalizing T-Score | Child Behavior Checklist | parent report | normative data | 31 | 31 | 0.27 | -0.23 | 0.77 |
| Hellebrekers 2022 | Extern. Prob | Child Behavior Checklist | parent report | normative data | 38 | 38 | 0.54 | 0.08 | 1.00 |
| Hellebrekers 2022 | Extern Prob | Child Behavior Checklist | teacher report | normative data | 38 | 38 | 0.57 | 0.11 | 1.03 |
| Hou 2022 | Externalizing Problems | Behavior Assessment System for Children | parent report | normative data | 88 | 88 | 0.16 | -0.14 | 0.45 |
| Klein-Tasman 2014 | Externalizing Problems | Behavior Assessment System for Children | parent report | healthy community | 40 | 37 | 0.07 | -0.37 | 0.52 |
| Lalancette 2022 | Externalizing | Child Behavior Checklist | parent report | healthy community | 28 | 28 | 0.62 | 0.09 | 1.16 |
| Lewis 2016^b^ | Defiance/Aggression | Conners 3-Parent Long Form | parent report | normative data | 23 | 23 | 0.39 | -0.19 | 0.98 |
| Lorenzo 2011 | Externalizing Problems | Behavior Assessment System for Children | parent report | healthy community | 39 | 42 | -0.84 | -1.29 | -0.38 |
| Lorenzo 2013 | Externalizing | Behavior Assessment System for Children | parent report | healthy community | 43 | 43 | 0.12 | -0.30 | 0.55 |
| Martin 2012 | Externalizing Problems | Behavior Assessment System for Children | teacher report | normative data | 53 | 53 | -0.01 | -0.39 | 0.37 |
| Martin 2012 | Externalizing Problems | Behavior Assessment System for Children | parent report | normative data | 53 | 53 | 0.05 | -0.33 | 0.43 |
| Mautner 2002 | Externalizing | Child Behavior Checklist | parent report | normative data | 46 | 46 | 0.89 | 0.46 | 1.32 |
| Mautner 2002 | Externalizing | Child Behavior Checklist | teacher report | normative data | 46 | 46 | 0.45 | 0.04 | 0.87 |
| Morotti 2021 | Externalizing | Child Behavior Checklist | parent report | healthy community | 45 | 180 | 0.43 | 0.11 | 0.76 |
| Noll 2007 | Externalizing problems | Child Behavior Checklist | parent report | healthy community | 59 | 59 | 0.44 | 0.07 | 0.80 |
| Noll 2007 | Externalizing problems | Child Behavior Checklist | parent report | healthy community | 59 | 59 | 0.23 | -0.13 | 0.59 |
| Noll 2007 | Aggressive-disruptive | Revised Class Play | teacher report | healthy community | 59 | 59 | 0.07 | -0.29 | 0.43 |
| Noll 2007 | Aggressive-disruptive | Revised Class Play | peer report | healthy community | 59 | 59 | -0.13 | -0.49 | 0.23 |
| Noll 2007 | Aggressive-disruptive | Revised Class Play | self-report | healthy community | 59 | 59 | -0.31 | -0.67 | 0.06 |
| Potter 2006^b,c^ | Externalizing Problems | Behavior Assessment System for Children | parent report | normative data | 60 | 60 | 0.27 | -0.08 | 0.63 |
| Pride 2018 | Aggression/defiance | Conners 3 | parent report | healthy community | 19 | 18 | 0.73 | 0.06 | 1.39 |
| Rietman 2017a | Externalizing | Child Behavior Checklist | teacher report | normative data | 38 | 38 | 0.31 | -0.14 | 0.76 |
| Rietman 2017a | Externalizing | Child Behavior Checklist | parent report | normative data | 38 | 38 | 0.45 | 0.00 | 0.91 |
| Rietman 2017b | Externalizing problems | Child Behavior Checklist | parent report | normative data | 69 | 69 | 0.45 | 0.12 | 0.79 |
| Rietman 2018 | Externalizing problems | Child Behavior Checklist | parent report | normative data | 183 | 183 | 0.32 | 0.12 | 0.53 |
| Rietman 2018 | Externalizing problems | Child Behavior Checklist | self report | normative data | 183 | 183 | -0.20 | -0.40 | 0.01 |
| Rietman 2018 | Externalizing problems | Child Behavior Checklist | teacher report | normative data | 183 | 183 | 0.22 | 0.01 | 0.42 |
| Sangster 2011 | Externalizing Problems Composite | Behavior Assessment System for Children | parent report | healthy community | 26 | 21 | 0.15 | -0.42 | 0.73 |
| Sharkey 2021 | Externalizing Problems | Child Behavior Checklist | teacher report | normative data | 7 | 7 | 1.00 | -0.12 | 2.12 |
| Sharkey 2021 | Externalizing Problems | Child Behavior Checklist | parent report | normative data | 7 | 7 | 0.80 | -0.29 | 1.90 |
| Taddei 2019 (NF1) | Ext | Child Behavior Checklist | parent report | normative data | 26 | 26 | 0.10 | -0.44 | 0.64 |
| Taddei 2019 (NF1+OPG) | Ext | Child Behavior Checklist | parent report | normative data | 26 | 26 | 0.57 | 0.02 | 1.13 |
| Taddei 2019 (NF1+CT) | Ext | Child Behavior Checklist | parent report | normative data | 19 | 19 | 0.11 | -0.53 | 0.74 |

*Note*. ^a^Study labels are mostly composed of first author’s last name and year of publication; for studies that had subgroups of NF1 participants and in which only subgroup data were used in analysis, study labels also include the NF1 subgroup name as labeled in each study. ^b^These studies included a typically developing control group but did not provide data for it, and thus normative data were used as the control. ^c^This study is an unpublished dissertation, and all other studies are published journal articles. NF1 *N* = sample size of the included NF1 group. Control *N* = sample size of the control group. *LL* = lower limit of 95% confidence interval; *UL* = upper limit of 95% confidence interval.

**Online Resource 14**. Sensitivity Analysis of Effect Size

|  | Funnel Plot Symmetry Test | |  |  | Trim and Fill Analysis | | | | | |  | Robu Meta Regression | | | | |
| --- | --- | --- | --- | --- | --- | --- | --- | --- | --- | --- | --- | --- | --- | --- | --- | --- |
|  | Egger’s Test | |  |  |  |  |  |  |  |  |  |  |  |  |  |  |
|  | Z | *p* |  | *N* of  trimmed  studies | | Filled  *N* | Filled  ES | *p* | 95% CI | *t* |  | *B* | *SE* | *p* | *n* | *k* |
| Depressive symptoms | 0.48 | .634 |  | 18 | | 0 | 0.43 | <0.001 | [0.21, 0.64] | 4.22 |  | 1.04 | 1.84 | 0.592 | 18 | 21 |
| Anxiety symptoms | 0.24 | .813 |  | 22 | | 4 | 0.45 | 0.004 | [0.17, 0.74] | 3.29 |  | 0.43 | 2.19 | 0.851 | 18 | 24 |
| Somatic symptoms | 2.48 | .013 |  | 23 | | 4 | 0.41 | 0.008 | [0.12, 0.69] | 2.93 |  | 4.03 | 2.02 | 0.081 | 19 | 27 |
| Total internalizing symptoms | 1.58 | .113 |  | 49 | | 10 | 0.34 | <0.001 | [0.17, 0.51] | 4.03 |  | 1.87 | 0.72 | 0.032 | 39 | 75 |
| Aggression | 1.22 | .223 |  | 21 | | 0 | 0.33 | 0.013 | [0.08, 0.58] | 2.72 |  | 2.21 | 1.63 | 0.214 | 21 | 33 |
| Delinquency | 1.91 | .056 |  | 31 | | 6 | 0.28 | 0.005 | [0.09, 0.47] | 3.00 |  | 2.28 | 1.22 | 0.091 | 25 | 37 |
| Total externalizing symptoms | 0.68 | .498 |  | 37 | | 4 | 0.20 | <0.001 | [0.09, 0.31] | 3.74 |  | 0.57 | 0.59 | 0.354 | 33 | 47 |

*Note.* Z = standard normal distribution score; *p* = probability value. Filled *N* = number of filled studies; Filled ES = effect size after filling the hypothetical unpublished studies; *t* = *t* value; CI = confidence interval. *B* = estimated regression coefficient; *SE* = standard error; *n* = number of studies; *k* = number of effect sizes.

**Online Resource 15**. Results from Moderation Tests

| Moderator | *n* | *k* | *β* | *SE* | *LL* | *UL* | *df* | *p*-value |
| --- | --- | --- | --- | --- | --- | --- | --- | --- |
| Depressive symptoms |  |  |  |  |  |  |  |  |
| Mean age | 17 | 20 | 0.00 | 0.01 | -0.02 | 0.02 | 7.43 | 0.845 |
| Female % | 17 | 20 | 0.01 | 0.01 | -0.01 | 0.03 | 8.49 | 0.282 |
| Familial NF1 % | 7 | 9 | -0.02 | 0.06 | -0.25 | 0.22 | 2.37 | 0.824 |
| ADHD % | 9 | 11 | -0.01 | 0.01 | -0.03 | 0.01 | 3.94 | 0.321 |
| FSIQ | 6 | 7 | 0.02 | 0.06 | -0.30 | 0.34 | 1.57 | 0.749 |
| Verbal IQ | 4 | 5 | 0.04 | 0.02 | -0.07 | 0.15 | 1.52 | 0.195 |
| Performance IQ | 4 | 5 | 0.07 | 0.03 | -0.16 | 0.29 | 1.32 | 0.231 |
| Adult sample (vs. child sample) | 18 | 21 | 0.00 | 0.25 | -0.53 | 0.53 | 12.68 | 0.992 |
| Normative data (vs. community) | 18 | 21 | -0.23 | 0.22 | -0.71 | 0.25 | 13.19 | 0.321 |
| Self-report (vs. parent report) | 18 | 21 | -0.18 | 0.21 | -0.63 | 0.28 | 14.69 | 0.419 |
| Anxiety symptoms |  |  |  |  |  |  |  |  |
| Mean age | 16 | 22 | 0.00 | 0.01 | -0.04 | 0.03 | 3.29 | 0.895 |
| Female % | 16 | 22 | 0.01 | 0.01 | -0.02 | 0.03 | 6.65 | 0.591 |
| Familial NF1 % | 9 | 14 | -0.03 | 0.02 | -0.13 | 0.07 | 1.40 | 0.227 |
| ADHD % | 7 | 9 | -0.01 | 0.01 | -0.04 | 0.03 | 2.68 | 0.489 |
| FSIQ | 7 | 8 | -0.01 | 0.06 | -0.24 | 0.22 | 2.24 | 0.862 |
| Adult sample (vs. child sample) | 18 | 24 | -0.20 | 0.26 | -0.86 | 0.47 | 5.03 | 0.484 |
| Siblings (vs. community) | 18 | 24 | -0.25 | 0.18 | -0.68 | 0.17 | 7.85 | 0.201 |
| Normative data (vs. community) | 18 | 24 | 0.19 | 0.27 | -0.38 | 0.76 | 14.75 | 0.490 |
| Self-report (vs. parent report) | 18 | 24 | -0.08 | 0.25 | -0.62 | 0.46 | 15.22 | 0.758 |
| Teacher report (vs. parent report) | 18 | 24 | -0.15 | 0.21 | -0.63 | 0.33 | 9.31 | 0.499 |
| Somatic symptoms |  |  |  |  |  |  |  |  |
| Mean age | 18 | 25 | -0.03 | 0.03 | -0.20 | 0.14 | 1.39 | 0.380 |
| Female % | 19 | 27 | -0.02 | 0.01 | -0.05 | 0.01 | 4.56 | 0.169 |
| Familial NF1 % | 10 | 15 | 0.01 | 0.03 | -0.11 | 0.13 | 1.83 | 0.748 |
| ADHD % | 12 | 18 | 0.01 | 0.01 | -0.03 | 0.04 | 3.76 | 0.607 |
| FSIQ | 13 | 20 | -0.01 | 0.02 | -0.08 | 0.06 | 2.70 | 0.626 |
| Verbal IQ | 10 | 15 | 0.00 | 0.01 | -0.07 | 0.07 | 1.62 | 0.785 |
| Performance IQ | 10 | 15 | 0.01 | 0.01 | -0.03 | 0.05 | 1.64 | 0.314 |
| Normative data (vs. community) | 19 | 27 | 0.18 | 0.32 | -0.52 | 0.88 | 12.78 | 0.587 |
| BASC (vs. CBCL) | 19 | 27 | -0.11 | 0.29 | -0.75 | 0.53 | 10.41 | 0.712 |
| Self-report (vs. parent report) | 19 | 27 | -0.76 | 0.60 | -5.81 | 4.29 | 1.22 | 0.395 |
| Teacher report (vs. parent report) | 19 | 27 | -0.09 | 0.15 | -0.45 | 0.27 | 6.51 | 0.557 |
| Total internalizing symptoms |  |  |  |  |  |  |  |  |
| Mean age | 38 | 71 | 0.03 | 0.01 | -0.07 | 0.13 | 1.36 | 0.211 |
| Female % | 39 | 75 | 0.00 | 0.01 | -0.02 | 0.02 | 6.21 | 0.881 |
| Familial NF1 % | 17 | 39 | 0.00 | 0.02 | -0.10 | 0.10 | 1.78 | 0.982 |
| ADHD % | 21 | 47 | 0.01 | 0.01 | -0.01 | 0.04 | 7.24 | 0.288 |
| FSIQ | 26 | 55 | -0.02 | 0.02 | -0.06 | 0.03 | 8.95 | 0.436 |
| **Verbal IQ** | **21** | **44** | **-0.07** | **0.03** | **-0.13** | **0.00** | **7.55** | **0.040** |
| Performance IQ | 21 | 44 | 0.00 | 0.03 | -0.07 | 0.06 | 5.78 | 0.958 |
| Normative data (vs. community) | 39 | 75 | -0.03 | 0.22 | -0.49 | 0.42 | 18.62 | 0.880 |
| BASC (vs. CBCL) | 39 | 75 | -0.29 | 0.18 | -0.67 | 0.08 | 16.78 | 0.114 |
| Self-report (vs. parent report) | 39 | 75 | 0.15 | 0.46 | -1.32 | 1.61 | 2.99 | 0.773 |
| Teacher report (vs. parent report) | 39 | 75 | 0.16 | 0.13 | -0.14 | 0.46 | 9.18 | 0.256 |
| Teacher report (vs. self-report) | 39 | 75 | 0.01 | 0.46 | -1.12 | 1.15 | 5.93 | 0.977 |
| Aggression |  |  |  |  |  |  |  |  |
| Mean age | 20 | 31 | -0.01 | 0.03 | -0.27 | 0.24 | 1.27 | 0.792 |
| Female % | 21 | 33 | -0.02 | 0.01 | -0.03 | 0.00 | 3.26 | 0.080 |
| Familial NF1 % | 12 | 18 | 0.00 | 0.01 | -0.05 | 0.04 | 1.59 | 0.822 |
| **ADHD %** | **13** | **21** | **0.02** | **0.00** | **0.01** | **0.03** | **3.32** | **0.017** |
| FSIQ | 15 | 24 | -0.01 | 0.04 | -0.12 | 0.10 | 4.31 | 0.779 |
| **Verbal IQ** | **12** | **18** | **-0.06** | **0.02** | **-0.11** | **-0.01** | **2.86** | **0.028** |
| Performance IQ | 12 | 18 | 0.02 | 0.05 | -0.14 | 0.18 | 3.50 | 0.713 |
| Normative data (vs. community) | 21 | 33 | 0.51 | 0.26 | -0.05 | 1.07 | 14.86 | 0.072 |
| **BASC (vs. CBCL)** | **21** | **33** | **-0.57** | **0.24** | **-1.10** | **-0.04** | **9.62** | **0.039** |
| Teacher report (vs. parent report) | 21 | 33 | 0.05 | 0.18 | -0.35 | 0.45 | 9.40 | 0.797 |
| Delinquency |  |  |  |  |  |  |  |  |
| Mean age | 24 | 35 | -0.08 | 0.07 | -0.23 | 0.08 | 10.52 | 0.313 |
| Female % | 24 | 35 | 0.00 | 0.01 | -0.02 | 0.01 | 3.22 | 0.715 |
| Familial NF1 % | 10 | 15 | 0.00 | 0.01 | -0.07 | 0.07 | 1.45 | 0.890 |
| ADHD % | 16 | 26 | 0.01 | 0.01 | -0.01 | 0.02 | 6.06 | 0.398 |
| FSIQ | 20 | 31 | 0.01 | 0.02 | -0.04 | 0.06 | 7.22 | 0.693 |
| Verbal IQ | 16 | 25 | -0.01 | 0.03 | -0.09 | 0.07 | 3.46 | 0.674 |
| Performance IQ | 16 | 25 | 0.02 | 0.01 | -0.02 | 0.06 | 3.79 | 0.168 |
| Siblings (vs. community) | 25 | 37 | -0.14 | 0.29 | -0.83 | 0.56 | 6.40 | 0.655 |
| Normative data (vs. community) | 25 | 37 | -0.08 | 0.27 | -0.73 | 0.57 | 6.14 | 0.784 |
| Normative data (vs. siblings) | 25 | 37 | 0.06 | 0.18 | -0.43 | 0.54 | 4.45 | 0.761 |
| Teacher report (vs. parent report) | 25 | 36 | -0.19 | 0.15 | -0.55 | 0.17 | 7.48 | 0.248 |
| Total externalizing symptoms |  |  |  |  |  |  |  |  |
| Mean age | 32 | 45 | 0.05 | 0.04 | -0.04 | 0.14 | 5.18 | 0.242 |
| Female % | 33 | 47 | 0.00 | 0.01 | -0.01 | 0.01 | 5.39 | 0.967 |
| Familial NF1 % | 16 | 22 | 0.00 | 0.01 | -0.03 | 0.03 | 1.78 | 0.799 |
| ADHD % | 19 | 29 | 0.01 | 0.00 | 0.00 | 0.02 | 5.17 | 0.112 |
| FSIQ | 24 | 36 | -0.01 | 0.01 | -0.04 | 0.01 | 9.87 | 0.328 |
| Verbal IQ | 18 | 25 | -0.02 | 0.01 | -0.05 | 0.02 | 5.35 | 0.251 |
| Performance IQ | 18 | 25 | -0.01 | 0.01 | -0.04 | 0.02 | 4.78 | 0.406 |
| Normative data (vs. community) | 33 | 47 | 0.14 | 0.14 | -0.15 | 0.44 | 16.47 | 0.323 |
| BASC (vs. CBCL) | 33 | 47 | -0.19 | 0.15 | -0.52 | 0.13 | 12.74 | 0.223 |
| Teacher report (vs. parent report) | 33 | 46 | 0.02 | 0.09 | -0.17 | 0.21 | 9.98 | 0.846 |

*Notes:* *n* = number of studies; *k* = number of effect sizes; *β* = estimated regression coefficient; *SE* = standard error; *LL* = lower limit of 95% confidence interval; *UL* = upper limit of 95% confidence interval; *df* = degrees of freedom. Female % = percentage of females in the NF1 group; Familial NF1 % = percentage of participants with familial NF1; ADHD % = percentage of NF1 participants with a diagnosis or clinically significant levels of attention-deficit/hyperactivity disorder. FSIQ = full-scale intelligence quotient; IQ = intelligence quotient. Community = healthy community controls; siblings = unaffected sibling controls. BASC = Behavior Assessment System for Children; CBCL = Child Behavior Checklist. Statistically significant results (*p* < .05) were in boldface.

**Online Resource 16**. Mean Effects for Each Group of Categorical Moderators

|  | Hedges’ *g* | *LL* | *UL* | *SE* | *df* | *p*-value | *n* | *k* | Tau^2^ | *I^2^* (%) |
| --- | --- | --- | --- | --- | --- | --- | --- | --- | --- | --- |
| Depressive symptoms |  |  |  |  |  |  |  |  |  |  |
| Published | 0.43 | 0.19 | 0.66 | 0.11 | 15.82 | 0.001 | 17 | 20 | 0.17 | 82.06 |
| Child sample | 0.43 | 0.20 | 0.66 | 0.10 | 9.77 | 0.002 | 11 | 14 | 0.09 | 71.42 |
| Adult sample | 0.43 | -0.10 | 0.97 | 0.22 | 5.97 | 0.094 | 7 | 7 | 0.32 | 88.94 |
| Community healthy controls | 0.52 | 0.22 | 0.83 | 0.13 | 8.82 | 0.004 | 10 | 10 | 0.14 | 77.44 |
| Normative data as controls | 0.30 | -0.14 | 0.73 | 0.18 | 5.97 | 0.146 | 7 | 9 | 0.17 | 83.51 |
| BASC | 0.31 | 0.10 | 0.52 | 0.09 | 5.77 | 0.012 | 7 | 10 | 0.03 | 36.88 |
| Parent report | 0.54 | 0.26 | 0.82 | 0.12 | 7.83 | 0.002 | 9 | 9 | 0.09 | 67.18 |
| Self-report | 0.34 | 0.03 | 0.65 | 0.14 | 9.90 | 0.037 | 11 | 11 | 0.18 | 83.51 |
| Anxiety symptoms |  |  |  |  |  |  |  |  |  |  |
| Published | 0.27 | -0.01 | 0.56 | 0.13 | 15.80 | 0.057 | 17 | 23 | 0.21 | 84.87 |
| Child sample | 0.32 | -0.01 | 0.65 | 0.15 | 12.83 | 0.056 | 14 | 19 | 0.23 | 84.47 |
| Adult sample | 0.13 | -0.55 | 0.80 | 0.21 | 2.99 | 0.592 | 4 | 5 | 0.17 | 84.33 |
| Community healthy controls | 0.20 | -0.22 | 0.62 | 0.18 | 7.84 | 0.303 | 9 | 11 | 0.21 | 82.56 |
| Normative data as controls | 0.39 | -0.07 | 0.85 | 0.20 | 6.98 | 0.086 | 8 | 11 | 0.24 | 87.75 |
| BASC | -0.01 | -0.41 | 0.39 | 0.16 | 5.95 | 0.943 | 7 | 10 | 0.13 | 73.63 |
| Parent report | 0.30 | -0.11 | 0.71 | 0.18 | 9.97 | 0.135 | 11 | 11 | 0.29 | 87.00 |
| Self-report | 0.19 | -0.09 | 0.48 | 0.12 | 7.57 | 0.151 | 9 | 12 | 0.09 | 71.19 |
| Somatic symptoms |  |  |  |  |  |  |  |  |  |  |
| Published | 0.57 | 0.28 | 0.85 | 0.13 | 16.76 | 0.001 | 18 | 26 | 0.25 | 83.43 |
| Child sample | 0.62 | 0.38 | 0.86 | 0.11 | 16.54 | 0.000 | 18 | 26 | 0.16 | 75.85 |
| Community healthy controls | 0.48 | -0.20 | 1.16 | 0.28 | 5.95 | 0.131 | 7 | 8 | 0.34 | 85.68 |
| Normative data as controls | 0.65 | 0.25 | 1.05 | 0.17 | 7.85 | 0.006 | 9 | 14 | 0.24 | 84.53 |
| CBCL | 0.67 | 0.38 | 0.96 | 0.13 | 9.54 | 0.001 | 11 | 18 | 0.13 | 73.14 |
| BASC | 0.58 | -0.11 | 1.27 | 0.27 | 4.96 | 0.082 | 6 | 7 | 0.28 | 83.80 |
| Parent report | 0.65 | 0.41 | 0.90 | 0.12 | 16.60 | 0.000 | 18 | 19 | 0.17 | 77.73 |
| Teacher report | 0.59 | 0.38 | 0.80 | 0.06 | 2.72 | 0.004 | 6 | 6 | 0.00 | 0.00 |
| Total internalizing symptoms | | | | | | | | | | |
| Published | 0.51 | 0.32 | 0.69 | 0.09 | 34.41 | 0.000 | 36 | 72 | 0.26 | 86.19 |
| Child sample | 0.50 | 0.33 | 0.67 | 0.08 | 35.22 | 0.000 | 37 | 73 | 0.21 | 83.18 |
| Community healthy controls | 0.53 | 0.10 | 0.96 | 0.19 | 9.94 | 0.020 | 11 | 16 | 0.36 | 87.71 |
| Normative data as controls | 0.49 | 0.28 | 0.70 | 0.10 | 24.45 | 0.000 | 26 | 51 | 0.23 | 85.43 |
| CBCL | 0.54 | 0.34 | 0.75 | 0.10 | 25.36 | 0.000 | 27 | 60 | 0.21 | 81.37 |
| BASC | 0.25 | -0.08 | 0.58 | 0.15 | 8.92 | 0.125 | 10 | 13 | 0.18 | 84.83 |
| Parent report | 0.49 | 0.31 | 0.67 | 0.09 | 36.33 | 0.000 | 38 | 54 | 0.24 | 84.87 |
| Self-report | 0.45 | -0.59 | 1.49 | 0.33 | 3.00 | 0.261 | 4 | 6 | 0.32 | 94.66 |
| Teacher report | 0.62 | 0.42 | 0.82 | 0.08 | 6.00 | 0.000 | 9 | 15 | 0.02 | 31.98 |
| Aggression |  |  |  |  |  |  |  |  |  |  |
| Published | 0.34 | 0.08 | 0.60 | 0.13 | 18.78 | 0.015 | 20 | 31 | 0.24 | 82.77 |
| Child sample | 0.36 | 0.11 | 0.62 | 0.12 | 18.71 | 0.007 | 20 | 30 | 0.21 | 80.97 |
| Community healthy controls | 0.03 | -0.44 | 0.50 | 0.20 | 6.93 | 0.881 | 8 | 12 | 0.26 | 81.12 |
| Normative data as controls | 0.54 | 0.15 | 0.93 | 0.17 | 8.85 | 0.013 | 10 | 16 | 0.22 | 82.67 |
| CBCL | 0.48 | 0.23 | 0.74 | 0.12 | 11.48 | 0.001 | 13 | 21 | 0.12 | 71.55 |
| BASC | -0.07 | -0.61 | 0.46 | 0.21 | 4.95 | 0.735 | 6 | 8 | 0.20 | 76.87 |
| Parent report | 0.40 | 0.13 | 0.67 | 0.13 | 18.78 | 0.005 | 20 | 21 | 0.25 | 83.33 |
| Teacher report | 0.42 | 0.08 | 0.76 | 0.14 | 6.56 | 0.022 | 8 | 8 | 0.10 | 69.04 |
| Delinquency |  |  |  |  |  |  |  |  |  |  |
| Published | 0.43 | 0.26 | 0.60 | 0.08 | 23.05 | 0.000 | 25 | 37 | 0.10 | 67.86 |
| Child sample | 0.43 | 0.26 | 0.60 | 0.08 | 23.05 | 0.000 | 25 | 37 | 0.10 | 67.86 |
| Community healthy controls | 0.53 | -0.21 | 1.27 | 0.26 | 3.90 | 0.116 | 5 | 6 | 0.21 | 76.78 |
| Unaffected siblings as controls | 0.34 | -0.20 | 0.88 | 0.16 | 2.79 | 0.132 | 4 | 6 | 0.04 | 37.57 |
| Normative data as controls | 0.42 | 0.20 | 0.64 | 0.10 | 14.49 | 0.001 | 16 | 25 | 0.11 | 71.02 |
| Parent report | 0.45 | 0.28 | 0.61 | 0.08 | 22.96 | 0.000 | 25 | 28 | 0.09 | 66.08 |
| Teacher report | 0.27 | -0.06 | 0.60 | 0.13 | 5.51 | 0.092 | 7 | 7 | 0.06 | 61.96 |
| Total externalizing symptoms | |  |  |  |  |  |  |  |  |  |
| Published | 0.24 | 0.13 | 0.35 | 0.06 | 28.40 | 0.000 | 32 | 46 | 0.05 | 53.95 |
| Child sample | 0.24 | 0.13 | 0.35 | 0.05 | 29.23 | 0.000 | 33 | 47 | 0.05 | 52.50 |
| Community healthy controls | 0.16 | -0.14 | 0.45 | 0.13 | 8.80 | 0.262 | 10 | 14 | 0.12 | 69.33 |
| Normative data as controls | 0.29 | 0.17 | 0.41 | 0.06 | 17.18 | 0.000 | 21 | 29 | 0.03 | 42.26 |
| CBCL | 0.29 | 0.18 | 0.40 | 0.05 | 18.32 | 0.000 | 23 | 33 | 0.02 | 31.54 |
| BASC | 0.09 | -0.25 | 0.42 | 0.14 | 6.90 | 0.555 | 8 | 9 | 0.11 | 75.30 |
| Parent report | 0.27 | 0.16 | 0.38 | 0.05 | 28.84 | 0.000 | 33 | 34 | 0.04 | 47.99 |
| Teacher report | 0.23 | 0.09 | 0.36 | 0.05 | 4.73 | 0.008 | 10 | 10 | 0.00 | 0.00 |

*Notes:* *LL* = lower limit of 95% confidence interval; *UL* = upper limit of 95% confidence interval; *SE* = standard error; *df* = degrees of freedom; *n* = number of studies; *k* = number of effect sizes; Tao^2^ = Tau-squared; *I*^2^ = I-squared. Published = published journal articles. BASC = Behavior Assessment System for Children; CBCL = Child Behavior Checklist.

**Online Resource 17**. Funnel Plots for Effect Sizes of Internalizing Symptoms


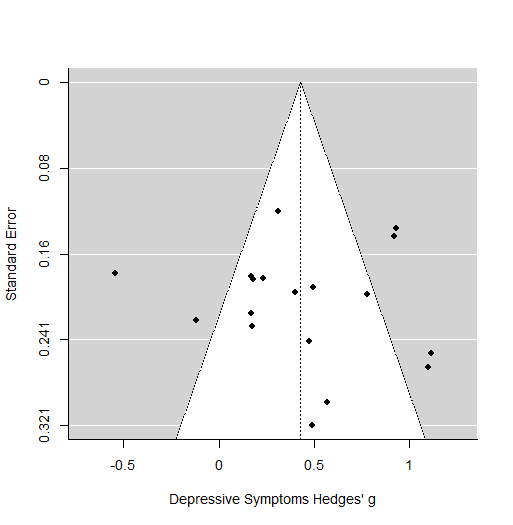

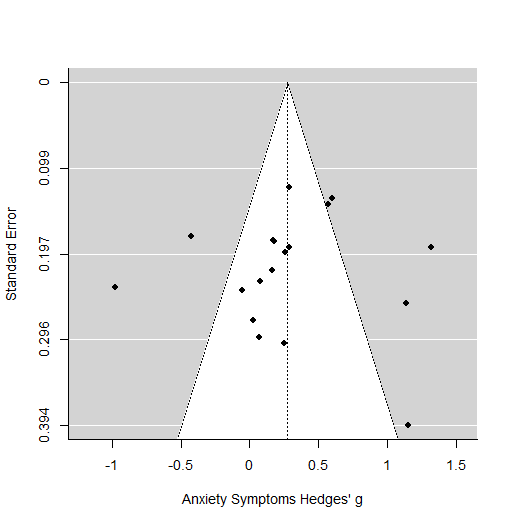


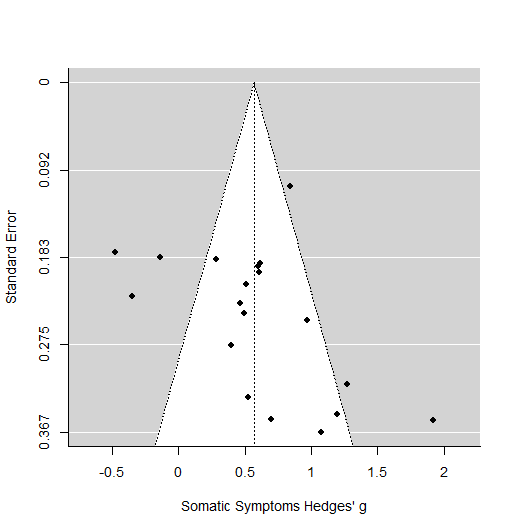

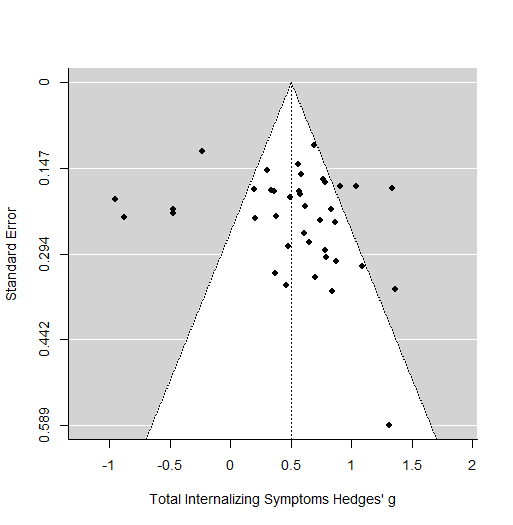


**Online Resource 18**. Funnel Plots for Effect Sizes of Externalizing Symptoms


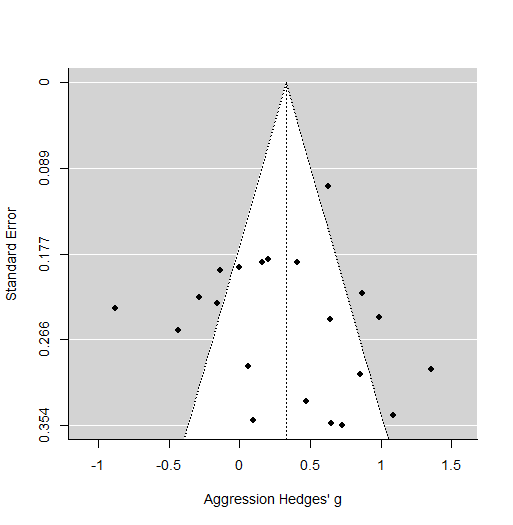


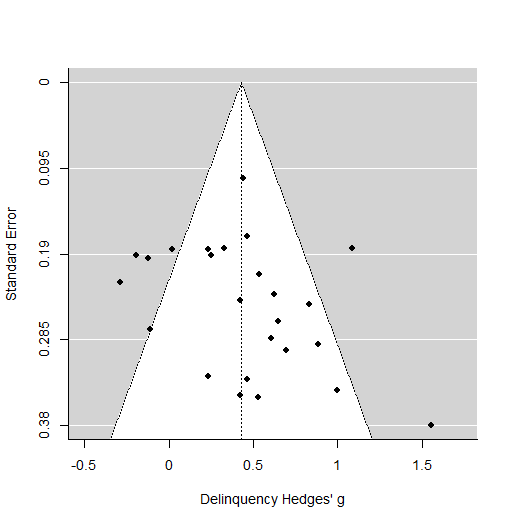

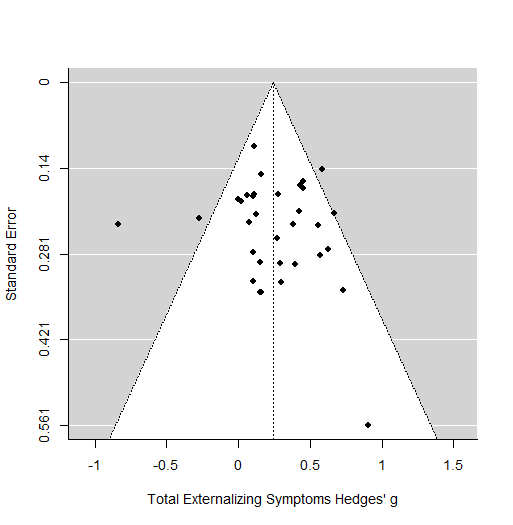


**References**

Acosta, M. T., Kardel, P. G., Walsh, K. S., Rosenbaum, K. N., Gioia, G. A., & Packer, R. J. (2011). Lovastatin as treatment for neurocognitive deficits in neurofibromatosis type 1: phase I study. *Pediatric Neurology*, *45*(4), 241-245.

Allen, T., Willard, V., Anderson, L., Hardy, K., & Bonner, M. (2016). Social functioning and facial expression recognition in children with neurofibromatosis type 1. *Journal of Intellectual Disability Research*, *60*(3), 282-293.

Allen, T. M., Struemph, K. L., Toledo‐Tamula, M. A., Wolters, P. L., Baldwin, A., Widemann, B., & Martin, S. (2018). The relationship between heart rate variability, psychological flexibility, and pain in neurofibromatosis type 1. *Pain Pract*, *18*(8), 969-978. https://doi.org/10.1111/papr.12695

Altman, D., Machin, D., Bryant, T., & Gardner, M. (2013). *Statistics with confidence: Confidence intervals and statistical guidelines*. John Wiley & Sons.

Amitay, E. L., & Keinan-Boker, L. (2015). Breastfeeding and childhood leukemia incidence: A meta-analysis and systematic review. *JAMA Pediatrics*, *169*(6), e151025-e151025. https://doi.org/10.1001/jamapediatrics.2015.1025

Barton, B., & North, K. (2004). Social skills of children with neurofibromatosis type 1. *Developmental Medicine and Child neurology*, *46*(8), 553-563. https://doi.org/10.1017/S0012162204000921

Barton, B., & North, K. (2007). The self-concept of children and adolescents with neurofibromatosis type 1. *Child Care Health Dev*, *33*(4), 401-408. https://doi.org/10.1111/j.1365-2214.2006.00717.x

Bawden, H., Dooley, J., Buckley, D., Camfield, P., Gordon, K., Riding, M., & Llewellyn, G. (1996). MRI and nonverbal cognitive deficits in children with neurofibromatosis 1. *Journal of Clinical and Experimental Neuropsychology*, *18*(6), 784-792. https://doi.org/10.1080/01688639608408302

Berardelli, I., Maraone, A., Belvisi, D., Pasquini, M., Giustini, S., Miraglia, E., Iacovino, C., Pompili, M., Frascarelli, M., & Fabbrini, G. (2021). The importance of suicide risk assessment in patients affected by neurofibromatosis. *Int J Psychiatry Clin Pract*, *25*(4), 350-355. https://doi.org/10.1080/13651501.2021.1921217

Biotteau, M., Déjean, S., Lelong, S., Iannuzzi, S., Faure-Marie, N., Castelnau, P., Rivier, F., Lauwers-Cancès, V., Baudou, E., & Chaix, Y. (2020). Sporadic and familial variants in NF1: An explanation of the wide variability in neurocognitive phenotype? *Front Neurol*, *11*, 368-368. https://doi.org/10.3389/fneur.2020.00368

Bottesi, G., Spoto, A., Trevisson, E., Zuccarello, D., Vidotto, G., Cassina, M., & Clementi, M. (2020). Dysfunctional coping is related to impaired skin‐related quality of life and psychological distress in patients with neurofibromatosis type 1 with major skin involvement. *British Journal of Dermatology*, *182*(6), 1449-1457. https://doi.org/10.1111/bjd.18363

Bulgheroni, S., Taddei, M., Saletti, V., Esposito, S., Micheli, R., & Riva, D. (2019). Visuoperceptual impairment in children with NF1: From early visual processing to procedural strategies. *Behavioural Neurology*, *2019*, 7146168-7146110. https://doi.org/10.1155/2019/7146168

Buono, F. D., Sprong, M. E., Paul, E., Martin, S., Larkin, K., & Garakani, A. (2021). The mediating effects of quality of life, depression, and generalized anxiety on perceived barriers to employment success for people diagnosed with neurofibromatosis type 1. *Orphanet J Rare Dis*, *16*(1), 234. https://doi.org/10.1186/s13023-021-01866-6

Chisholm, A. K., Haebich, K. M., Pride, N. A., Walsh, K. S., Lami, F., Ure, A., Maloof, T., Brignell, A., Rouel, M., Granader, Y., Maier, A., Barton, B., Darke, H., Dabscheck, G., Anderson, V. A., Williams, K., North, K. N., & Payne, J. M. (2022). Delineating the autistic phenotype in children with neurofibromatosis type 1. *Molecular autism*, *13*(1), 3-3. https://doi.org/10.1186/s13229-021-00481-3

Chisholm, A. K., Lami, F., Haebich, K. M., Ure, A., Brignell, A., Maloof, T., Pride, N. A., Walsh, K. S., Maier, A., Rouel, M., Granader, Y., Barton, B., Darke, H., Fuelscher, I., Dabscheck, G., Anderson, V. A., Williams, K., North, K. N., & Payne, J. M. (2023). Sex- and age-related differences in autistic behaviours in children with neurofibromatosis type 1. *Journal of Autism and Developmental Disorders*, *53*(7), 2835-2850. https://doi.org/10.1007/s10803-022-05571-6

Cipolletta, S., Spina, G., & Spoto, A. (2018). Psychosocial functioning, self‐image, and quality of life in children and adolescents with neurofibromatosis type 1. *Child : Care, Health & Development*, *44*(2), 260-268. https://doi.org/10.1111/cch.12496

Cohen, J. (2013). *Statistical power analysis for the behavioral sciences*. Routledge.

Cohen, J. S., Levy, H. P., Sloan, J., Dariotis, J., & Biesecker, B. B. (2015). Depression among adults with neurofibromatosis type 1: prevalence and impact on quality of life. *Clinical Genetics*, *88*(5), 425-430. https://doi.org/10.1111/cge.12551

Cohen, R., Steinberg, T., Kornreich, L., Aharoni, S., Halevy, A., & Shuper, A. (2015). Brain imaging findings and social/emotional problems in Israeli children with neurofibromatosis type 1. *European Journal of Pediatrics*, *174*(2), 199-203. https://doi.org/10.1007/s00431-014-2366-7

Coutinho, V., Câmara-Costa, H., Kemlin, I., Billette de Villemeur, T., Rodriguez, D., & Dellatolas, G. (2017). The discrepancy between performance-based measures and questionnaires when assessing clinical outcomes and quality of life in pediatric patients with neurological disorders. *Applied Neuropsychology: Child*, *6*(4), 255-261. https://doi.org/10.1080/21622965.2016.1146141

Coutinho, V., Kemlin, I., Dorison, N., Billette de Villemeur, T., Rodriguez, D., & Dellatolas, G. (2016). Neuropsychological evaluation and parental assessment of behavioral and motor difficulties in children with neurofibromatosis type 1. *Res. Dev. Disabil.*, *48*, 220-230. https://doi.org/10.1016/j.ridd.2015.11.010

Crook, A., Kwa, R., Ephraums, S., Wilding, M., Thiyagarajan, L., Fleming, J., Moore, K., & Berman, Y. (2022). The psychological impact and experience of breast cancer screening in young women with an increased risk of breast cancer due to neurofibromatosis type 1. *Familial Cancer*, *21*(2), 241-253. https://doi.org/10.1007/s10689-021-00259-9

de Blank, P., Li, N., Fisher, M. J., Ullrich, N. J., Bhatia, S., Yasui, Y., Sklar, C. A., Leisenring, W., Howell, R., Oeffinger, K., Hardy, K., Okcu, M. F., Gibson, T. M., Robison, L. L., Armstrong, G. T., & Krull, K. R. (2020). Late morbidity and mortality in adult survivors of childhood glioma with neurofibromatosis type 1: report from the Childhood Cancer Survivor Study. *Genet Med*, *22*(11), 1794-1802. https://doi.org/10.1038/s41436-020-0873-7

Descheemaeker, M. J., Ghesquière, P., Symons, H., Fryns, J. P., & Legius, E. (2005). Behavioural, academic and neuropsychological profile of normally gifted neurofibromatosis type 1 children. *Journal of Intellectual Disability Research*, *49*(1), 33-46. https://doi.org/10.1111/j.1365-2788.2005.00660.x

Dilts, C. V., Carey, J. C., Kircher, J. C., Hoffman, R. O., Creel, D., Ward, K., Clark, E., & Leonard, C. O. (1996). Children and adolescents with neurofibromatosis 1: A behavioral phenotype. *Journal of Developmental & Behavioral Pediatrics*, *17*(4), 229-239.

Doser, K., Andersen, E. W., Kenborg, L., Dalton, S. O., Jepsen, J. R. M., Kroyer, A., Ostergaard, J., Hove, H., Sorensen, S. A., Johansen, C., Mulvihill, J., Winther, J. F., & Bidstrup, P. E. (2020). Clinical characteristics and quality of life, depression, and anxiety in adults with neurofibromatosis type 1: A nationwide study. *Am J Med Genet A*, *182*(7), 1704-1715. https://doi.org/10.1002/ajmg.a.61627

Eby, N. S., Griffith, J. L., Gutmann, D. H., & Morris, S. M. (2019). Adaptive functioning in children with neurofibromatosis type 1: Relationship to cognition, behavior, and magnetic resonance imaging. *Dev Med Child Neurol 61*(8), 972-978. https://doi.org/10.1111/dmcn.14144

Ferner, R. E., Hughes, R. A., & Weinman, J. (1996). Intellectual impairment in neurofibromatosis 1. *J Neurol Sci*, *138*(1-2), 125-133. https://doi.org/10.1016/0022-510x(96)00022-6

Fishbein, N. S., Vranceanu, A.-M., & Mace, R. A. (2022). Baseline characteristics of adults with neurofibromatosis enrolled on a psychosocial randomized controlled trial. *Journal of Neuro-Oncology*, *159*(3), 637-646. https://doi.org/10.1007/s11060-022-04104-6

Fisher, Z., & Tipton, E. (2015). robumeta: An R-package for robust variance estimation in meta-analysis. *arXiv.org*. https://doi.org/10.48550/arxiv.1503.02220

Foy, A. M. H., Hudock, R. L., Shanley, R., & Pierpont, E. I. (2022). Social behavior in RASopathies and idiopathic autism. *Journal of Neurodevelopmental Disorders*, *14*(1), 5-5. https://doi.org/10.1186/s11689-021-09414-w

Fu, R., Gartlehner, G., Grant, M., Shamliyan, T., Sedrakyan, A., Wilt, T. J., Griffith, L., Oremus, M., Raina, P., Ismaila, A., Santaguida, P., Lau, J., & Trikalinos, T. A. (2011). Conducting quantitative synthesis when comparing medical interventions: AHRQ and the Effective Health Care Program. *Journal of Clinical Epidemiology*, *64*(11), 1187-1197. https://doi.org/10.1016/j.jclinepi.2010.08.010

Galasso, C., Lo-Castro, A., Di Carlo, L., Pitzianti, M. B., D’Agati, E., Curatolo, P., & Pasini, A. (2014). Planning deficit in children with neurofibromatosis type 1:A neurocognitive trait independent from attention-deficit hyperactivity disorder (ADHD)? *Journal of Child Neurology*, *29*(10), 1320-1326. https://doi.org/10.1177/0883073813517001

Garg, S. (2015). *Autism spectrum disorder in neufibromatosis type 1: Prevalence and characterisation of the phenotype* (Publication Number 28301622) [Doctoral Dissertation, University of Manchester]. ProQuest Dissertations and Theses Global.

Garg, S., Lehtonen, A., Huson, S. M., Emsley, R., Trump, D., Evans, D. G., & Green, J. (2013). Autism and other psychiatric comorbidity in neurofibromatosis type 1: evidence from a population-based study. *Developmental Medicine & Child Neurology*, *55*(2), 139-145. https://doi.org/10.1111/dmcn.12043

Garwood, M. M., Bernacki, J. M., Fine, K. M., Hainsworth, K. R., Davies, W. H., & Klein-Tasman, B. P. (2012). Physical, cognitive, and psychosocial predictors of functional disability and health-related quality of life in adolescents with neurofibromatosis-1. *Pain Res Treat*, *2012*, 975364. https://doi.org/10.1155/2012/975364

Gilboa, Y., Rosenblum, S., Fattal-Valevski, A., Toledano-Alhadef, H., Rizzo, A. S., & Josman, N. (2011). Using a Virtual Classroom environment to describe the attention deficits profile of children with neurofibromatosis type 1. *Res Dev Disabil*, *32*(6), 2608-2613. https://doi.org/10.1016/j.ridd.2011.06.014

Gordon, M., Lumley, T., & Gordon, M. M. (2019). *Package ‘forestplot’*. The Comprehensive R Archive Network. https://www.rdocumentation.org/packages/forestplot/versions/3.1.3

Graf, A., Landolt, M. A., Mori, A. C., & Boltshauser, E. (2006). Quality of life and psychological adjustment in children and adolescents with neurofibromatosis type 1. *The Journal of Pediatrics*, *149*(3), 348-353. https://doi.org/10.1016/j.jpeds.2006.04.025

Gray, L. S. (2014). *Family functioning and coping: Mediators and moderators between severity and internalizing disorders for children with nf1* (Publication Number 3635151) [Doctoral Dissertation, The George Washington University]. ProQuest Dissertations and Theses Global.

Hamoy-Jimenez, G., Elahmar, H. A., Mendoza, M., Kim, R. H., Bril, V., & Barnett, C. (2022). A cross-sectional study of gender differences in quality of life domains in patients with neurofibromatosis type 1. *Orphanet J Rare Dis*, *17*(1), 40. https://doi.org/10.1186/s13023-022-02195-y

Hardy, K. K., Berger, C., Griffin, D., Walsh, K. S., Sharkey, C. M., Weisman, H., Gioia, A., Packer, R. J., & Acosta, M. T. (2021). Computerized working memory training for children with neurofibromatosis type 1 (NF1): A pilot study. *J Child Neurol*, *36*(12), 1078-1085. https://doi.org/10.1177/08830738211038083

Hedges, L. V., & Olkin, I. (2014). *Statistical methods for meta-analysis*. Academic press.

Heimgärtner, M., Granström, S., Haas-Lude, K., Leark, R. A., Mautner, V.-F., & Lidzba, K. (2019). Attention deficit predicts intellectual functioning in children with neurofibromatosis type 1. *Int. J. Pediatr.*, *2019*, 9493837-9493810. https://doi.org/10.1155/2019/9493837

Hellebrekers, D. M. J., van Abeelen, S. A. M., Catsman, C. E., van Kuijk, S. M. J., Laridon, A. M., Klinkenberg, S., Hendriksen, J. G. M., & Vles, J. S. H. (2022). Cognitive and behavioral functioning in two neurogenetic disorders; how different are these aspects in Duchenne muscular dystrophy and neurofibromatosis type 1? *PloS one*, *17*(10), e0275803. https://doi.org/10.1371/journal.pone.0275803

Higgins, J. P. T., Li, T., & Deeks, J. J. (2019). Choosing effect measures and computing estimates of effect. In (pp. 143-176). John Wiley & Sons, Ltd. https://doi.org/10.1002/9781119536604.ch6

Hou, Y., Wu, X., Liu, D., Martin, S., Toledo-Tamula, M. A., Allen, T., Baldwin, A., Gillespie, A., Goodwin, A., Widemann, B. C., & Wolters, P. L. (2022). Demographic and disease-related predictors of socioemotional development in children with neurofibromatosis type 1 and plexiform neurofibromas: An exploratory study. *Cancers*, *14*(23), 5956. https://doi.org/10.3390/cancers14235956

Huijbregts, S., Swaab, H., & de Sonneville, L. (2010). Cognitive and motor control in neurofibromatosis type I: Influence of maturation and hyperactivity-inattention. *Dev Neuropsychol*, *35*(6), 737-751. https://doi.org/10.1080/87565641.2010.508670

Huijbregts, S. C., & de Sonneville, L. M. (2011). Does cognitive impairment explain behavioral and social problems of children with neurofibromatosis type 1? *Behav Genet*, *41*(3), 430-436. https://doi.org/10.1007/s10519-010-9430-5

Isenberg, J. C., Templer, A., Gao, F., Titus, J. B., & Gutmann, D. H. (2013). Attention skills in children with neurofibromatosis type 1. *J Child Neurol*, *28*(1), 45-49. https://doi.org/10.1177/0883073812439435

Johnson, H., Wiggs, L., Stores, G., & Huson, S. M. (2005). Psychological disturbance and sleep disorders in children with neurofibromatosis type 1. *Dev Med Child Neurol*, *47*(4), 237-242. https://doi.org/10.1017/s0012162205000460

Johnson, N. S., Saal, H. M., Lovell, A. M., & Schorry, E. K. (1999). Social and emotional problems in children with neurofibromatosis type 1: Evidence and proposed interventions. *J Pediatr*, *134*(6), 767-772. https://doi.org/10.1016/S0022-3476(99)70296-9

Kenborg, L., Andersen, E. W., Duun-Henriksen, A. K., Jepsen, J. R. M., Doser, K., Dalton, S. O., Bidstrup, P. E., Kroyer, A., Frederiksen, L. E., Johansen, C., Ostergaard, J. R., Hove, H., Sorensen, S. A., Riccardi, V. M., Mulvihill, J. J., & Winther, J. F. (2021). Psychiatric disorders in individuals with neurofibromatosis 1 in Denmark: A nationwide register-based cohort study. *Am J Med Genet A*, *185*(12), 3706-3716. https://doi.org/10.1002/ajmg.a.62436

Klein-Tasman, B. P., Janke, K. M., Luo, W., Casnar, C. L., Hunter, S. J., Tonsgard, J., Trapane, P., van der Fluit, F., & Kais, L. A. (2014). Cognitive and psychosocial phenotype of young children with neurofibromatosis-1. *Journal of the International Neuropsychological Society*, *20*(1), 88-98. https://doi.org/10.1017/S1355617713001227

Lai, J. S., Jensen, S. E., Charrow, J., & Listernick, R. (2019). Patient reported outcomes measurement information system and quality of life in neurological disorders measurement system to evaluate quality of life for children and adolescents with neurofibromatosis type 1 associated plexiform neurofibroma. *J Pediatr*, *206*, 190-196. https://doi.org/10.1016/j.jpeds.2018.10.019

Lalancette, E., Charlebois-Poirier, A. R., Agbogba, K., Knoth, I. S., Côté, V., Perreault, S., & Lippé, S. (2023). Time-frequency analyses of repetition suppression and change detection in children with neurofibromatosis type 1. *Brain Research*, *1818*. https://doi.org/10.1016/j.brainres.2023.148512

Lalancette, E., Charlebois-Poirier, A. R., Agbogba, K., Knoth, I. S., Jones, E. J. H., Mason, L., Perreault, S., & Lippe, S. (2022). Steady-state visual evoked potentials in children with neurofibromatosis type 1: Associations with behavioral rating scales and impact of psychostimulant medication. *J Neurodev Disord*, *14*(1), 42. https://doi.org/10.1186/s11689-022-09452-y

Lee, D. K. (2016). Alternatives to P value: Confidence interval and effect size. *Korean Journal of Anesthesiology*, *69*(6), 555-562. https://doi.org/10.4097/kjae.2016.69.6.555

Leidger, A., Vosschulte, M., Nieder, T. O., & Mautner, V. F. (2022). Sexual self-esteem and psychological burden of adults with neurofibromatosis type 1. *Front Psychol*, *13*, 883019. https://doi.org/10.3389/fpsyg.2022.883019

Lester, E. G., Wang, K. E., Blakeley, J. O., & Vranceanu, A. M. (2023). Occurrence and severity of suicidal ideation in adults with neurofibromatosis participating in a Mind-Body RCT. *Cogn Behav Neurol*, *36*(1), 19-27. https://doi.org/10.1097/WNN.0000000000000332

Lewis, A. K., & Porter, M. A. (2016). Social competence in children with neurofibromatosis type 1: Relationships with psychopathology and cognitive ability. *Journal of Childhood & Developmental Disorders*, *02*(02). https://doi.org/10.4172/2472-1786.100020

Lewis, A. K., Porter, M. A., Williams, T. A., Bzishvili, S., North, K. N., & Payne, J. M. (2017). Facial emotion recognition, face scan paths, and face perception in children with neurofibromatosis type 1. *Neuropsychology*, *31*(4), 361-370. https://doi.org/10.1037/neu0000340

Loitfelder, M., Huijbregts, S. C., Veer, I. M., Swaab, H. S., Van Buchem, M. A., Schmidt, R., & Rombouts, S. A. (2015). Functional connectivity changes and executive and social problems in neurofibromatosis type I. *Brain Connect*, *5*(5), 312-320. https://doi.org/10.1089/brain.2014.0334

Long, S. K. (2001). *Academic and psychosocial functioning of children with neurofibromatosis -type 1: A longitudinal study using individual growth curves* (Publication Number 3021368) [Doctoral Dissertation, University of Houston]. ProQuest Dissertations and Theses Global.

Lorenzo, J., Barton, B., Acosta, M. T., & North, K. (2011). Mental, motor, and language development of toddlers with neurofibromatosis type 1. *The Journal of Pediatrics*, *158*(4), 660-665. https://doi.org/10.1016/j.jpeds.2010.10.001

Lorenzo, J., Barton, B., Arnold, S. S., & North, K. N. (2013). Cognitive features that distinguish preschool-age children with neurofibromatosis type 1 from their peers: A matched case-control study. *The Journal of Pediatrics*, *163*(5), 1479-1483.e1471. https://doi.org/10.1016/j.jpeds.2013.06.038

Mace, R. A., Doorley, J., Bakhshaie, J., Cohen, J. E., & Vranceanu, A.-M. (2021). Psychological resiliency explains the relationship between emotional distress and quality of life in neurofibromatosis. *J Neurooncol*, *155*(2), 125-132. https://doi.org/10.1007/s11060-021-03852-1

Martin, S., Allen, T., Toledo-Tamula, M. A., Struemph, K., Reda, S., Wolters, P. L., Baldwin, A., Quinn, M., & Widemann, B. C. (2021). Acceptance and commitment therapy for adolescents and adults with neurofibromatosis type 1, plexiform neurofibromas, and chronic pain: results of a randomized controlled trial. *J Contextual Behav Sci*, *22*, 93-101. https://doi.org/10.1016/j.jcbs.2021.10.003

Martin, S., Wolters, P., Baldwin, A., Gillespie, A., Dombi, E., Walker, K., & Widemann, B. (2012). Social-emotional functioning of children and adolescents with neurofibromatosis type 1 and plexiform neurofibromas: Relationships with cognitive, disease, and environmental variables. *J Pediatr Psychol*, *37*(7), 713-724. https://doi.org/10.1093/jpepsy/jsr124

Martin, S., Wolters, P. L., Toledo-Tamula, M. A., Schmitt, S. N., Baldwin, A., Starosta, A., Gillespie, A., & Widemann, B. (2016). Acceptance and commitment therapy in youth with neurofibromatosis type 1 (NF1) and chronic pain and their parents: a pilot study of feasibility and preliminary efficacy. *Am J Med Genet A*, *170*(6), 1462-1470. https://doi.org/10.1002/ajmg.a.37623

Mautner, V.-F., Granström, S., & Leark, R. A. (2015). Impact of ADHD in adults with neurofibromatosis type 1:Associated psychological and social problems. *Journal of Attention Disorders*, *19*(1), 35-43. https://doi.org/10.1177/1087054712450749

Mautner, V.-F., Kluwe, L., Thakker, S. D., & Leark, R. A. (2002). Treatment of ADHD in neurofibromatosis type 1. *Dev Med Child Neurol 44*(3), 164-170.

McCurdy, M. D. (2019). *Social competence in youth with neurofibromatosis type 1* (Publication Number 13903256) [Doctoral Disseration, Drexel University]. ProQuest Dissertations and Theses Global.

McNeill, A. M., Hudock, R. L., Foy, A. M. H., Shanley, R., Semrud‐Clikeman, M., Pierpont, M. E., Berry, S. A., Sommer, K., Moertel, C. L., & Pierpont, E. I. (2019). Emotional functioning among children with neurofibromatosis type 1 or Noonan syndrome. *Am J Med Genet A*, *179*(12), 2433-2446. https://doi.org/10.1002/ajmg.a.61361

Merchant, T. E., Conklin, H. M., Wu, S., Lustig, R. H., & Xiong, X. (2009). Late effects of conformal radiation therapy for pediatric patients with low-grade glioma: prospective evaluation of cognitive, endocrine, and hearing deficits. *J Clin Oncol*, *27*(22), 3691-3697. https://doi.org/10.1200/JCO.2008.21.2738

Min, K., Hong, D. W., Kim, E. K., & Lee, B. H. (2020). Psychological characteristics of adult neurofibromatosis type 1 patients seeking elective surgery. *Archives of Aesthetic Plastic Surgery*, *26*(4), 150-156. https://doi.org/10.14730/aaps.2020.02187

Morotti, H., Mastel, S., Keller, K., Barnard, R. A., Hall, T., O'Roak, B. J., & Fombonne, E. (2021). Autism and attention‐deficit/hyperactivity disorders and symptoms in children with neurofibromatosis type 1. *Dev Med Child Neurol 63*(2), 226-232. https://doi.org/10.1111/dmcn.14558

Morris, S. M., Gupta, A., Kim, S., Foraker, R. E., Gutmann, D. H., & Payne, P. R. O. (2021). Predictive modeling for clinical features associated with neurofibromatosis type 1. *Neurology: Clinical Practice*, *11*(6), e497-e505. https://doi.org/10.1212/cpj.0000000000001089

Noll, R. B., Reiter-Purtill, J., Moore, B. D., Schorry, E. K., Lovell, A. M., Vannatta, K., & Gerhardt, C. A. (2007). Social, emotional, and behavioral functioning of children with NF1. *American Journal of Medical Genetics Part A*, *143A*(19), 2261-2273. https://doi.org/10.1002/ajmg.a.31923

North, K., Joy, P., Yuille, D., Cocks, N., & Hutchins, P. (1995). Cognitive function and academic performance in children with neurofibromatosis type 1. *Dev Med Child Neurol 37*(5), 427-436.

Nussbaumer-Streit, B., Klerings, I., Dobrescu, A. I., Persad, E., Stevens, A., Garritty, C., Kamel, C., Affengruber, L., King, V. J., & Gartlehner, G. (2020). Excluding non-English publications from evidence-syntheses did not change conclusions: A meta-epidemiological study. *J Clin Epidemiol*, *118*, 42-54. https://doi.org/10.1016/j.jclinepi.2019.10.011

Parmeggiani, A., Boiani, F., Capponi, S., Duca, M., Angotti, M., Pignataro, V., Sacrato, L., Spinardi, L., Vara, G., Maltoni, L., Cecconi, I., Pastore Trossello, M., & Franzoni, E. (2018). Neuropsychological profile in Italian children with neurofibromatosis type 1 (NF1) and their relationships with neuroradiological data: Preliminary results. *Eur J Paediatr Neurol*, *22*(5), 822-830. https://doi.org/10.1016/j.ejpn.2018.04.016

Pasini, A., Lo-Castro, A., Di Carlo, L., Pitzianti, M., Siracusano, M., Rosa, C., & Galasso, C. (2012). Detecting anxiety symptoms in children and youths with neurofibromatosis type I. *Am J Med Genet B Neuropsychiatr Genet*, *159B*(7), 869-873. https://doi.org/10.1002/ajmg.b.32095

Payne, J. M., Hearps, S. J. C., Walsh, K. S., Paltin, I., Barton, B., Ullrich, N. J., Haebich, K. M., Coghill, D., Gioia, G. A., Cantor, A., Cutter, G., Tonsgard, J. H., Viskochil, D., Rey-Casserly, C., Schorry, E. K., Ackerson, J. D., Klesse, L., Fisher, M. J., Gutmann, D. H., . . . Consortium, N. F. C. T. (2019). Reproducibility of cognitive endpoints in clinical trials: Lessons from neurofibromatosis type 1. *Ann Clin Transl Neurol*, *6*(12), 2555-2565. https://doi.org/10.1002/acn3.50952

Peng, P., Barnes, M., Wang, C., Wang, W., Li, S., Swanson, H. L., Dardick, W., & Tao, S. (2018). A meta-analysis on the relation between reading and working memory. *Psychological Bulletin*, *144*(1), 48-76. https://doi.org/10.1037/bul0000124

Peters, J. L., Sutton, A. J., Jones, D. R., Abrams, K. R., & Rushton, L. (2006). Comparison of two methods to detect publication bias in meta-analysis. *JAMA : The Journal of the American Medical Association*, *295*(6), 676-680. https://doi.org/10.1001/jama.295.6.676

Pierpont, E. I., Hudock, R. L., Foy, A. M., Semrud-Clikeman, M., Pierpont, M. E., Berry, S. A., Shanley, R., Rubin, N., Sommer, K., & Moertel, C. L. (2018). Social skills in children with RASopathies: a comparison of Noonan syndrome and neurofibromatosis type 1. *Journal of Neurodevelopmental Disorders*, *10*(1), 21. https://doi.org/10.1186/s11689-018-9239-8

Potter, B. S. (2006). *Evaluating everyday executive functions and psychosocial behavior in children with neurofibromatosis type I* (Publication Number 3220711) [Doctoral Dissertation, Antioch University]. ProQuest Dissertations and Theses Global.

Pride, N. A., Haebich, K. M., Walsh, K. S., Lami, F., Rouel, M., Maier, A., Chisholm, A. K., Lorenzo, J., Hearps, S. J. C., North, K. N., & Payne, J. M. (2023). Sensory Processing in Children and Adolescents with Neurofibromatosis Type 1. *Cancers (Basel)*, *15*(14). https://doi.org/10.3390/cancers15143612

Pride, N. A., Korgaonkar, M. S., North, K. N., & Payne, J. M. (2018). Impaired engagement of the ventral attention system in neurofibromatosis type 1. *Brain Imaging Behav*, *12*(2), 499-508. https://doi.org/10.1007/s11682-017-9717-8

Rietman, A. B., Oostenbrink, R., Bongers, S., Gaukema, E., van Abeelen, S., Hendriksen, J. G., Looman, C. W. N., de Nijs, P. F. A., & de Wit, M. C. (2017b). Motor problems in children with neurofibromatosis type 1. *J Neurodev Disord*, *9*, 19. https://doi.org/10.1186/s11689-017-9198-5

Rietman, A. B., Oostenbrink, R., van Noort, K., Franken, M.-C., Catsman - Berrevoets, C., Aarsen, F., Heniksen, J. G., & de Nijs, P. (2017a). Development of emotional and behavioral problems in neurofibromatosis type 1 during young childhood. *Am J Med Genet A*, *173*(9), 2373-2380. https://doi.org/10.1002/ajmg.a.38323

Rietman, A. B., Vaart, T., Plasschaert, E., Nicholson, B. A., Oostenbrink, R., Krab, L. C., Descheemaeker, M. J., de Wit, M. C., Moll, H., Legius, E., & de Nijs, P. (2018). Emotional and behavioral problems in children and adolescents with neurofibromatosis type 1. *American Journal of Medical Genetics. Part B, Neuropsychiatric Genetics*, *177*(3), 319-328. https://doi.org/10.1002/ajmg.b.32612

Samuelsson, B., & Riccardi, V. M. (1989). Neurofibromatosis in Gothenburg, Sweden. III. Psychiatric and social aspects. *Neurofibromatosis*, *2*(2), 84-106.

Sangster, J., Shores, E. A., Watt, S., & North, K. N. (2011). The cognitive profile of preschool-aged children with neurofibromatosis type 1. *Child Neuropsychol*, *17*(1), 1-16. https://doi.org/10.1080/09297041003761993

Schrimsher, G. W. (2003). *Neuroanatomical and visual -spatial/motor performance correlates of attention-deficit hyperactivity disorder symptomatology in children with neurofibromatosis type-I and normal children* (Publication Number 3089791) [Doctoral Dissertation, University of Houston]. ProQuest Dissertations and Theses Global.

Sharkey, C. M., Mullins, L. L., Clawson, A. H., Gioia, A., Hawkins, M. A. W., Chaney, J. M., Walsh, K. S., & Hardy, K. K. (2021). Assessing neuropsychological phenotypes of pediatric brain tumor survivors. *Psychooncology*, *30*(8), 1366-1374. https://doi.org/10.1002/pon.5692

Shi, L., & Lin, L. (2019). The trim-and-fill method for publication bias: Practical guidelines and recommendations based on a large database of meta-analyses. *Medicine (Baltimore)*, *98*(23), e15987-e15987. https://doi.org/10.1097/MD.0000000000015987

Stivaros, S., Garg, S., Tziraki, M., Cai, Y., Thomas, O., Mellor, J., Morris, A. A., Jim, C., Szumanska-Ryt, K., Parkes, L. M., Haroon, H. A., Montaldi, D., Webb, N., Keane, J., Castellanos, F. X., Silva, A. J., Huson, S., Williams, S., Gareth Evans, D., . . . Consortium, S. (2018). Randomised controlled trial of simvastatin treatment for autism in young children with neurofibromatosis type 1 (SANTA). *Mol Autism*, *9*, 12. https://doi.org/10.1186/s13229-018-0190-z

Taddei, M., Erbetta, A., Esposito, S., Saletti, V., Bulgheroni, S., & Riva, D. (2019). Brain tumors in NF1 children: Influence on neurocognitive and behavioral outcome. *Cancers*, *11*(11), 1772. https://doi.org/10.3390/cancers11111772

Talaei-Khoei, M., Riklin, E., Merker, V. L., Sheridan, M. R., Jordan, J. T., Plotkin, S. R., & Vranceanu, A. M. (2017). First use of patient reported outcomes measurement information system (PROMIS) measures in adults with neurofibromatosis. *J Neurooncol*, *131*(2), 413-419. https://doi.org/10.1007/s11060-016-2314-7

Tang, H., Wu, Q., Li, S., Fang, Y., Yang, Z., Wang, B., Wang, X., & Liu, P. (2021). Visuospatial but not verbal working memory deficits in adult patients with neurofibromatosis type 1. *Frontiers in Psychology*, *12*, 751384. https://doi.org/10.3389/fpsyg.2021.751384

van der Vaart, T., Plasschaert, E., Rietman, A. B., Renard, M., Oostenbrink, R., Vogels, A., de Wit, M.-C. Y., Descheemaeker, M.-J., Vergouwe, Y., & Catsman-Berrevoets, C. E. (2013). Simvastatin for cognitive deficits and behavioural problems in patients with neurofibromatosis type 1 (NF1-SIMCODA): a randomised, placebo-controlled trial. *Lancet Neurol*, *12*(11), 1076-1083. https://doi.org/10.1016/S1474-4422(13)70227-8

van der Vaart, T., Rietman, A. B., Plasschaert, E., Legius, E., Elgersma, Y., Moll, H. A., & Group, N. S. S. (2016). Behavioral and cognitive outcomes for clinical trials in children with neurofibromatosis type 1. *Neurology*, *86*(2), 154-160. https://doi.org/10.1212/WNL.0000000000002118

van Geel, M., Vedder, P., & Tanilon, J. (2014). Bullying and weapon carrying: A meta-analysis. *JAMA Pediatrics*, *168*(8), 714-720. https://doi.org/10.1001/jamapediatrics.2014.213

Varnhagen, C. K., Lewin, S., Das, J. P., Bowen, P., Ma, K., & Klimek, M. (1988). Neurofibromatosis and psychological processes. *Journal of developmental and behavioral pediatrics : JDBP*, *9*(5), 257-265. https://doi.org/10.1097/00004703-198810000-00003

Vaucheret Paz, E., López Ballent, A., Puga, C., García Basalo, M. J., Baliarda, F., Ekonen, C., Ilari, R., & Agosta, G. (2019). Cognitive profile and disorders affecting higher brain functions in paediatric patients with neurofibromatosis type 1. *Neurología (English Edition)*, *34*(6), 353-359. https://doi.org/10.1016/j.nrleng.2017.02.009

Viechtbauer, W., López-López, J. A., Sánchez-Meca, J., & Marín-Martínez, F. (2015). A comparison of procedures to test for moderators in mixed-effects meta-regression models. *Psychological Methods*, *20*(3), 360-374. https://doi.org/10.1037/met0000023

Viechtbauer, W., & Viechtbauer, M. W. (2015). *Package ‘metafor’*. The Comprehensive R Archive Network. https://cran.r-project.org/web/packages/metafor/metafor.pdf

Vranceanu, A. M., Merker, V. L., Plotkin, S. R., & Park, E. R. (2014). The relaxation response resiliency program (3RP) in patients with neurofibromatosis 1, neurofibromatosis 2, and schwannomatosis: results from a pilot study. *J Neurooncol*, *120*(1), 103-109. https://doi.org/10.1007/s11060-014-1522-2

Walsh, K. S., Velez, J. I., Kardel, P. G., Imas, D. M., Muenke, M., Packer, R. J., Castellanos, F. X., & Acosta, M. T. (2013). Symptomatology of autism spectrum disorder in a population with neurofibromatosis type 1. *Dev Med Child Neurol*, *55*(2), 131-138. https://doi.org/10.1111/dmcn.12038

Wang, D. L., Smith, K. B., Esparza, S., Leigh, F. A., Muzikansky, A., Park, E. R., & Plotkin, S. R. (2012). Emotional functioning of patients with neurofibromatosis tumor suppressor syndrome. *Genet Med*, *14*(12), 977-982. https://doi.org/10.1038/gim.2012.85

Wang, X., Wu, Q., Tang, H., Zhao, F., Yang, Z., Wang, B., Li, P., Wang, Z., Wu, Y., Fan, J., & Liu, P. (2019). Selective impairment of the executive attentional network in adult patients with neurofibromatosis type 1. *NeuroReport*, *30*(14), 921-926. https://doi.org/10.1097/WNR.0000000000001275

Wiener, L., Battles, H., Bedoya, S. Z., Baldwin, A., Widemann, B. C., & Pao, M. (2018). Identifying symptoms of distress in youth living with neurofibromatosis type 1 (NF1). *J Genet Couns*, *27*(1), 115-123. https://doi.org/10.1007/s10897-017-0128-1

Wolters, P. L., Burns, K. M., Martin, S., Baldwin, A., Dombi, E., Toledo-Tamula, M. A., Dudley, W. N., Gillespie, A., & Widemann, B. C. (2015). Pain interference in youth with neurofibromatosis type 1 and plexiform neurofibromas and relation to disease severity, social-emotional functioning, and quality of life. *Am J Med Genet A*, *167*(9), 2103-2113. https://doi.org/10.1002/ajmg.a.37123

Xue, H., Wu, Q., Yang, Z., Wang, B., Wang, X., & Liu, P. (2021). Dissociated deficits between explicit and implicit empathetic pain perception in neurofibromatosis type 1. *Brain Sci*, *11*(12). https://doi.org/10.3390/brainsci11121591

Yamauchi, T., & Suka, M. (2023). Quality of life in patients with neurofibromatosis type 1: a nationwide database study in Japan from 2015 to 2019. *Environ Health Prev Med*, *28*, 77. https://doi.org/10.1265/ehpm.23-00221

Yoo, H. K., Porteous, A., Ng, A., Haria, K., Griffiths, A., Lloyd, A., Yang, X., Kazeem, G., & Barut, V. (2023). Impact of neurofibromatosis type 1 with plexiform neurofibromas on the health-related quality of life and work productivity of adult patients and caregivers in the UK: a cross-sectional survey. *BMC Neurol*, *23*(1), 419. https://doi.org/10.1186/s12883-023-03429-7

Yoshida, Y., Ehara, Y., Koga, M., & Imafuku, S. (2022). Health-related quality of life in patients with neurofibromatosis 1 in Japan: A questionnaire survey using EQ-5D-5L. *J Dermatol*, *49*(12), 1228-1232. https://doi.org/10.1111/1346-8138.16510

Yund, B. D. (2020). *Factors contributing to executive functioning in children with neurofibromatosis type 1* (Publication Number
28087958) [Doctoral Dissertation, University of Wisconsin - Milwaukee]. ProQuest Dissertations and Theses Global.

Zimerman, M., Wessel, M. J., Timmermann, J. E., Granstrom, S., Gerloff, C., Mautner, V. F., & Hummel, F. C. (2015). Impairment of procedural learning and motor intracortical inhibition in neurofibromatosis type 1 patients. *EBioMedicine*, *2*(10), 1430-1437. https://doi.org/10.1016/j.ebiom.2015.08.036

Zöller, M. E., & Rembeck, B. (1999). A psychiatric 12-year follow-up of adult patients with neurofibromatosis type 1. *J Psychiatr Res*, *33*(1), 63-68. https://doi.org/10.1016/s0022-3956(98)00052-1
